# Alloying–realloying enabled high durability for Pt–Pd-3d-transition metal nanoparticle fuel cell catalysts

**DOI:** 10.1038/s41467-021-21017-6

**Published:** 2021-02-08

**Authors:** Zhi-Peng Wu, Dominic T. Caracciolo, Yazan Maswadeh, Jianguo Wen, Zhijie Kong, Shiyao Shan, Jorge A. Vargas, Shan Yan, Emma Hopkins, Keonwoo Park, Anju Sharma, Yang Ren, Valeri Petkov, Lichang Wang, Chuan-Jian Zhong

**Affiliations:** 1 Department of Chemistry, State University of New York at Binghamton, Binghamton, NY USA; 2 Key Laboratory of Ministry of Education for Green Chemical Technology, Tianjin University, Tianjin, China; 3 Department of Physics, Central Michigan University, Mt. Pleasant, MI USA; 4 Center for Nanoscale Materials, Argonne National Laboratory, Lemont, IL USA; 5 Advanced Photon Source, Argonne National Laboratory, Lemont, IL USA; 6 Department of Chemistry and Biochemistry and the Materials Technology Center, Southern Illinois University, Carbondale, IL USA

**Keywords:** Catalysis, Electrocatalysis, Fuel cells, Nanoparticles

## Abstract

Alloying noble metals with non-noble metals enables high activity while reducing the cost of electrocatalysts in fuel cells. However, under fuel cell operating conditions, state-of-the-art oxygen reduction reaction alloy catalysts either feature high atomic percentages of noble metals (>70%) with limited durability or show poor durability when lower percentages of noble metals (<50%) are used. Here, we demonstrate a highly-durable alloy catalyst derived by alloying PtPd (<50%) with 3d-transition metals (Cu, Ni or Co) in ternary compositions. The origin of the high durability is probed by in-situ/operando high-energy synchrotron X-ray diffraction coupled with pair distribution function analysis of atomic phase structures and strains, revealing an important role of realloying in the compressively-strained single-phase alloy state despite the occurrence of dealloying. The implication of the finding, a striking departure from previous perceptions of phase-segregated noble metal skin or complete dealloying of non-noble metals, is the fulfilling of the promise of alloy catalysts for mass commercialization of fuel cells.

## Abstract (web summary)

Durability of catalysts under fuel cell reaction conditions is challenging for active nanoalloy electrocatalysts derived from platinum group metals and other transition metals. Here, the authors show that realloying in certain multimetallic nanoalloys plays a major role in enabling the high durability.

## Introduction

Proton-exchange membrane fuel cells (PEMFC) represent a clean and sustainable energy conversion vector for transportation and stationary power generation^1,2^. The mass commercialization of PEMFC, however, is hindered by high cost and poor performance of platinum group metal (PGM, e.g., Pt, Pd, etc.) catalysts for oxygen reduction reaction (ORR) taking place in the fuel cell’s cathode. Alloying noble metals with non-noble metals enhances the ORR activity while reducing the cost of catalysts^3–14^. However, the high atomic percentage of PGMs for the state-of-the-art alloy catalysts and their poor activity and durability under fuel cell operating conditions constitute major barriers to fuel cell mass commercialization^15^. Noble metal phase-segregation, e.g., “Pt-skin”^3,4,8,16,17^, by deliberate or unavoidable dealloying of the non-noble metals or deposition of the noble metals^18^, is supported by high-resolution imaging and mapping of highly-limited and carefully-selected individual particles and theoretical considerations^4,5,8,9^. Such catalysts, and intermetallic alloy catalysts as well^4^, are perhaps the predominant pathway for making most of the state-of-the-art alloy catalysts. Electrochemical dealloying approach to catalyst preparation, which dates back more than a decade ago without breakthroughs in commercializing alloy catalysts in terms of cost and stability, has shown limited success for operation in fuel cells^2,19,20^. Many of the reported ultrahigh-activity alloy catalysts contain high atomic percentages of PGM (e.g., 75 at% Pt) or toxic elements (e.g., Pb) in the as-synthesized state, feature large particle sizes (10–20 nm), or do not survive long-term durability test, and almost all perform poorly in real fuel cells^15^. For example, the rotating disk electrode (RDE) measurement of PtNi/C nanoframe catalyst was shown to display ultrahigh mass activity (MA) (5.7 A/mg_Pt_), which, however, yielded a very low activity (0.76 A/mg_Pt_) in a fuel cell^21^. In most prior studies, the origin of the poor performances of the state-of-the-art catalysts in real fuel cells is either unknown or has grossly been attributed to severe dealloying of the catalysts under the operating conditions, and no effective solutions have been found. A missing piece of information is the overall structure for the trillions of nanoparticles (NPs) in a typical catalyst layer which could range from amorphous to crystalline nature under the reaction conditions.

Here we show that realloying in PtPd-based ternary alloy NP electrocatalysts under ORR or fuel cell operating condition offers an intriguing solution to the problem. The structure durability of the alloy catalysts was characterized by in-situ/operando synchrotron high-energy X-ray diffraction (HE-XRD) to determine the alloying and realloying phase structures. A high degree of compressive strain >4% is shown to be maintained after extensive potential cycles, which is responsible for the enhanced catalytic performance in comparison with almost all prior studies of alloy electrocatalysts prepared by “on-purpose” dealloying or core–shell structuring (Supplementary Table S1). In contrast to the state-of-the-art PGM-based catalysts featuring noble metal content of about 75 at%, this dealloying–realloying process is shown to enable not only high activity and stability but also lower loading of total noble metals about 50 at% under electrochemical cell or fuel cell operating conditions. A “thermodynamically-stable alloy” has a parting limit, which is defined as a critical percentage of the more reactive metal component removable by dealloying^22^. Because of the parting limit, alloyed core and surface structures could persist under dealloying condition, and the occurrence of realloying could lead to a stainless-like alloy structure as a passivation layer of the NP with a stabilized composition. This prevents the more reactive metal component from further fast dissolution. For certain metal components alloyed in NPs smaller than a critical size, realloying of the remaining metals is a process thermodynamically favored due to a negative enthalpy^23^, as supported by recent potential-cycling experiments^24^. Alloying and realloying in the NPs could be significantly different from the bulk counterparts due to changes in the atomic mobility and the thermodynamics at the nanoscale (see Experimental Details in the Supplementary Information), as exemplified by the dramatic decrease of melting point and orders of magnitude increase of the atomic diffusion rate^25^. The atomic mobility could also be influenced by electrochemical potential. The enhanced atomic diffusion constitutes a driving force for realloying upon dealloying, as demonstrated by the penetration of alloying to a few atomic-layers of catalysts derived from pristine core–shell nanostructures after extensive electrochemical potential cycles^17^. By considering nanoscale thermodynamics, parting limit, and lattice strain deviations from Vegards’ Law (VL), a fundamental question is how a low-noble-metal-content (<50 at%) catalyst with high percentages of dealloyable metals can remain at an active alloy state under fuel cell operating conditions^26–28^ (Supplementary Fig. S1a). The formation of “thermodynamically-stable alloy” and the structure evolution of catalyst under electrochemical cell or fuel cell operating conditions were examined by several techniques or methods, including ex-situ and in-situ/operando HE-XRD coupled to pair distribution function (PDF) analysis and reverse Monte Carlo (RMC) simulations, high-resolution high-angle annular dark-field scanning transmission electron microscopy (HAADF–STEM) and elemental mapping, and density functional theory (DFT) calculations. The discovery that the alloy catalyst remains alloyed under the fuel cell operating condition is in sharp contrast to the fully-dealloyed or phase-segregated “Pt-skin” or “Pt-shell” catalysts perceived in almost all current literature reports. The significance in understanding of the thermodynamic stability of the catalyst system is a potential paradigm shift of design, preparation, and processing of alloy catalysts with high activity, low cost, and stainless-like robustness under fuel cell operating conditions.

## Results

### Alloy combination and composition

**Fig. 1:**
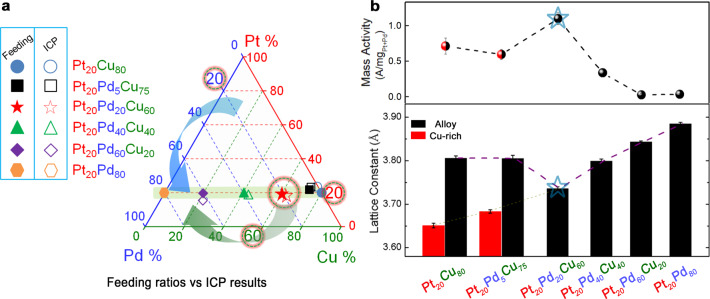
The design concept for optimizing the thermodynamic stability of ternary alloy catalysts in this work. **a** Triangle plots showing the design of Pt_20_Pd_n_Cu_80–n_ alloy NPs in terms of Pt, Pd, and Cu atomic percentages and the actual alloy NPs of selected compositions tested in this work. **b** Composition correlation of the lattice constant and the mass activity. Upper panel: the mass activities. Bottom panel: the lattice constant (determined by HE-XRD/PDFs) vs. composition for the as-prepared NP catalysts (error bars represent s.d. based on three independent experiments). The red and black columns represent the Cu-rich and Pt/Pd-rich phases, respectively.

The formation of thermodynamically-stable alloys strongly depends on the metal combinations and compositions controlled by the synthesis processes and the post-synthesis treatments, including thermochemical annealing. While extensive studies of the synthesis of binary or ternary alloy catalysts have been done^16–18^, limited success has been achieved in understanding the evolution of nanophase structures at the atomic scale, especially under the reaction conditions. It is important to note that the Pt- or Pd-based catalysts alloyed with base metals in most of the previous works including some of our own works (Supplementary Table S2) often suffered from the corrosive effect in the acidic electrolyte and hence extensive base metal dissolution. Theoretically, while it is possible to form a PGM-skin for a NP alloy (e.g., ~6.5 nm in diameter) with 75 at% PGM because there are sufficient PGM atoms to cover the particle, it is unlikely for 50 at% or less PGM content, e.g., 20 at% PGM, because the amount of PGM atoms is hardly sufficient to form a single or sub-monolayer (Supplementary Fig. S1b). For example, the amount of noble metals in a 6-nm Pt_20_Pd_20_Cu_60_ NP could only allow the formation of PGM-skin with ~2 monolayers in a phase-segregated Cu-core@PGM-shell structure. We focused on PtPd-based catalysts alloyed with base metals in a ternary alloy state, aiming at enhancing the activity and the durability in comparison with single PGM-based alloys even with the same total noble-metal content, including Pt-skin or alloy-skin nanocatalysts^29^. Pd was chosen as a noble metal Pt’s partner component because (i) catalytic synergy enabled by the dual-noble-metal character comparing with single noble metal counterparts, as demonstrated by recent studies^17,29^, (ii) Pd, like Pt, is resistant to acid corrosion, (iii) Pd is more malleable than Pt, helping self-healing^30^ upon dealloying, and (iv) Pd’s oxidation potential is lower than Pt, helping form a passivation film. Furthermore, considering the price spike of Pd due to supply–demand deficit in recent years leading to a higher price than Pt, it is also important to explore combinations of the advantages of Pt and Pd in terms of the cost per atom and the enhanced Pt-Pd catalytic synergy^17^. The dealloying–realloying process happens almost in all PGM-based fuel cell electrocatalysts during the potential cycling^17,26^. The incorporation of Pd into Pt-based nanomaterials enables a lower degree of dealloying and hence better stability. The PtPd alloyed with base metals enables fine tuning of the thermodynamic stability of the catalysts. An important emphasis is to keep the total noble metal (Pt + Pd) at a content (<50 at%) lower than the state-of-the-art PGM-based alloy catalysts which mostly contain high noble metal contents (>75 at%) and feature Pt-skin structures. With the reduction of the total noble metals (Pt and Pd) as a key emphasis of the catalyst design while harnessing the above attributes, the third metal, M′, a non-noble transition metal (e.g., Cu, Co, Ni, Fe, etc.), plays a central role in the catalytic synergy by alloying with the noble metals reflected by optimization of both electronic and strain effects, as known for simple bimetallic systems, e.g., PtCu^31^, and PdCu^32^. Another important consideration of the ternary catalyst design is the exploitation of the increased entropy effect, which is well known to homogenize the compositions towards maximum randomness or remove phase segregation for enhancing corrosion resistance^33^. By alloying^34^, a solid solution of the multimetallic components increases the entropy, leading to a higher degree of resistance to oxidation in comparison with conventional binary alloys. The ternary random alloys (e.g., Pt_20_Pd_20_Cu_60_) exhibit entropies higher than those of their binary counterparts (e.g., PtCu, PtNi, PtCo, and PtPd) or alloys with ordered phase structures. As such, a selected set of ternary alloy NPs with tunable alloy combinations and compositions (Supplementary Fig. 1c) mostly containing <50 at% Pt + Pd (Supplementary Table S2) were synthesized by a wet-chemical method. For a typical set of examples, ternary Pt_20_Pd_*n*_Cu_80−*n*_ alloy NPs were synthesized in terms of the desired Pt, Pd, and Cu atomic percentages (Fig. 1a). The compositions were determined by inductive coupled plasma-optical emission spectroscopy (ICP-OES) (Fig. 1a, and Supplementary Fig. S1d). Results showed a high tunability of the atomic compositions in NPs by manipulating the feeding ratios.

### Morphology and phase structure

The NPs feature relatively monodispersed size distributions (Supplementary Fig. S2). The carbon-supported NPs were then treated under oxidizing and reducing atmosphere. This thermochemical treatment is crucial for the structural optimization of the lattice strains, in addition to effectively removing the organic molecules capped on the as-synthesized NPs. The metal atoms in the as-prepared NPs are relatively loosely packed with an expanded lattice constant, as confirmed by the delineation of lattice constant and composition in terms of VL. The oxidative thermal treatment under an optimized O_2_ concentration yields a lattice expansion on the NPs along with surface/subsurface oxygenated base metal layers and vacancies occupied by inserted oxygen, whereas the subsequent annealing under H_2_ removes the oxygenation and enables lattice shrinking. The lattice constant of such compressed NPs closely agrees with the Vegard’s law and exhibits a stable alloy structure^35^ (Supplementary Fig. S3a). Pt_20_Pd_n_Cu_80–n_ NPs (*n* = 0–80) show face-centered cubic (fcc)-type structures as evidenced by X-ray diffraction (XRD) (Supplementary Fig. 3b). Cu doping in PtPd reduces the lattice constant effectively, as shown by HE-XRD coupled with PDF analysis (Fig. 1b and Supplementary Fig. S3c). Single fcc phase was observed for Pt_20_Pd_n_Cu_80–n_ (*n* = 20, 40, 60, 80) nanoalloys, exhibiting an apparent reduction of lattice constant in the alloy states (e.g., 3.736 Å for Pt_20_Pd_20_Cu_60_/C).

Given the understanding of the increased thermodynamic stabilities for the formation of surface amorphous oxide layers on metals under oxidative condition^36,37^ and the reduction of the metal oxide layers using hydrogen as a reducing agent^38^, as well as the reversible surface oxygenation/de-oxygenation of alloy NPs under the oxidative/reductive conditions^35^, we hypothesized that the oxidative and reductive treatments of the as-synthesized PtPdM′ (PGM < 50 at%) and tunable non-noble metal M′ composition enables a thermodynamically-stable state in terms of alloying, realloying, and lattice strains (Supplementary Fig. S3a). The realloying process not only homogenizes the inhomogeneous composition by inter-diffusion upon calcination of the as-prepared NPs, but also provides an effective pathway for self-healing upon dealloying.

With the optimal combination of alloying elements and the optimal compressive strain, the random alloy without any phase segregation would be the catalyst by design for enhancing the stability while keeping reasonable high activity^16^. The ternary catalyst, e.g., Pt_20_Pd_20_Cu_60_ (Fig. 1b), poises itself for meeting this design concept. This is supported by the strong correlation between the lattice constants (bottom panel of Fig. 1b) and the mass activities toward ORR (upper panel in Fig. 1b, details of which will be discussed later) for catalysts in different compositions, revealing a maximized compressive strain and a maximized MA for Pt_20_Pd_20_Cu_60_ catalyst. In addition to testing the maximization of activity of the ternary Pt_20_Pd_20_Cu_60_ alloy catalyst, we demonstrate for the first time dynamic realloying as the pathway towards self-healable durability under the half-cell and the fuel cell operating conditions. This is in contrast to the fact that almost all previous alloy catalysts identified from half-cell studies have been found to perform poorly in fuel cells because of the difficulty or inability to re-engineer the catalyst’s morphologies (e.g., nanoframe or nanowire) in the membrane electrode assembly (MEA) and the lack of thermodynamic stabilization^15^.

### Electrocatalytic activity and stability

**Fig. 2:**
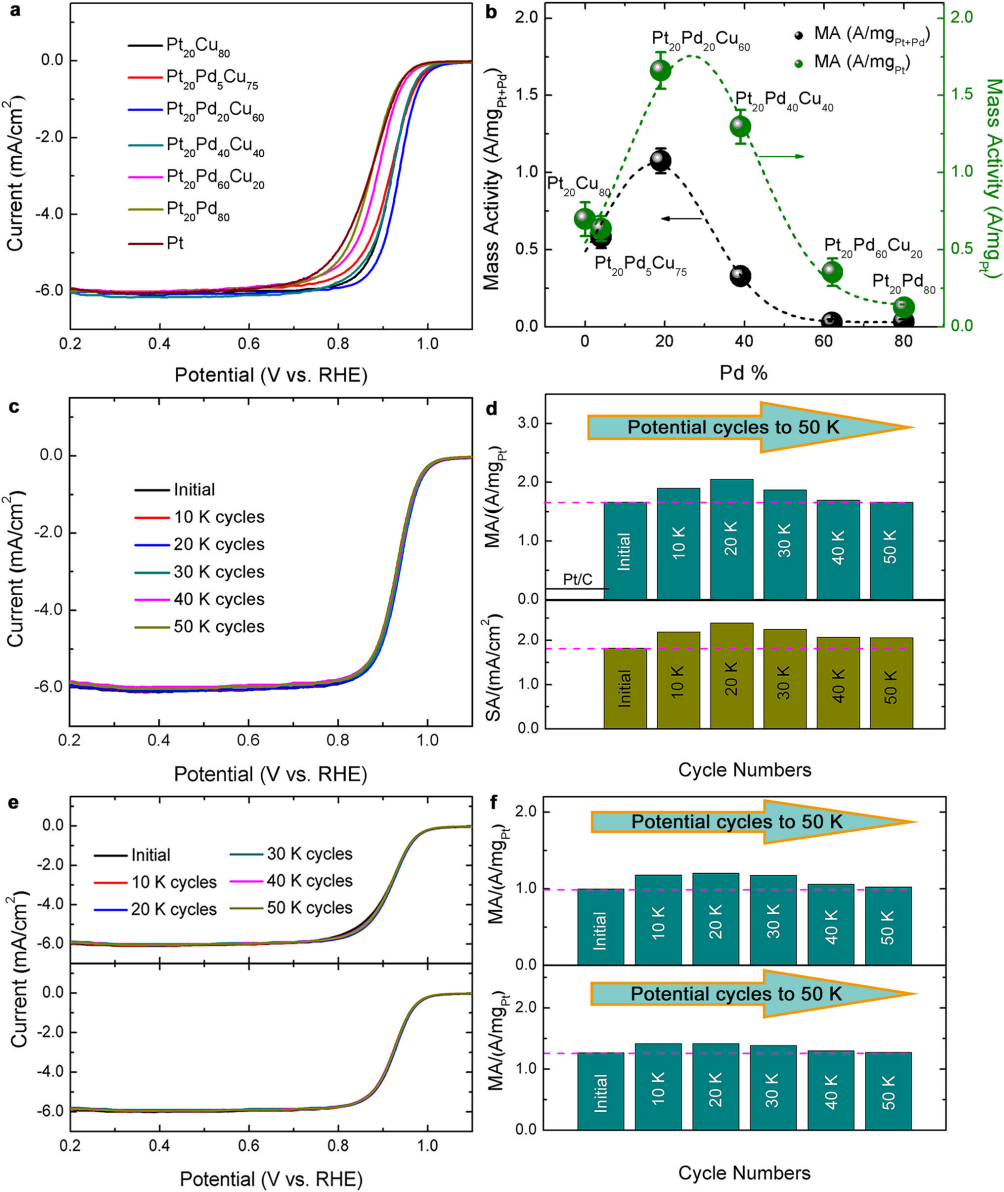
Activity and durability of the ternary alloy electrocatalysts (PtPdM (M = Cu, Ni, Co)) for ORR. **a** RDE curves for Pt_20_Pd_n_Cu_80–n_/C and the commercial Pt/C catalysts with different compositions at 25 °C in O_2_-saturated 0.1 M HClO_4_ at a scan rate of 10 mV/s and 1600 RPM (revolutions per minute). **b** The corresponding MA extracted from **a** at 0.900 V (vs. RHE) as a function of Pd% in NPs normalized by both PGM (Pt + Pd) and Pt (error bars represent s.d. based on three independent experiments). **c** RDE polarization curves of Pt_20_Pd_20_Cu_60_/C catalyst before cycling and after 10 K, 20 K, 30 K, 40 K, and 50 K cycles, respectively. **d** The corresponding MA and SA values extracted from **c** at 0.900 V (vs. RHE) of Pt_20_Pd_20_Cu_60_/C catalyst during potential cycling. **e** RDE polarization curves of Pt_20_Pd_40_Ni_40_/C (top) and Pt_20_Pd_40_Co_40_/C (bottom) catalyst before potential cycling and after 10 K, 20 K, 30 K, 40 K, and 50 K cycles, respectively. **f** The corresponding MA values extracted from **e** at 0.900 V (vs. RHE) of Pt_20_Pd_40_Ni_40_/C (top) and Pt_20_Pd_40_Co_40_/C (bottom) catalysts during accelerated durability test.

We examined the activity and durability of the well-defined ternary alloy catalysts in a standard electrochemical cell and under in operando fuel cell operating conditions. Before electrochemical test, the catalysts were subjected to a standard cyclic voltammetric (CV) activation process. For example, with the as-prepared Pt_20_Pd_20_Cu_60_/C catalyst, the CV curves in the first ~40 cycles showed gradually-diminishing Cu-redox waves at ~0.6 V (Supplementary Fig. S4a). The total reduction charge at the initial 3 cycles translates to ~3 at% of the total Cu atoms in the catalyst on the electrode surface (Supplementary Fig. S4b). The charge for Cu oxidation appeared less than that of Cu(II) reduction in the initial cycles, reflecting the additional electrochemically-induced Cu dissolution, which was not detectable after about 10 cycles. The positive shift of the peak potential with the potential cycling (Supplementary Fig. S4c) is clearly indicative of the increased energy needed to further oxidize or dissolve Cu in the alloy NPs, which is consistent with earlier observation reported for PtCu NPs^39^ and also supported by our DFT calculations (see later “Discussion”). In the electrochemical activation process, structure evolution could occur during potential cycling^7,16^. The composition dependence of the activity of Pt_20_Pd_*n*_Cu_80−*n*_/C for ORR was determined in terms of electrochemical active surface area (ECSA), MA, and specific activity (SA). A maximal ECSA value (~60.7 m^2^/g_Pt+Pd_) was obtained for our Pt_20_Pd_20_Cu_60_/C, which is slightly lower than the theoretical ECSA based on the PtPd alloy model and is comparable to that of the state-of-the-art Pt/C with a smaller particle size (76.4 m^2^/g_Pt_) (Supplementary Fig. S4d–g). Kinetic currents extracted from RDE curves (Fig. 2a, b) showed a maximum of MA (1.66 A/mg_Pt_) for Pt_20_Pd_20_Cu_60_/C, which is a ninefold increase over the commercial Pt/C catalyst (0.18 A/mg_Pt_)^40^. By normalizing the MA in terms of the total loading of Pt and Pd, it exhibits a peak MA value of 1.08 A/mg_Pt+Pd_ for Pt_20_Pd_20_Cu_60_/C, which is six times that of the commercial Pt/C catalyst (Fig. 2b). For Pt_20_Pd_40_Cu_40_/C, the MA is slightly lower (1.30 A/mg_Pt_). The catalysts with higher and lower Cu% or no Cu showed very low MA whereas the catalysts with an increased atomic Pd% showed a decrease in SA (Supplementary Fig. S4h). By accelerated durability test (ADT), Pt_20_Pd_20_Cu_60_/C appeared highly durable, as evidenced by the very small change of the ECSA values (e.g., about 90% remaining after 50,000 cycles) (Supplementary Fig. S4i, j). The MA increased by 24% after the initial 20,000 cycles, and 99.8% of MA remained after 50,000 cycles (Fig. 2c, d). The small but steady decrease in ECSA upon cycling (Supplementary Fig. S4j) can be mainly attributed to realloying, which may not be associated with Cu leaching. The realloying is a slow process, causing changes of the surface sites, including the decreased hydrogen spill over as a result of the lattice strain^41^. The fact that the MA and SA increase in the first 20,000 cycles serves as a clear demonstration that activation of the catalysts originates from the dynamic dealloying–realloying process. This type of activation was also observed in a recent study of a different alloy catalyst^17^. The subtle decreases of MA and SA after 20,000 cycles likely reflects a combination of the slow realloying process, the electrochemically-induced NP agglomeration and surface poisoning (Fig. 2d, and Supplementary Fig. S4k, l), which would require further studies.

In contrast to the results for the binary counterparts and the state-of-the-art commercial Pt/C (Supplementary Fig. S5), as well as those reported for PtPd and PtPd-based electrocatalysts (Supplementary Table S3), and other Pt-based ternary alloy electrocatalysts for ORR (Supplementary Table S4), the enhanced activity and ultrahigh durability for the PtPdCu ternary catalysts are indicative of a promising pathway for fuel cell applications. Note the potential cycling was stopped when the activity showed a significant decay, which was the case for the other Pt-based electrocatalysts which did not contain Pd and the composition changed significantly during the first 10,000 cycles. For Pd-based electrocatalysts, the MA showed a drop by 68% only after 1000 cycles^32^. The high stability of ternary catalysts was further substantiated by alloying Pt and Pd with other transition metals (Ni or Co) (Fig. 2e, f, and Supplementary Fig. S6). For example, Pt_20_Pd_40_Co_40_/C and Pt_20_Pd_40_Ni_40_/C showed no loss of MA after 50,000 cycles (Fig. 2e, f); Pt_20_Pd_20_Co_60_/C catalyst with a high Co content showed a high durability. However, Pt_20_Pd_20_Ni_60_/C catalyst showed a 20% decrease in MA after 20,000 cycles. The results demonstrated a high degree of tunability afforded by the chemical nature and composition of the third metal component. While durability measurement in the standard electrochemical cell using RDE at higher temperatures (e.g., 75 °C) may be a way to mimic the operating condition of a fuel cell, the performance evaluation is still different from a real fuel cell. As discussed later, we focused on using in-situ PEMFC experiment operated at 75 °C for the evaluation.

### Elemental mapping of catalysts

**Fig. 3:**
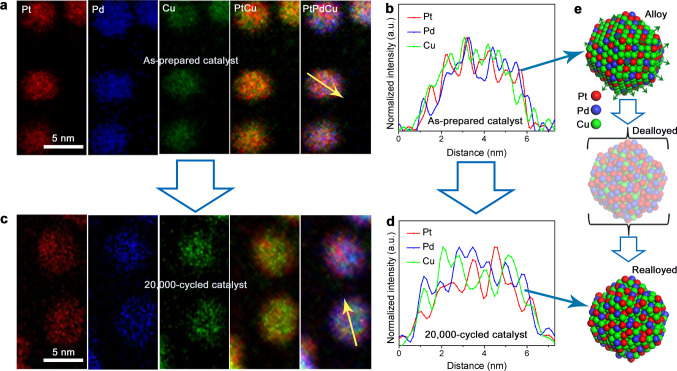
Metal component distributions in Pt_20_Pd_20_Cu_60_/C catalyst before and after potential cycling. **a**, **b** STEM-EELS elemental mapping images and corresponding EELS line-scan profiles for the as-prepared (thermochemically calcined) catalyst.** c**, **d** STEM elemental mapping images and corresponding EELS line-scan profiles for the catalyst after 20,000 cycles (electrochemically cycled) by accelerated durability test in a standard electrochemical cell. The exact atomic compositions determined by energy-dispersive X-ray spectroscopy (EDS) are Pt_21_Pd_21_Cu_58_ and Pt_27_Pd_27_Cu_46_ for the as-prepared and the 20,000-cycled Pt_20_Pd_20_Cu_60_/C, respectively. **e** Illustration of the metal component distribution of the ternary NP before (Alloy) and after (Realloyed) potential cycling based on the results shown in **b** and **d**. The intermediate “Dealloyed” NP is also included for comparison.

We next took a close look into the origin of the high activity and durability by examining Pt_20_Pd_20_Cu_60_/C catalyst before and after ADT in a standard electrochemical cell using HAADF–STEM analysis, electron energy-loss spectroscopy (EELS) elemental mapping, and line scan profile (Fig. 3 and Supplementary Figs. S7, S8). Before ADT, the carbon-supported Pt_20_Pd_20_Cu_60_ NPs were thermochemically treated (Fig. 3a, b and Supplementary Fig. S8e–h), which featured a homogeneously alloyed structure and differed from the phase-segregated core–shell type structure (PdCu@PtPd) for the as-synthesized Pt_20_Pd_20_Cu_60_ NPs (Supplementary Figs. S7a, b and S8a–d). After ADT for 20,000 cycles, the catalyst was found to preserve the alloy structure with a slightly increased NP size and contain a slightly-lower Cu% (i.e., Pt_27_Pd_20_Cu_53_, as determined by ICP-OES), despite the dynamic dealloying and realloying (Fig. 3e). The increased entropy and the optimized strain effect contributed to the stabilized structure. Note that such a small level of leaching of the non-noble metal after acid etching or long-term ADT test of our catalyst with an initial noble metal content less than 50 at.% is unprecedented. This finding is supported not only by the results from testing a series of Pt-alloy catalysts with an increase in noble metal content in this work (Supplementary Table S2), but also by results reported previously for high-Pt-content alloys starting from a low Pt content (e.g., PtNi_3_)^5,7^. The dissolution of a relatively smaller fraction of Pd into the electrolyte during the prolonged potential cycling is similar to that known for Pt upon long duration of potential cycling in fuel cells. While it is feasible to detect the very small percentage change of dissolved metal ions in the RDE experiment, which is even smaller in MEA in the experimental time scale as discussed later, measurements by ICP-OES or in-situ ICP-mass spectroscopy^42^ could gain some information for assessing the transient behavior. Nevertheless, dealloying occurs mostly at the initial few tens/hundreds of potential cycles as known by analyzing Cu redox waves in the potential cycling experiment (Supplementary Fig. S4a–c) as well as studies of other alloy NPs^40,43^. The slow realloying process is evidenced in our in-situ/operando HE-XRD experiment as described later. As confirmed by HAADF-STEM mapping and EELS line-scan profile analysis, the NPs remained a uniform ternary nanoalloy (Fig. 3c, d and Supplementary Fig. 7c, d). Specifically, the noble metal elements are slightly concentrated and a considerable amount of the base metal (e.g., Cu) remains on and near the surface region of the nanoalloy. There is no apparent indication of evolution of the nanoalloy into a core–shell or noble metal skin type structure (Supplementary Fig. S8). The composition determined by line scan profiles is not representative since differences could be observed along different directions of an individual particle or on different particles, which, along the shape irregularity of the NPs, could also lead to a discrepancy in composition from the ICP determined results and the elemental distribution homogeneity. The homogeneous distribution of elements in the ternary NPs is evidenced by the XRD and HE-XRD/PDF results, showing no indication of phase segregation. The formation of completely homogeneous alloy for the as-synthesized NPs is controlled by nucleation and growth rates of different metals during the synthesis. The subsequent thermochemical treatment used to calcine the NPs favors the formation of homogeneous alloy through structure optimization. The presence of incomplete homogenization, while insignificant, was also noticed when selected NPs were analyzed. In fact, XRD pattern of Pt_20_Pd_20_Cu_60_/C catalyst after 20,000 cycles (Supplementary Fig. S9a, b) revealed a small change in the lattice constant from 3.704 to 3.744 Å, due to a slight Cu leaching and Pt enrichment. This was supported by XPS analysis (Supplementary Fig. 9c–e). The emerging Pd^2+^ 3d and diminishing Cu^2+^ 2p_3/2_ peaks substantiate the structural evolution from PtPdCu alloy to a relatively PtPd-rich surface after leaching out Cu or CuO_x_ species during ADT. The XPS intensity of Cu 2p peak showed almost no change (Supplementary Fig. 9e), suggesting that Cu component remains abundantly near the NP’s surface region after the potential cycling. The detection of surface oxygen species for the 20,000-cycled Pt_20_Pd_20_Cu_60_/C catalyst can be attributed to the oxophilicity of the metals upon air exposure of the catalyst samples before XPS analysis. It is the existence of Cu element in the surface region after extensive electrochemical potential cycles that create surface-oxygenated metal species, as indicated by the higher oxidation states for Pt, Pd, and Cu. Further support is provided by XPS depth profile analysis using Ar ion sputtering (Supplementary Fig. S9f) and ICP-OES analysis of catalysts with different compositions after 20,000 cycles (Supplementary Fig. S9g).

### In-situ/operando determination of dynamic phase structures

**Fig. 4:**
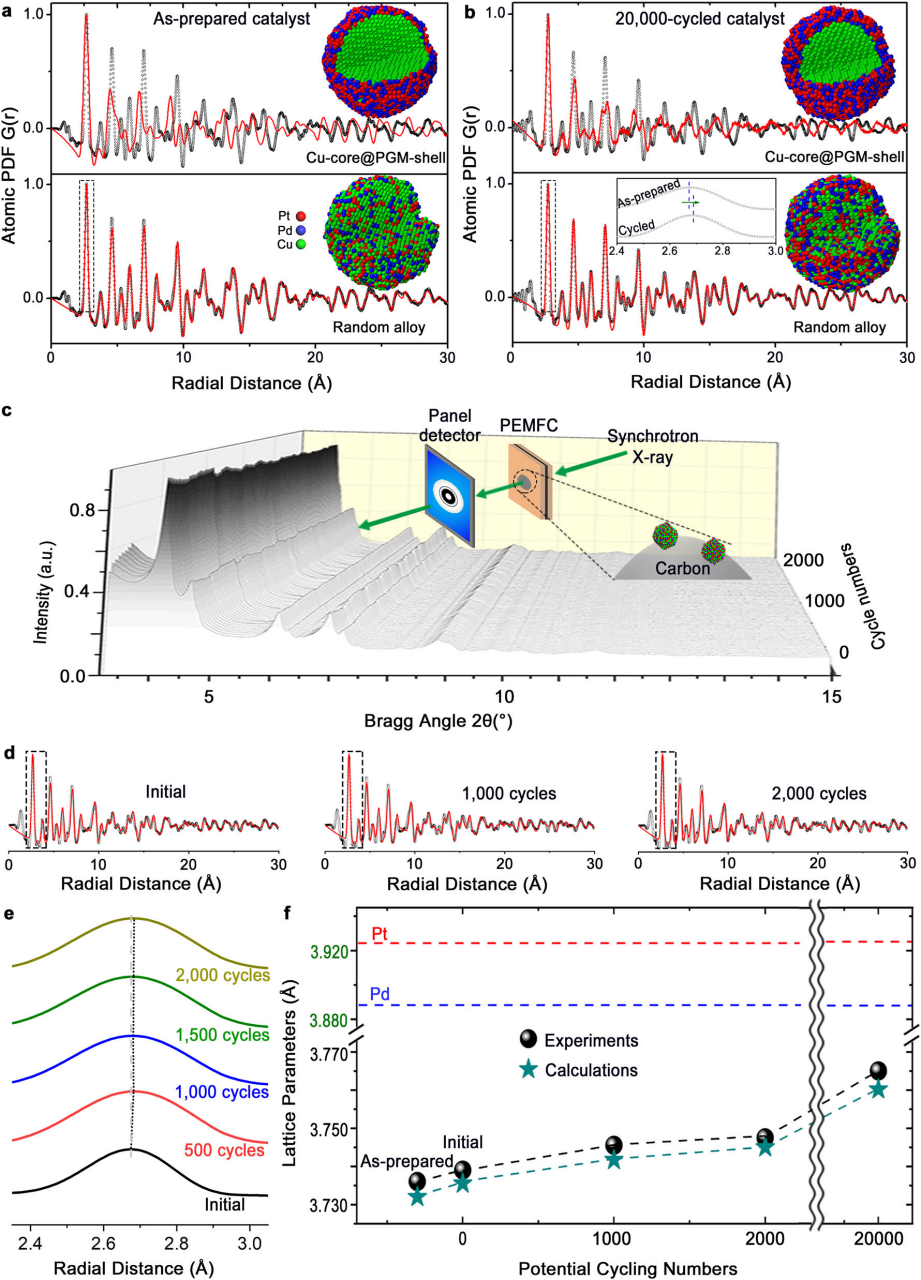
The demonstration that the alloy catalyst remained in realloyed state during and after accelerated durability test (ADT) in standard electrochemical cell and in PEMFC (in-situ/operando). Experimental (black symbols) and RMC computed (red lines) atomic PDFs as well as the corresponding 3D RMC simulation models for **a** the as-prepared, and **b** the 20,000-cycled Pt_20_Pd_20_Cu_60_/C catalyst. Upper and bottom panels show RMC models and atomic PDFs for Cu-core@PGM-shell and random alloy structures, respectively. Inset in **b**) is a magnified view of the data in the dash box for the experimentally-obtained first atomic PDF peak of as-prepared and cycled catalysts. **c** In operando HE-XRD patterns for Pt_20_Pd_20_Cu_60_/C catalyst inside an operating PEMFC upon potential cycles. **d** Experimental (black symbols) and model-computed (red lines) atomic PDFs for Pt_20_Pd_20_Cu_60_/C catalyst inside the PEMFC at 0, 1000, and 2000 cycles. **e** A close comparison of the experiment-obtained atomic PDFs at the low-r region (corresponding to the boxed regions in **d**). **f** Plots of lattice parameters obtained from the experimental and the calculated PDFs as a function of the potential cycle numbers.

Synchrotron X-ray with a high energy (105.69 keV in this work) enables a deep penetration of the materials even for small NPs which typically do not show very good crystallinities using regular XRD. Coupling synchrotron HE-XRD with PDF analysis, and RMC simulation^44^ as well, an insight can be gained into the structural evolution of catalyst under electrochemical or fuel cell operating conditions regardless of amorphous or crystalline nature of the catalyst (Supplementary Fig. S10). While X-ray absorption fine structure technique could provide local coordination as complementary information, as we observed in our previous studies of other alloy catalysts^45^, it does not provide the same information on both local and extended coordination as HE-XRD/PDF/RMC techniques as used in this work. The above findings are further substantiated by HE-XRD/PDF analysis and 3-dimentional (3D) modeling of the as-prepared and 20,000-cycled Pt_20_Pd_*n*_Cu_80–*n*_/C catalysts (Fig. 4a, b and Supplementary Fig. S11). Based on PDF analysis and RMC simulation using models of varying structures, e.g., PGM sandwich, PGM-core@Cu-shell, Cu-core@PGM-shell, Janus, and random alloy structures, etc. (Fig. 4a, b and Supplementary Fig. S11d, e), the best-fitting results revealed a random ternary alloy structure (bottom panels in Fig. 4a, b). This is clearly supported by the poor fitting results using models with PGM-skin protected Cu-core@PGM-shell structures (upper panels in Fig. 4a, b) for the same catalyst. This finding is also supported by considering the shell thickness-composition-particle size correlation (Supplementary Fig. S1b) in terms of the total PGM content (~50 at%) and NP size (~6 nm) in the formation of Cu-core@PGM-shell structure in the phase-segregated state. The absence of such core–shell phase segregation by our HE-XRD/PDF/RMC techniques rules out this possibility of the Cu-core@PGM-shell models (upper panels in Fig. 4a, b). This finding is also consistent with the thermodynamic instability of the core–shell structure given the strong atomic interdiffusion at the nanoscale. A Pt-skin structure would require at least a thickness of 1–3 MLs to maximize the activity as determined by RDE studies^16^, or a sufficiently thicker Pt-shell (>4 MLs) to protect the base metals from dissolution in the MEA^46^. In contrast to the problem with the limited layers of protective PGM-shell which are insufficient to stabilize the NPs, realloying provides an effective pathway to stability under long-term and harsh electrochemical potential cycling or fuel cell operating conditions.

The modeling results clearly reveal a single fcc phase with a slightly increased lattice constant (from 3.736 to 3.765 Å) after 20,000 cycles and a well-matched and slightly positive shift of the atomic PDF peak by about 0.02 Å (inset in Fig. 4b). We note that the lattice constant of the catalyst after such a long-term potential cycling showed the most significant compressive strain (4.1%) among the Pt-based alloy catalysts for ORR reported so far to the best of our knowledge. While Pt_20_Cu_80_/C and Pt_20_Pd_5_Cu_75_/C evolved from a phase-segregated state to a single fcc phase due to the high Cu%, which was likely above the parting limit, most catalysts remained an fcc phase. Pt_20_Pd_20_Cu_60_/C showed the smallest lattice constant among all catalysts after 20,000 potential cycles (Supplementary Fig. S11c). The finding is very consistent with the structural parameters of these catalysts before the potential cycling (Fig. 1b), indicative of a high degree of their structural stability. By RMC simulation (see Methods section for details), a bulk composition of Pt_30_Pd_22_Cu_48_ and a surface composition of Pt_35_Pd_23_Cu_42_ for the outermost ML were obtained, demonstrating a uniform distribution of the three metal elements across the NP and the existence of surface Cu in the 20,000-cycled Pt_20_Pd_20_Cu_60_/C catalyst. It is evident that the surface composition determined by RMC simulation coincides with the result for the sample after Ar-sputtering for 2 min (~Pt_40_Pd_20_Cu_40_). This composition change of Cu would translate to a quantity of about two equivalent outermost atomic layers. However, no phase segregation of PGM-skin was detected by the XRD and HEXRD/PDF/RMC results. This finding essentially rules out the possibility of PGM-skin formation since the expected phase segregation^47^ is not detected. It is therefore the diffusion of Cu atoms from the inner layers to the outermost two atomic layers where realloying occurs. An estimation of the realloying shell thickness based on the composition change yields about 4–5 atomic layers, which agrees quite well with the effective alloying shell thickness for a different ternary alloy NP system in a recent high resolution STEM study^17^.

This finding is substantiated by in-situ synchrotron XRD characterization of the catalyst on a custom-designed PEMFC under accelerated durability testing involving continuous potential cycling (Fig. 4c and Supplementary Fig. S12a). By comparing in-situ data (Fig. 4d, e and Supplementary Fig. S12b), there appears no apparent change within the measurement time frame (Fig. 4e). However, a close examination of the first PDF peak (Supplementary Fig. S12c) reveals that the peak’s radial distance) and the calculated lattice constant display a regular/irregular oscillatory kinetics as a function of the number of potential cycling. This phenomenon is believed to reflect the dealloying–realloying induced subtle structural changes, i.e., lattice constant expands upon dealloying and shrinks upon realloying, a finding consistent with findings for other nanoalloys^27,43^. It is the dealloying–realloying process that likely plays a crucial role in enhancing both activity and stability of the nanoalloy catalysts. Consistently, the lattice constants calculated experimentally via analyzing PDF data are slightly higher than those from theoretical calculation via VL, which both show similar trends with a very small increase during potential cycling and remains much smaller than those for pure Pt and Pd (Fig. 4f). This structurally stable alloy catalyst is in sharp contrast to those catalysts which undergo significant changes, e.g., recent in-situ study of PtFe nanowire catalysts under fuel cell operation conditions^40^, which showed a rapid leaching of a substantiate amount of Fe during the initial few potential cycles. A close examination reveals first-order reaction kinetics with an apparent rate constant of ~3 × 10^–5^ s^–1^ and a final lattice parameter of 3.765 Å during the first 20,000 cycles (Supplementary Fig. S12d, e). The average oscillatory frequency (Supplementary Fig. S12c) appears to fall in the range of self-diffusion of metal atoms in the surface/subsurface layers of NPs. Considering the diffusion coefficients based on recent studies of atomic surface diffusion on Pt, Cu, and Au NPs (see Supplementary Information for details), the estimated diffusion distance (0.4–4.8 nm) in the oscillatory peak-to-valley time frame showed a close agreement with the NP size. Indeed, the initial leaching of Cu, as indicated by the activation process of the as-prepared catalyst (Supplementary Fig. S4a) and the subsequent realloying, as supported by the HE-XRD/PDF data, led to a final stable alloy state. This subsequently-realloyed state is more stable than the initial state, thus preventing it from further dealloying. In-situ EDX analysis of the compositions of the catalyst during the first 2000 cycles showed a negligible change (Supplementary Fig. S12f). While it is possible that the dissolved Cu is not re-deposited in the RDE due to fast diffusion under the concentration gradient or is re-deposited in the MEA because of the solid nature of the electrolyte creating a high concentration at the interface, it is important to emphasize that the re-deposited Cu is largely irrelevant to the realloying. It is the remaining metals in the NP that are mainly responsible for the realloying or self-healing which is the focus of our work, especially from the in-situ HE-XRD/PDF experiment. This has been evidenced by the difference in compositions after potential cycling of Pt_20_Pd_20_Cu_60_ in the electrochemical half-cell (Pt_27_Pd_20_Cu_53_) and in the MEA (Pt_23_Pd_17_Cu_61_). It is the nanoscale realloying that led to the enhanced durability of the catalyst containing large amount of base metals, or a “stainless steel like” alloy structure at the optimal metal combination and composition. This alloy structure would require an increased energy to dealloy, which constitutes the basis for the high durability of the alloy catalysts. Note that the “self-healing” here means realloying of the remaining metal components structurally so that further dealloying is effectively slowed down. The electrochemical data revealed a quite consistent trend, i.e., a sharp rise followed by a slow decay towards a stable value (Supplementary Fig. S12g, h). The dealloying process is accompanied by a dynamic realloying process towards a more stable ternary alloy state which is responsible for the high durability of the catalyst. It is evident that realloying is a highly-dynamic process, the degree of which depends on a combination of composition, parting limit, and thermodynamic factors.

While a direct comparison of the catalyst’s structural durability data with the fuel cell performance data could be complicated by non-catalytic and engineering factors such as MEA optimization and fuel cell conditioning, which requires careful separation of these factors and represents a challenging area in translating high performance of advanced catalysts to technological applications^15^, the catalyst’s durability in a standard single-cell PEMFC was examined. Using the same procedure as reported in our earlier work^48^, an MEA was prepared from the Pt_20_Pd_20_Cu_60_/C catalyst and was tested in the fuel cell. The mass activity at 0.8 V for the fuel cell, 0.86 A/mg_Pt_ (or 0.54 A/mg_PGM_ based on the total mass of all PGMs) without iR compensation, clearly outperforms that of the commercial Pt/C catalyst (0.29 A/mg_Pt_) under the same operating condition (Supplementary Fig. S13a). The fuel cell’s stability is evidenced by the observation of a stable cell voltage close to ~0.6 V for at least 160 h under a current density of 1.0 A/cm^2^ (Supplementary Fig. S13b). Enhanced performance is expected by further optimization of the fuel cell engineering and testing parameters.

### Theoretical explanations

**Fig. 5:**
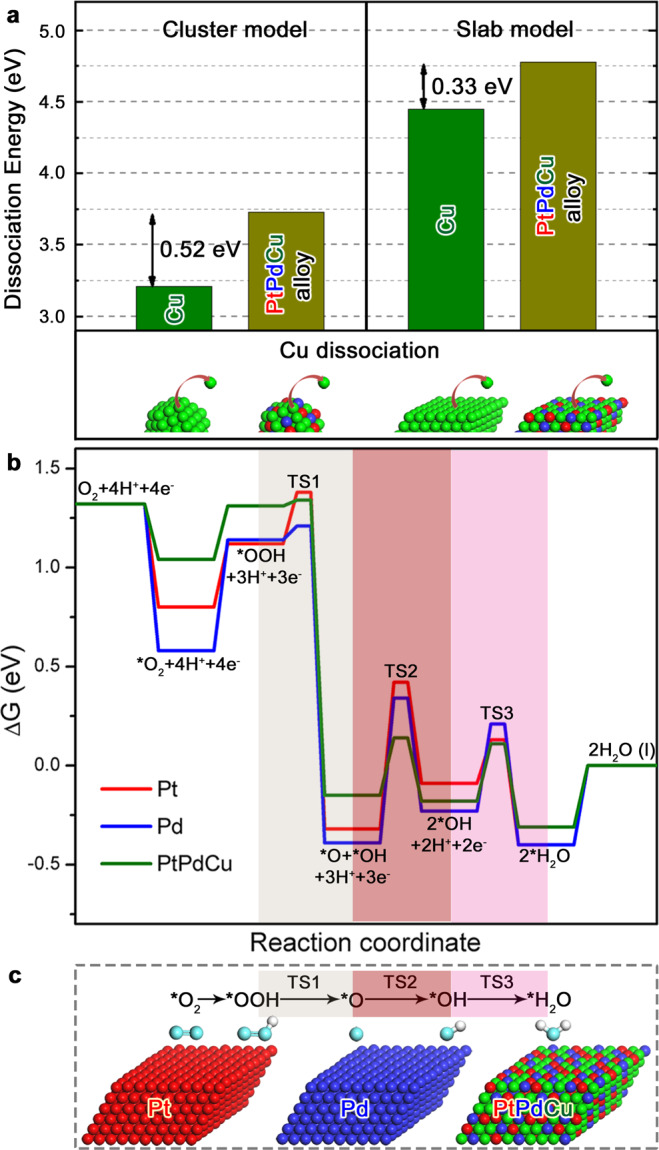
Theoretical modeling of enhanced stability and activity of the ternary alloy catalyst. **a** A comparison of the calculated dissociation energies of Cu atom from pure Cu and PtPdCu alloy based on cluster and slab models. **b** A free energy (Δ*G*) diagram comparing the energetics for the different ORR species and the reaction barriers for some key elementary steps in ORR on Pt (111), Pd (111), and PtPdCu (111) surface models at 0.9 V with respect to Computational Hydrogen Electrode (CHE). “TS1–TS3” represents the transition states for the three elementary steps, as indicated. **c** Illustration of the ORR mechanism based on the corresponding models of Pt (111), Pd (111), and PtPdCu (111) surfaces for the calculation. Red, dark-blue, green, light-blue, and white balls represent Pt, Pd, Cu, O, and H atoms, respectively.

As the enhanced ORR activity is often linked to both strain and electronic effects, DFT calculations were carried out to assess the dynamic “dealloying–realloying” process. The dissociation energy of a Cu atom from pure Cu and PtPdCu alloy was calculated using both cluster (1.5 nm) and slab surface models (Fig. 5a). At a specific composition, i.e., Pt_20_Pd_20_Cu_60_, the dissociation energy of Cu is shown to reach a maximum value (Supplementary Fig. S14a). In addition, introducing Pt into PdCu alloy models enhances the dissociation energies of Cu. Both cluster and slab models show that the dissociation energies of Cu from Pt_20_Pd_20_Cu_60_ alloy models are much higher than that from the pure Cu models with values ranging from 0.33 to 0.52 eV. In addition, the dissociation energy of Pt from Pt_20_Pd_20_Cu_60_ alloy slab model was also found to be 0.10–0.20 eV higher than that from a pure Pt slab depending on its coordination atmosphere, which is responsible for the high durability of the ternary alloy catalysts under the fuel cell working condition. The increased dissociative energies for the metal dissolution are responsible for the enhanced stability of the alloy. Upon a slight degree of Cu dissolution, the structure of Pt_20_Pd_20_Cu_60_ catalyst is shown to be realloyed to a stable alloy state. Here, the Cu atoms alloyed in the nanoscale alloy structure do not exhibit the same oxidation properties as in bulk counterpart but in a robust alloy state due to the nanoscale alloying and synergistic charge redistribution between PGM and base metals. This creates a barrier to the propensity of dissolution of Cu in the alloy, as evidenced by the fact that little Cu dissolution occurred after extensive potential cycles. This finding is consistent with the expectation that the combination of Pt and Pd often exhibits synergistically additive benefits to activity, selectivity, and stability. The calculated difference of the reduction potentials of Cu on pure Cu and PtPdCu alloy (165 mV) is also consistent with the experimental observation of a positive shift of the reduction potential of the latter in comparison with the former. In addition to the alloying-increased oxidation resistance of Cu, the maximization of the metal binding energy for Pt_20_Pd_20_Cu_60_ cluster in its composition dependence of the alloy clusters (Supplementary Fig. S14b) provides an insight into the origin of the highest stability of this catalyst. It is likely the combination of nanoscale parting limit and realloying propensity that has enabled the dynamic durability and self-healing capability. We further calculated the activation energies for O–O bond cleavage of OOH species and the protonation of O, OH, and O_2_ species on Pt (111), Pd (111), and PtPdCu (111) surfaces (Fig. 5b, c and Supplementary Fig. S14c)^49,50^. The lattice constant for Pt_20_Pd_20_Cu_60_ (111) surface model (3.77 Å), which is much lower than those for Pt (111) and Pd (111) surface models, is highly consistent with the experimental result. The large lattice strain (~4%), which is a significant factor contributing to the greatly enhanced ORR activity, is maintained even after 20,000 potential cycles. The activation energy for the protonation of a poisonous intermediate O species is found to be greatly reduced on PtPdCu (111) (0.29 eV) in comparison with Pt (111) (0.74 eV) and Pd (111) (0.73 eV). Similar conclusions can also be drawn by comparing the activation energies for the protonation of OH and O_2_ species and O–O bond cleavage of OOH (Fig. 5b and Supplementary Fig. S14c). The theoretical ORR overpotential on PtPdCu (111) alloy surface is 0.32 eV, which is much lower than that on Pt (111) surface (0.55 eV). In addition, by comparing the calculation results for both dissociative and associative pathways of ORR, the same conclusion can be reached (Supplementary Table S5). The cleavage of *OOH shows much smaller reaction barriers comparing with the reduction reactions of poisonous species *O and *OH. Given this finding, in the presence of H^+^, the corresponding reaction barriers would further drop or change slightly, keeping the overall overpotential unchanged. This would exclude it as a rate-determining step. The O_2_ molecule showed much weaker adsorption on the PtPdCu alloy model than noble metal models (Supplementary Table S6). The presence of Cu element modifies the electronic structures of the noble metals, which is responsible for the decrease of activation energies of elementary steps in ORR. The maximization of activity for ternary Pt_20_Pd_20_Cu_60_ is also supported by the intermittent value of d-band center in terms of alloy composition dependence (Supplementary Fig. S14d).

Insights into the electronic effect are obtained by the theoretical calculations, revealing: (i) reduced reaction barriers for some key elementary steps and the reduced overall ORR overpotential on PtPdCu alloy model comparing with Pt and Pd counterparts, which agrees with the experimentally enhanced ORR activity; and (ii) an increased dissociation energy of atoms in the alloy which is responsible for the improved durability. These findings reflected both electronic and strain effects, both of which are closely associated since a perturbation in strain would directly change the electronic configuration by changing bonding distance.

**Fig. 6:**
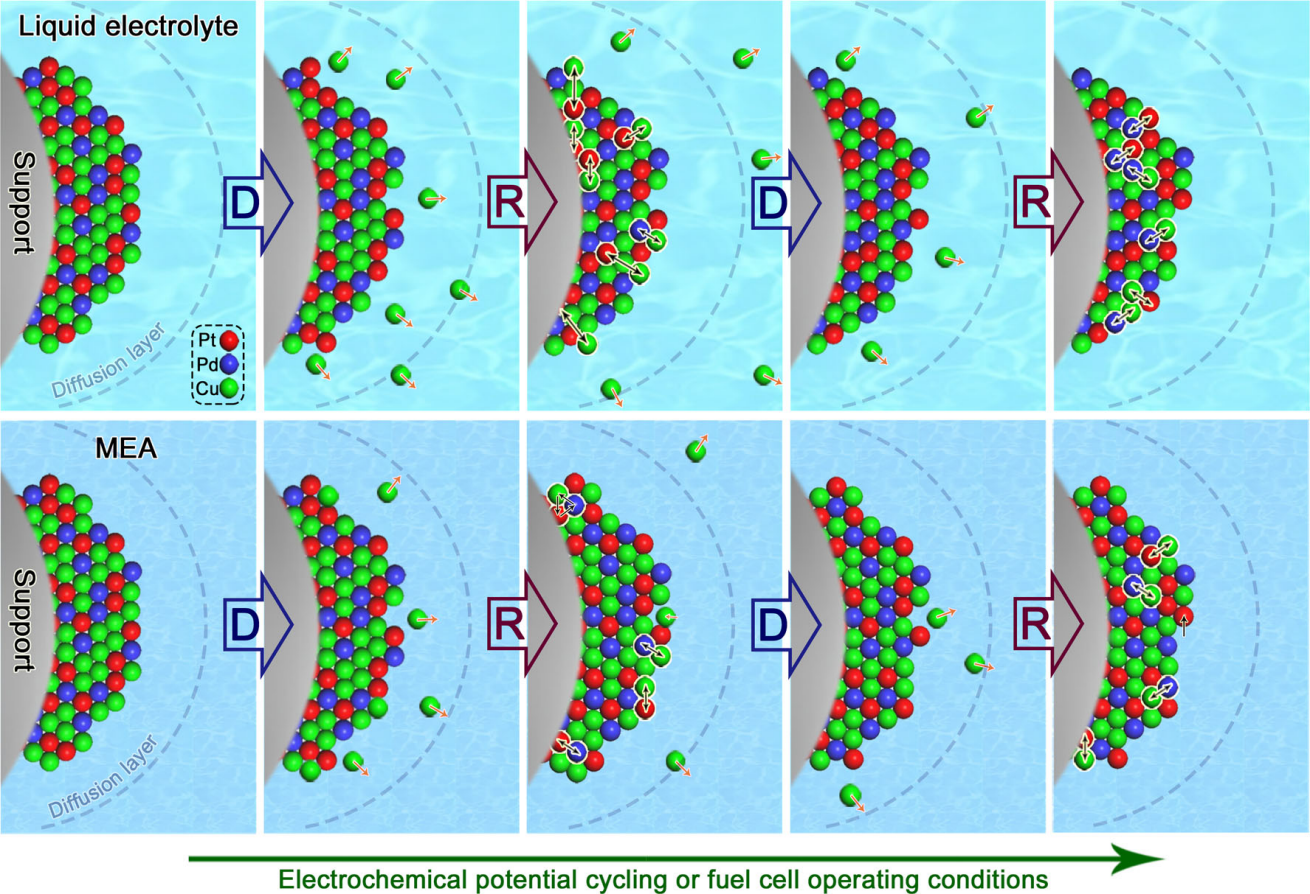
A schematic diagram illustrating the realloying process in the NP upon dealloying. Top panel: in the electrochemical liquid cell; Bottom panel: in MEA of a fuel cell. D: Dealloying; R: Realloying. The difference between the liquid cell and MEA is that the diffusion of the dissolved Cu in MEA is much slower than that in the liquid cell, resulting in much slower dealloying kinetics. Note that the various parts in the illustration are drawn not to scale.

In conclusion, the structure durability of our alloy catalysts is strongly evidenced by in-situ/operando identification of the alloyed and realloyed phases. The thermodynamically-stable Pt_20_Pd_20_Cu_60_/C catalyst exhibits not only the largest compressive strain (4.1%) after 20,000 potential cycles, but also high MA (1.66 A/mg_Pt_) and high durability (showing no activity decay after 50,000 potential cycles). As evidenced by in-situ/operando HE-XRD/PDF data in combination with HAADF-STEM/EELS and XPS analyses, as well as DFT modeling, it is the operation of the dynamic realloying or effective “self-healing” that has played a key role in the thermodynamic stability on or near the surface region of the catalyst even after more than 100,000 potential cycles. The dealloying–realloying cycles lead to an effective self-healing process of the catalyst toward a thermodynamically-stable alloy, which enhances the anti-corrosive properties for both noble and base metals (Fig. 6). Pd helps promote the realloying process and reduce the degree of dealloying. The ternary alloy composition increases the binding strength and the nanoscale entropy by several driving forces, including, i) high atomic mobility at the nanoscale (many orders of magnitude higher than bulk materials); ii) combination of the applied electrochemical potential to induce atomic rearrangement and the negative enthalpy change as known for many nanoscale alloy particles to favor realloying, and iii) parting limit for the alloying components to allow their co-existence in/on the nanoalloy. The partial dissolution of base metals, e.g., Cu, into the electrolyte during the initial potential cycles is followed by realloying process, leading to a highly effective self-healing process that realloys the remaining metals in the partially dealloyed NPs toward a thermodynamically-stable alloy. The difference between RDE and MEA is that the diffusion of the dissolved base metals in MEA is much slower than that in RDE, resulting in much slower dealloying kinetics. This insight parallels the technology on “stainless” alloys^51^, fills a knowledge gap between the structure and durability of alloy catalysts under fuel cell operating conditions while reducing Pt-content in the catalysts. Similar or subtly-different synergies are expected to operate for nanoalloy catalysts containing other noble metals such as Ir, Au, Ru, etc., depending on the actual compositions and phase structures. Our work opens a new avenue to develop efficient ternary or multimetallic catalysts combining Pt, other noble metals, and different base metals. The findings demonstrate a paradigm shift from the traditional perception of dealloying-induced phase segregation to the design and preparation of alloy catalysts that are commercially viable. Understanding the universal nature of the dynamic self-healing process will have also implications for advancing nano-engineered alloy catalysts in many other electrocatalytic reactions such as alcohol oxidation reaction fuel cells and water-splitting cells for hydrogen production, and may open new possibilities from the materials design level toward achieving low-cost, active and stable alloy electrocatalysts for fuel cells.

## Methods

### Chemicals and materials

Platinum(II) acetylacetonate (97%), palladium(II) acetylacetonate (99%), copper(II) acetylacetonate (98%), benzyl ether (99%), oleylamine (70%), oleic acid (99%), and 1,2-hexadecanediol (90%) were purchased from Aldrich. Other chemicals used, such as ethanol and hexane, were purchased from Fisher Scientific. Vulcan carbon XC-72 was purchased from Cabot. Commercial Pt/C catalyst (E-tek 20 wt% loading) was obtained from Strem Chemicals. All chemicals were used as received. Gases such as N_2_, O_2_, and H_2_ were obtained from Airgas. Millipore Milli-Q water was used.

### Synthesis and preparation of catalysts

PtPdM (M = Cu, Ni, Co) catalysts were synthesized by the wet chemical method described previously^32^. Briefly, PtPdM (M = Cu, Ni, Co NPs were synthesized by adding a total of 1 mmol with a specific feeding ratio (see Fig. 1a and Supplementary Fig. S6a) of platinum(II) acetylacetonate (Pt(acac)_2_), palladium(II) acetylacetonate (Pd(acac)_2_), and copper(II) acetylacetonate (Cu(acac)_2_), or nickel(II) acetylacetonate (Ni(acac)_2_), or cobalt(III) acetylacetonate (Co(acac)_3_) to a 125 mL benzyl ether solution. This solution was continuously stirred and purged with N_2_ throughout the addition of the reagents. A controlled amount of capping agent (5 mmol), equally divided between 2.5 mmol oleylamine and 2.5 mmol oleic acid, was added. 2 mmol 1,2-hexadecanediol was added as reducing agent. Rapid mixing of the solution ensured uniformity. The solution was then heated to 105 °C, upon which N_2_ purging was ceased. The solution was brought to a final temperature of 220–260 °C, in which the solution experienced several color changes before turning completely black, and then allowed to reflux at that temperature for 30 min. Once the solution had cooled to room temperature, the NPs were collected by adding ethanol and centrifuging for 10 min to encourage precipitation. The precipitated NPs were suspended in hexane to create an NP ink. Mixing of NP ink and Vulcan carbon (XC–72) by overnight stirring generated the catalysts. These catalysts were thermally treated: first under N_2_ to 120 °C for 10 min, and then under O_2_ (20% O_2_ balanced by N_2_) to 260 °C for 1 h, and then under H_2_ (15% H_2_ balanced by N_2_) to 400 °C for 2 h in order to remove the surfactants and anneal the catalysts, which generated the as-prepared catalysts to be used for this study. This process is scalable, as demonstrated recently in our lab for making tens of grams of catalysts in one batch.

### Characterizations

The chemical compositions of PtPdM (M = Cu, Ni, Co)/C catalysts were analyzed by inductively coupled plasma-optical emission spectroscopy (ICP-OES) using a PerkinElmer 2000 DV ICP-OES instrument. Hitachi H-7000 electron microscope and JEM-2200FS instruments were employed to analyze transmission electron microscopy (TEM) and high-resolution transmission electron microscopy, respectively. An FEI Talos F200X microscope was used to characterize the morphology of the NPs in the scanning transmission electron microscopy (STEM) mode, which is equipped with Super X energy-dispersive X-ray spectroscopy for precise and fast mapping. A Phillips X’pert PW 3040 MPD diffractometer was used to collect XRD data from 15° to 90° 2*θ* with a step size of 0.033° at room temperature. The Cu Kα radiation (*λ* = 1.5418 Å) and a sealed Xe proportional detector were used. Synchrotron HE-XRD experiments were carried out at Advanced Photon Source in Sector-11 at Argonne National Laboratory at room temperature. HE-XRD data were collected using X-rays with an energy of 105.69 keV (*λ* = 0.1173 Å). A Physical Electronics Quantum 2000 scanning ESCA microprobe was used to conduct X-ray photoelectron spectroscopy (XPS) experiments. An excitation source of focused monochromatic Al Kα X-ray (1486.7 eV), a 16-elementmultichannel detection system and a spherical section analyzer were assembled on the instrument. A 100 µm-diameter X-ray beam was rastered over a 0.2 mm by 1.4 mm rectangular spot on the sample. The binding energy was calibrated by C 1 s peak at 284.8 eV. To prepare the sample for Ar^+^ sputtering experiment, a sample of the catalyst ink (see the “Electrocatalytic activity measurement” section) was casted and dried on a small piece of molybdenum foil as sample holder, and further dried in the vacuum environment before analysis. Ar^+^ ion sputtering experiment was performed at 0.5 kV on a 2 × 2 mm^2^ sample region for a total of 6 min. XPS spectra were collected every minute. The mass metal loading of the catalysts was determined by thermogravimetric analysis (TGA) conducted on PerkinElmer Pyris 1-TGA instrument. The mass metal loading of Pt-based alloy NP electrocatalysts in this study was 20 ± 2 wt% for the standard electrochemical cell test. A higher mass metal loading of ~35 wt% of Pt_20_Pd_20_Cu_60_/C catalyst was used in the custom designed PEMFC for the in-situ HE-XRD experiments and the standard PEMFC experiments.

### Electrocatalytic activity measurement

Glassy carbon (GC) disks with a diameter of 0.5 cm were used after well-polished by 1 µm and 0.05 µm Al_2_O_3_ powders as in order. For the assembly of Pt_20_Pd_n_Cu_80–n_/C catalysts on the GC disks, 8.0 mg catalyst was suspended in 3.6 mL of deionized water with 0.28 mL of isopropanol and 0.12 mL of Nafion polymer (wt 5%), and the suspension was ultrasonicated for 30 min to generate a well-suspended ink. After sonication, 15 μL of suspension was loaded on the polished GC disk and evaporated under infrared lamp for 10 min followed by drying under room temperature for another 30 min. CV and RDE were performed by an electrochemical analyzer (CHI620a, CH Instruments) at room temperature (22–23 °C). All potential measurements from performance were given respect to the reversible hydrogen electrode (RHE) filled with electrolyte (0.1 M HClO_4_) acting as the reference electrode. A coiled Pt wire was used as the counter electrode. The catalysts were activated by electrochemical cycling between 0.05 V and 1.2 V (vs. RHE) at a scan rate of 100 mV/s in the high purity N_2_ reaerated 0.1 M HClO_4_ electrolyte for 20–30 min to ensure a stable CV curve would be obtained. CV curves were then measured between 0.02 V and 1.2 V (vs. RHE) at a scan rate of 50 mV/s. The electrochemically active surface area (ECSA) was determined by integrating the hydrogen adsorption charge on the above obtained CV curves assuming 210 μC/cm^2^ for both Pt and Pd. Afterward, the electrolyte was saturated with O_2_ to conduct RDE experiments over a potential cycling window of 0.02–1.2 V at a scan rate of 10 mV/s. The rotation speed of RDE was 1600 RPM. The iR drop was compensated for analyzing RDE results.

During the ADT, each electrocatalyst was examined in O_2_-saturated 0.1 M HClO_4_ electrolyte over a potential cycling window of 0.6–1.0 V (vs. RHE) at a scan rate of 100 mV/s for certain cycle numbers. The in-house RDE durability test mimicking the in-situ PEMFC experiments was carried out in N_2_-saturated 0.1 M HClO_4_ electrolyte over a potential cycling window of 0.6 V to 1.0 V (vs. RHE) at a scan rate of 100 mV/s for certain cycle numbers. All the RDE measurements to assess the activities of the catalysts during potential cycling follow the protocol mentioned above.

### MEA preparation for fuel cell testing and in-situ/operando HE-XRD measurement

MEAs were prepared by hot pressing method. Pure Pt/C catalyst (E-tek with 40% loading) was used as the anode hydrogen oxidation reaction (HOR) catalyst, while Pt_20_Pd_20_Cu_60_/C catalyst was applied for the cathode ORR catalysts. Generally, the catalysts were dissolved in a deionized solution containing 22% isopropanol and 3% Nafion solution (5 wt%) with the catalysts’ concentration around 2–3 mg/mL. The mixed solution was ultrasonicated for 1 h to make a well-suspended ink and then uniformly spray coated on pieces of carbon paper (Spectracarb 2050A-0550, 2.5 cm × 2.5 cm). The X-ray penetrates through the MEA, producing the catalyst information on both cathodic and anodic sides. For this reason, our design involved a much higher loading of the alloy catalyst on the cathode side (almost a tenfold Pt mass loading) than the anode side so that the collected information reflects mainly the cathode catalyst. The Pt loading on the carbon papers for pure commercial Pt/C catalyst was about 0.08 mg_Pt_/cm^2^ while for Pt_20_Pd_20_Cu_60_/C ORR catalyst was around 0.6–0.8 mg_Pt_/cm^2^. The carbon papers coated with ORR and HOR catalysts were utilized as two opposite sides of a Dupont Nafion membrane via hot pressing at 108 °C for 20 min. The ready-made sandwich-type MEAs were assembled on a custom-designed PEMFC. A modified durability testing protocol following a protocol recommended by DOE was used^52^.

For the in-situ/operando HE-XRD characterization of the MEA in a fuel cell under operating condition, a custom-designed PEMFC device and the measurement setup are shown in Supplementary Fig. S10. The PEMFC was cycled between 0.6 and 1.0 V at a scan rate of 100 mV/s for totally 2,000 cycles. During the durability potential cycling, stops at the initial point, after 50, 100, 200, 300, 500, 1000, 1500, and 2000 cycles, respectively, were carried out to conduct full range CV from 0.02 to 1.2 V at a scan rate of 50 mV/s. This test protocol has been practiced by many researchers in the literature. Other testing protocols introduced in recent years, including square wave potential cycling and cycling to higher potentials (e.g., 1.0–1.5 V), is part of our on-going work. High purity hydrogen gas (3.5% H_2_ balanced by N_2_) and high purity nitrogen gas at the well-controlled flow rate of 100 mL/min were supplied to the anode and cathode parts for PEMFC, respectively. The gases were humidified via bubbling through deionized water (78 ± 2 °C) to achieve 100% humidity. The temperature of the PEMFC was reposefully maintained around 75 ± 2 °C using a wrapped heater around the PEMFC and a thermal controller. The selected CV curves at each stop were analyzed to calculate the ECSA of the MEAs. The in-situ experiments were performed in a custom-built fuel cell which has a higher cell resistance in comparison with standard cell due to the deformed cell sealing effect posed by the presence of a hole in the middle cell electrode plate to allow synchrotron X-ray penetration and scattering out. As such, the CV curves from the in-situ cell were somewhat distorted in comparison with those from the regular fuel cell as reported in our work^53^. The ECSA values were calculated using Pt–O reduction peak. Nevertheless, the ECSA results obtained from Pt–O reduction peak by MEA and in-house RDE measurement show very similar results.

For testing the MEA performance in a standard fuel cell, a standard 5.0-cm^2^ single-cell (Electrochem Inc.) was used. MEAs for the single fuel cell performance test were prepared by a similar but slightly modified method. Briefly, the catalyst ink was brushed on carbon cloth (CeTech W151010) as the gas diffusion layer. Then hot pressing (120 °C) was applied to prepare MEAs using Pt_20_Pd_20_Cu_60_/C (0.15 mg_Pt_/cm^2^) and commercial Pt/C (0.40 mg_Pt_/cm^2^) as the cathode and anode catalysts, respectively. The single-cell test station (Electrochem Inc.) was used. The fuel cell was maintained at 75 °C for the duration of the measurement. The gases for both the anode and cathode were passed through a humidifier (Electrochem Inc, HAS-TC-GTL) at 65 °C and 100% humidification. Flow rate was held at a stoichiometric rate of H_2_ 300 mL/min at the anode and O_2_ 150 mL/min at the cathode, respectively, using MTS-A-150 (Electrochem Inc.) with a back pressure held at 30 psi for both anode and cathode. Prior to the durability test, a current density of about 2.15 A/cm^2^/mg_Pt_ (0.32 A/cm^2^) was applied to the fuel cell for about 16 h until the cell voltage reached to a stable value. Then the fuel cell was operated by holding a current density while measuring the corresponding voltage as a function of time. Standard potential cycling test was also performed for the durability study.

### Atomic PDFs derivation and interpretation

The HE-XRD experimental data was first reduced to the structure factors, *S*(*q*), and then Fourier transferred to atomic pair distribution functions (PDFs) *G*(*r*) defined as:

1 $$G\left( r \right) = 4\pi r(\rho (r)-\rho o),$$

where *ρ(r)* and *ρo* are the local and average atomic number density, respectively. *G*(*r*) oscillated about zero and shows positive peaks at real space distances (*r*) separating pairs of atoms, immediate and all farther neighbors, within the studied NPs. The area under the peaks is proportional to the number of atomic pairs at those distances. Furthermore, because surface atoms at the opposite sides of NPs are separated the most, PDF peaks at higher-r distances are very sensitive to atoms near the NP’s surface. Hence, the experimental atomic PDFs obtained here are sensitive to the atomic-level structure throughout the studied NPs, including their surface. This fact may not come as a surprise because atoms at the surface of NPs explored for catalytic applications occupy a very substantial fraction (about 20% for particles <1 nm) of their overall volume, and XRD is known to be sensitive to the volume fraction of the constituents of a metallic material down to a few %.

### 3D structure modeling and Reverse Monte Carlo (RMC) simulation

The 3D structure models shown in Fig. 4a, b and Supplementary Fig. S11d, e were built by molecular dynamic (MD) simulations. The initial models were equilibrated for 200 ps at 400 °C (the operation temperature for the thermochemical annealing under H_2_/N_2_ atmosphere) and then cooled down to room temperature at a rate of 50 °C per step and finally equilibrated for another 100 ps. RMC simulations were carried out to refine the configurations obtained from the MD simulation results. During the simulations, the energy of the model was described by paired potentials obtained from the literature and kept close to the MD-obtained result as much as possible. Models will undergo consecutive MD and RMC runs when necessary. Further simulation details are provided in the Supplementary Information.

### Computational modeling

The DMol^3^ package in the Materials Studio Program was used for Density Functional Theory (DFT) calculations. The Perdew–Burke–Ernzerhof (PBE) functional and a double-numerical basis set with polarization functions were used in all our DFT calculations. Computation using the Revised PBE functional generally yields a binding energy which is 0.2–0.3 eV weaker than that obtained using PBE. However, the activation barrier, reaction energy and relative binding energy show little change. To calculate the dissociation energy of Cu, two kinds of models, i.e., cluster models and slab surface models were used. The cluster models include pure metal and alloy phase cluster models that consist of 116 atoms in the cluster with a size around 1.5 nm. The surface models have five layers with the bottom three layers fixed. A 4 × 4 unit cell and a (3 × 3 × 1) k-point mesh were used. Between the repeated slabs along the *z* axis, a 15 Å vacuum space was applied to eliminate interaction between the slabs.

The reduction potential of Cu was calculated by:

2 $$E_{{\mathrm{SPR}}} = \left( {\frac{{\Delta {\it{\epsilon }}}}{{\nu \left| {\mathrm{e}} \right|}} - 4.43} \right),$$

where *E*_SPR_ is the standard redox potential, Δ*ε* is the energy change from bulk metal to hydrated metal ion and represents in “eV” unit, *ν* is the number of electrons being transferred to the cation. The absolute value of 4.43 eV was used for the potential of hydrogen electrode^54^. The energy change from the bulk metal to the hydrated metal ion was estimated by:

3 $$\Delta {\it{\epsilon }} = {\it{\epsilon }}_{{\mathrm{cohe}}} + {\it{\epsilon }}_{{\mathrm{ion}}} + {\it{\epsilon }}_{{\mathrm{hydra}}}( + {\it{\epsilon }}_{{\mathrm{li}} - {\mathrm{ex}}}),$$

where *ε*_cohe_ is the sublimation energy from solid to gas phase and is equivalent to the cohesive energy, *ε*_ion_ is the ionization energy, *ε*_hydra_ is the hydration energy, and *ε*_li-ex_ is the ligand exchange energy^55^. For example, in calculating the reduction potential of Cu(II)/Cu, the number of electrons transferred is two. The *ε*_cohe_ energy gap is associated with the dissociation energy gap while other terms are kept the same. When calculating the reduction potential shift of Cu from pure Cu to PtPdCu alloy, the dissociation energy gap is 0.33 eV, which translates to 165 mV for shift of the reduction potential of Cu.

DFT calculations to obtain the reaction barriers of some representative elementary steps were carried using three-layer periodic slab models with the bottom two layers fixed. The simulation details can also be found in our previous DFT work on calculating transition states^56,57^. The binding energies of the adsorbates which were fully relaxed were calculated by

4 $$\Delta E_{{\mathrm{ad}}} = \left[ {E_{\mathrm{R}} + \mathop {\sum}\nolimits_j {\frac{{hv_j}}{2}} } \right] + \left[ {E_{{\mathrm{metal}}\,{\mathrm{surface}}} + \mathop {\sum}\nolimits_j {\frac{{hv_j}}{2}} } \right] - \,\left[ {E_{{\mathrm{R}}/{\mathrm{metal}}\,{\mathrm{surface}}} + \mathop {\sum}\nolimits_j {\frac{{hv_j}}{2}} } \right],$$

where *E*_R_, *E*_metal surface_, and *E*_R/metal surface_ are the total energy of the adsorbates, such as H_2_O and O_2_ molecules, the isolated model metal surface, and the adsorbates that were adsorbed on the metal surfaces, respectively. *h* is the Planck’s constant. *ν*_*j*_ is the vibrational frequency of each binding configuration. The slab models of Pt (111), Pd (111), and Pt_20_Pd_20_Cu_60_ (111) surfaces were used. The Gibbs free reaction barriers and reaction energies of some key elementary steps, e.g., the O–O bond cleavage of OOH, the protonation reactions of O_2_, O, and OH species on different surfaces were calculated by:

5 $$\Delta G_a = \left[ {E_{{\mathrm{TS}}} + \mathop {\sum}\nolimits_j {\frac{{hv_j}}{2}} } \right]-\left[ {E_{{\mathrm{IS}}} + \mathop {\sum}\nolimits_j {\frac{{hv_j}}{2}} } \right] - T\left[ {S_{{\mathrm{TS}}} - S_{{\mathrm{IS}}}} \right],$$

6 $$\Delta G = \left[ {E_{{\mathrm{FS}}} + \mathop {\sum}\nolimits_j {\frac{{hv_j}}{2}} } \right]-\left[ {E_{{\mathrm{IS}}} + \mathop {\sum}\nolimits_j {\frac{{hv_j}}{2}} } \right] - T\left[ {S_{{\mathrm{FS}}} - S_{{\mathrm{IS}}}} \right],$$

where *E*_IS_, *E*_TS_, and *E*_FS_ denote the total energies of the reactant, the transition state, and the product, respectively, while *S*_IS_, *S*_TS_, and *S*_FS_ denote the entropies of the reactant, the transition state, and the product, respectively^56^. We note here that the zero point energy correction was considered for all calculations. The d-band centers of the noble metals on the first surface layer in the periodic models were calculated by:

7 $$\varepsilon _d = \frac{{{\int} {E\rho (E,r)dE} }}{{{\int} {\rho (E,r)dE} }}.$$

The computational hydrogen electrode as proposed by Nørskov and co-workers was used to determine the free energy diagram of ORR as a function of potential^50^. The ORR free energy diagrams at *U* = 0 V and *U* = 0.9 V were calculated, respectively.

## Supplementary information

Supplementary Information

Peer Review File

## Data Availability

The datasets generated and/or analyzed in the current study are available from the corresponding authors upon reasonable request, and are also included with the manuscript as Supplementary Information.
