# Supplementary material for: Alloying–realloying enabled high durability for Pt–Pd-3d-transition metal nanoparticle fuel cell catalysts
Source: Nat Commun. 2021 Feb 8;12:859. doi: 10.1038/s41467-021-21017-6 (PMC7870895; doi:10.1038/s41467-021-21017-6)
Supplement: Supplementary file 1 — Supplementary Information [file 41467_2021_21017_MOESM1_ESM.pdf]

Supplementary Information

Alloying–Realloying Enabled High Durability for Pt–Pd–*3d*-Transition Metal Nanoparticle Fuel Cell Catalysts

Zhi-Peng Wu,[1,2] Dominic T. Caracciolo,[1] Yazan Maswadeh,[3] Jianguo Wen,[4] Zhijie Kong,[1] Shiyao Shan,[1] Jorge A. Vargas,[3] Shan Yan,[1] Emma Hopkins,[1] Keonwoo Park,[1] Anju Sharma,[1] Yang Ren,[5] Valeri Petkov,[3,]* Lichang Wang[2,6,]* and Chuan-Jian Zhong[1,]*

[1] Department of Chemistry, State University of New York at Binghamton, Binghamton, NY 13902, USA.
[2] Key Laboratory of Ministry of Education for Green Chemical Technology, Tianjin University, Tianjin 300072, China.
[3] Department of Physics, Central Michigan University, Mt. Pleasant, MI 48859, USA.
[4] Center for Nanoscale Materials and X-ray Science Division, Argonne National Laboratory, Lemont, IL 60439, USA.
[4] Center for Nanoscale Materials, Argonne National Laboratory, Lemont, IL 60439, USA.
[5] Advanced Photon Source, Argonne National Laboratory, Lemont, IL 60439, USA.
[6] Department of Chemistry and Biochemistry and the Materials Technology Center, Southern Illinois University, Carbondale, IL 62901, USA.

*Correspondence to: cjzhong@binghamton.edu, lwang@chem.siu.edu, petkolvg@cmich.edu.

Supplementary Figures 1 – 14

Supplementary Tables 1 – 7

Additional Details on Methods and Theoretical Considerations:

Considerations of atomic mobility (diffusion) and thermodynamics at the nanoscale. The atomic diffusion, which is referred to the atom displacement induced by thermal energy, is governed by the Arrhenius-type equation:

$$D_{\infty} = D_{0,\infty} e^{-\frac{Q_{\infty}}{RT}} \quad (1)$$

where $D_{0,\infty}$ is the pre-exponential factor, Q_{∞} refers to the thermal activation energy regardless of size and shape effects, R is the ideal gas constant while T is the absolute temperature. The thermal activation energy Q_{∞} is proportional to the melting temperature $T_{m,\infty}$ via a constant coefficient C in the following equation:

$$Q_{\infty} = CT_{m,\infty} \quad (2)$$

At the nanoscale, the diffusion is accelerated by the dramatically enhanced surface area to volume ratios and quantum effect at the interatomic distance range. The melting temperatures of nanoparticles are highly dependent on the particle size, which decreases at the nanoscale in comparison with the bulk counterparts. Therefore, the thermal activation energy is proportionally decreased while the atomic diffusion rate is exponentially enhanced with the particle size at the nanoscale[1]. The nanoscale effect contributes to the greatly-decreased melting temperature, thus leading to an enhanced atomic diffusion rate in nanoparticles. The nanoscale atomic diffusion is driven by thermal energy, kT. Given the negative ΔH values for many nanoscale alloys and positive ΔS value for alloying (enthalpy change and entropy change, respectively), alloying could be favored even at moderate temperatures from the thermodynamic viewpoint ($\Delta G = \Delta H - T\Delta S$), or driven by electrochemical potential of the metal components in terms of $\Delta G = -nFE$ (where ΔG is the Gibbs free energy, n is number of electrons transferred, F is the Faraday constant, and E is the electrochemical potential).

The diffusion of atoms in/on NPs can be evaluated in terms of the mean square displacement (MSD), which is defined as[2]:

$$MSD = \Delta r^2\,(t) = \pi r^2 \quad (3)$$

where r is the diffusion radius, t is the diffusion time. MSD can be further described by[2]:

$$MSD = 6Dt \quad (4)$$

where D is the diffusion coefficient of atoms. For transition metal NPs such as Pt, Pd, and Cu NPs, the atomic diffusion coefficients fall into a range of 1×10^{-18} to 1×10^{-16} cm^2/s [3–6]. The average diffusion time during the dealloying-realloying process can be roughly estimated from the oscillatory peak-to-valley time frame (Supplementary Fig. 12c), which is about 800–1200 s. The estimated diffusion radius (or distance) of the atoms would be 0.4 ~ 4.8 nm, which agrees with the length scale in terms of the surface/subsurface layers and the NP sizes.

Reverse Monte Carlo (RMC) simulation. RMC simulation was used to refine the MD generated models. In RMC simulation, positions of atoms in the MD models were adjusted as to minimize the difference between the model-computed and experimental-obtained atomic PDFs. During the simulations, Pt and TM atoms were constrained both to maintain as maximal (i.e., as close to 12) as possible coordination numbers. The distances were determined from the positions of the first peak in the experimental atomic PDFs. Simulations were done with the help of a new version of the program RMC++[7]. More details of modelling the 3D structure of metallic NPs by joint MD and RMC computations can be found in our previous works[8].

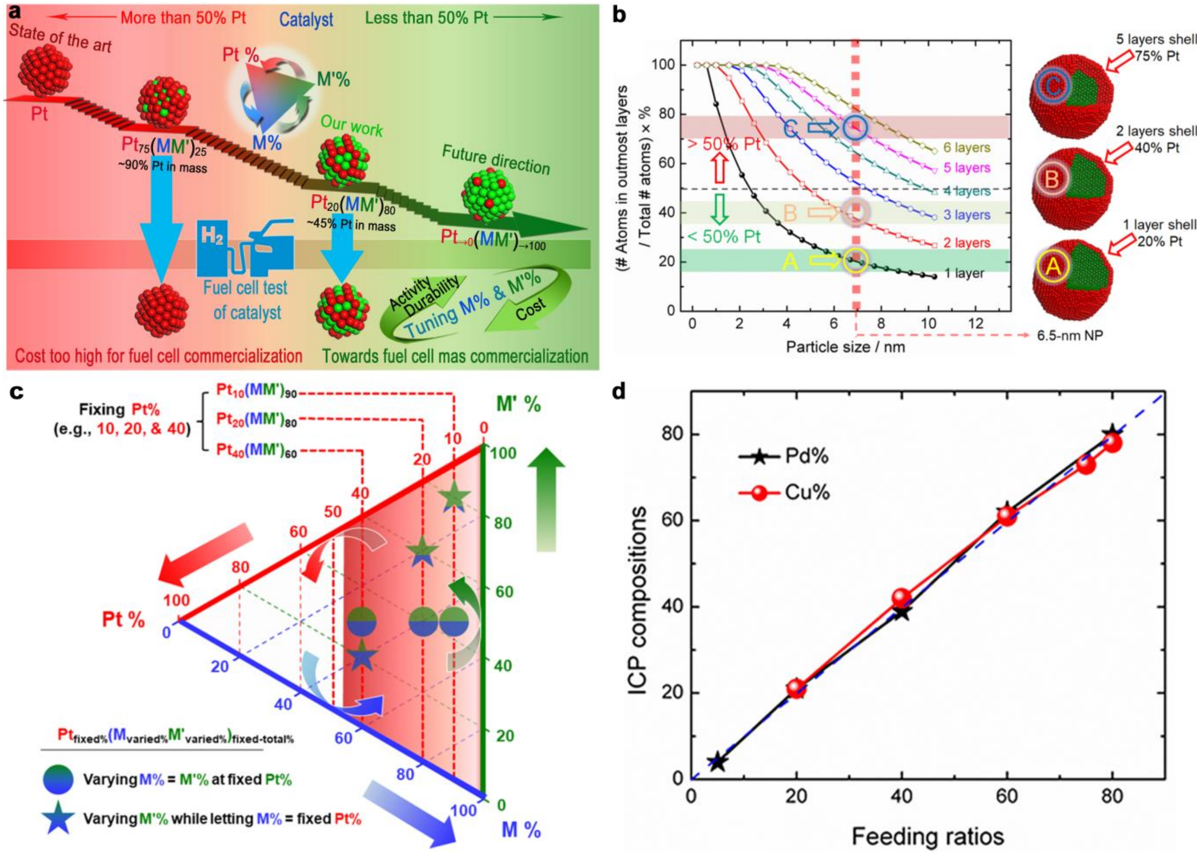

Supplementary Fig. 1 | Chart illustration of the progress of Pt catalysts development and evolution. a, Pt catalysts development towards higher activity & durability with lower cost, by tuning composition of ternary alloy ($Pt_x(MM')_y$, M, M′: other transition metals), in comparison with most of the state-of-the-art catalysts with higher Pt content (>50%), and the catalyst composition evolution in fuel cell durability test. The corresponding Pt mass ratios are also indicated in the graph for comparison. Note that M and M’ are considered as 3d-transition metals when converting atomic ratios to mass ratios for simplicity in the calculation. **b**, Plots of the percentage of the number of atoms (e.g., Pt) in the outmost layers of metal nanoparticles in terms of the different number of layers (1, 2, …, and 6) as a function of the nanoparticle diameter. **c**, An illustration of the design of Pt(MM') ternary alloy catalysts with different levels of reduction of the amount of Pt (in Pt%). The points in the Pt% < 50% region (red-highlight) show two scenarios: (I) Varying M% and M'% at fixed Pt% (e.g., Pt% fixed at 10%, 20%, and 40%). (II) Varying M'% while letting M% equal to fixed Pt%. **d**, Plots of the fraction (n) in $Pt_{20}Pd_nCu_{80-n}$ NPs of Pd and Cu determined by ICP–OES as a function of the feeding ratios.

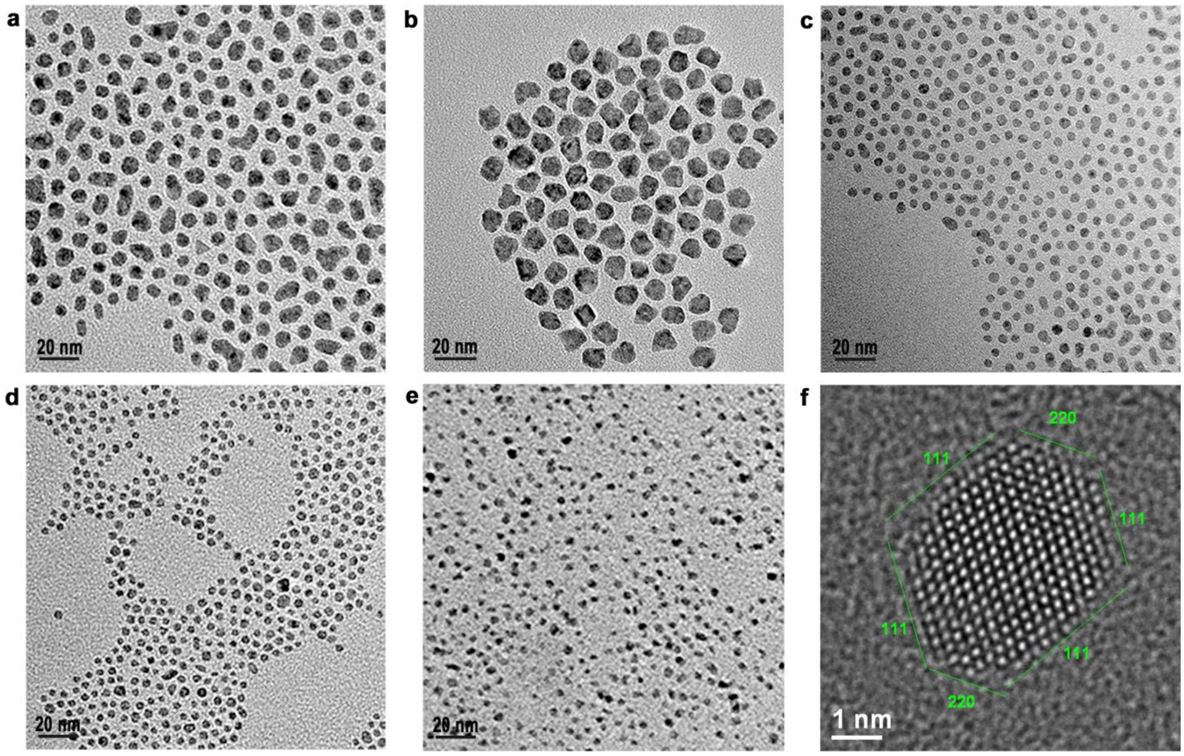

Supplementary Fig. 2 | Morphology characterizations of $Pt_{20}Pd_nCu_{80-n}$ NPs. a-e, TEM images of the as-synthesized NPs. TEM images of **a**, $Pt_{20}Cu_{80}$, **b**, $Pt_{20}Pd_5Cu_{75}$, **c**, $Pt_{20}Pd_{20}Cu_{60}$, **d**, $Pt_{20}Pd_{40}Cu_{40}$, and **e**, $Pt_{20}Pd_{60}Cu_{20}$ NPs. The corresponding size distributions are 6.6 ±0.7 nm for $Pt_{20}Cu_{80}$, 9.7 ±0.7 nm for $Pt_{20}Pd_5Cu_{75}$, 4.6 ±0.4 nm for $Pt_{20}Pd_{20}Cu_{60}$, 4.3 ±0.4 nm for $Pt_{20}Pd_{40}Cu_{40}$, and 4.1 ±0.4 nm for $Pt_{20}Pd_{60}Cu_{20}$. **f**, HR-TEM image of the as-synthesized $Pt_{20}Pd_{20}Cu_{60}$ NPs.

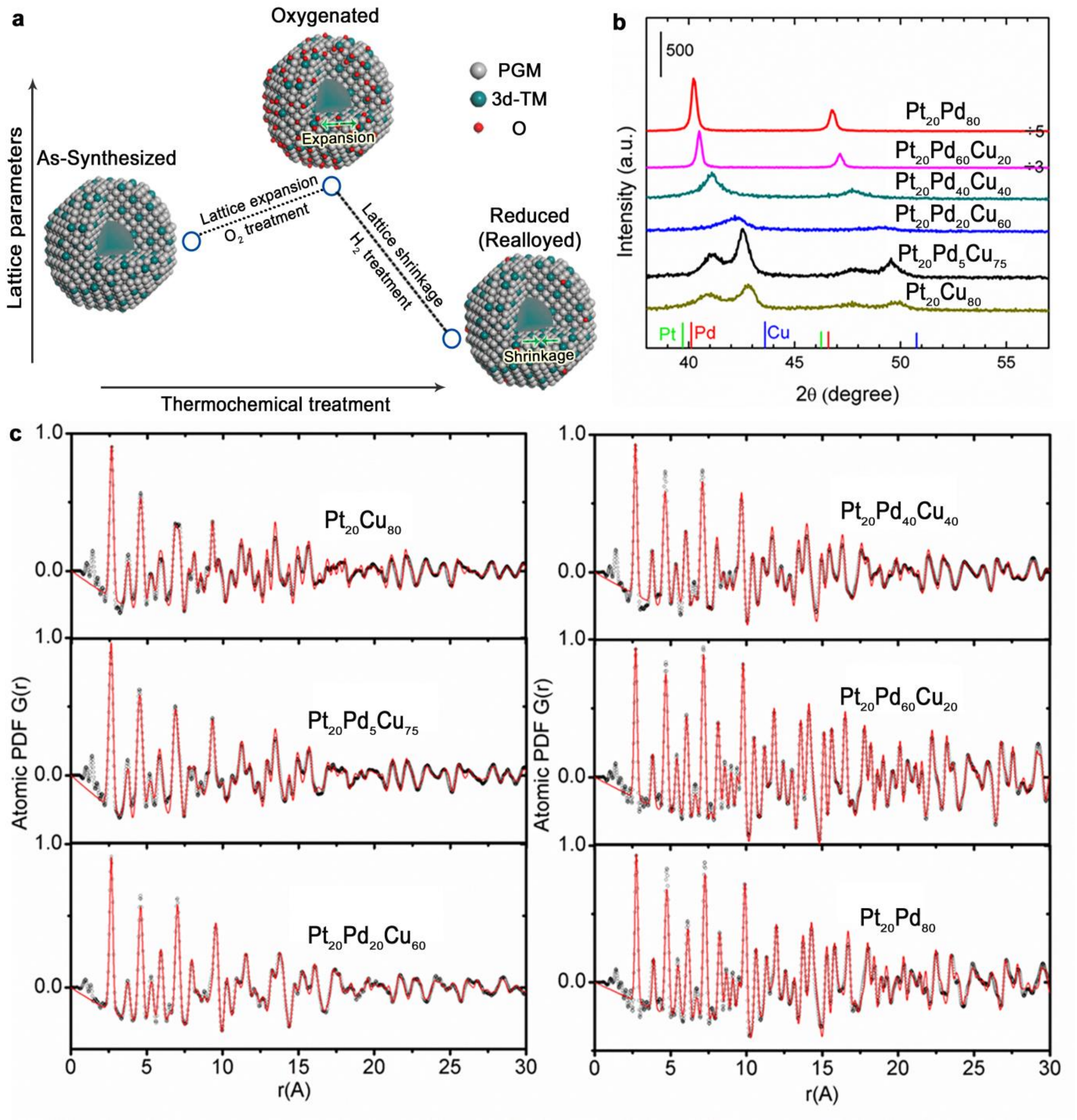

Supplementary Fig. 3 | Physical characterizations of $Pt_{20}Pd_nCu_{80-n}$/C catalysts. a, Optimization of the lattice strain by a combination of alloying and thermochemical treatment, which was demonstrated for many different alloy catalysts[9]. Gray, blue, and red atoms represent noble metal, non-noble metal, and oxygen atoms, respectively. **b**, XRD patterns of $Pt_{20}Pd_nCu_{80-n}$/C catalysts. **c**, Experimental curves (black symbols) and phase structure model-derived atomic PDFs (red lines) for the as-prepared $Pt_{20}Pd_nCu_{80-n}$/C catalysts.

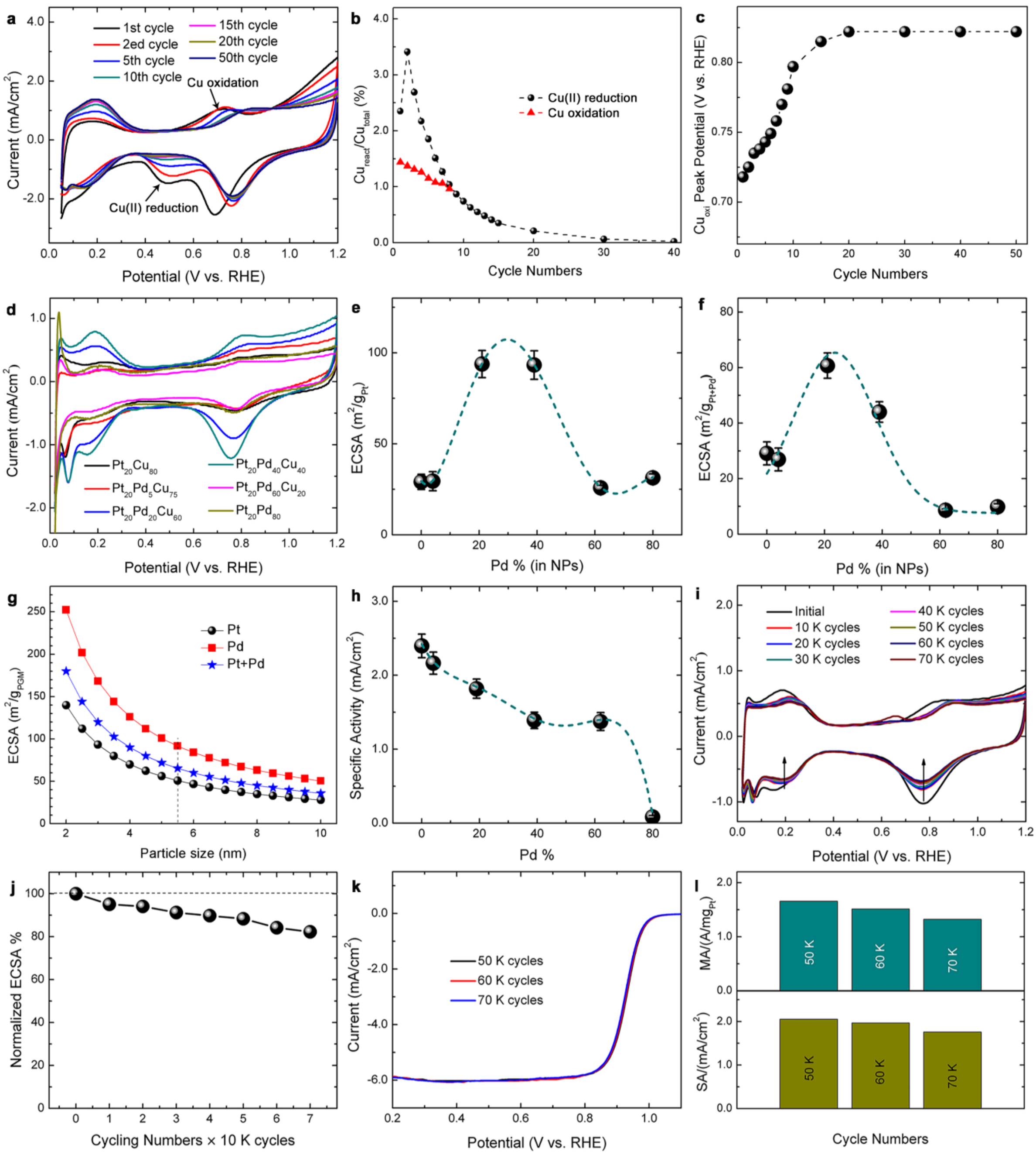

Supplementary Fig. 4 | Electrochemical properties of $Pt_{20}Pd_nCu_{80-n}$/C catalysts. **a**, CV curves of the activation process for the as-prepared $Pt_{20}Pd_{20}Cu_{60}$/C catalyst at a scan rate of 100 mV/s. **b**, The integrated charges for the copper oxidation and reduction waves at ~0.6 V in **a**, expressed as the ratio of the reacted Cu to the total Cu in the catalyst on the electrode surface as a function of the potential cycle number. **c**, The peak potential positions for the Cu oxidation wave in **a** as a function of the potential cycle number. **d**, CV curves for $Pt_{20}Pd_nCu_{80-n}$/C catalysts with different compositions. **e**, **f**, Plots of the ECSA values based on **e**, Pt and **f**, platinum group metals (Pt and Pd) as a function of Pd% in NPs. **g**, The theoretically-estimated ECSA values for Pt, Pd, and PtPd alloy spherical nanoparticles as a function of particle size. **h**, SA values as a function of Pd% in NPs. Error bars represent s.d. based on three independent experiments in e, f, h. **i**, CV curves and **j**, the corresponding ECSA values of $Pt_{20}Pd_{20}Cu_{60}$/C catalyst before cycling and after 10 K, 20 K, 30 K, 40 K, 50 K, 60 K, and 70 K cycles. **k**, RDE polarization curves of $Pt_{20}Pd_{20}Cu_{60}$/C catalyst after 50 K, 60 K, and 70 K cycles. **l**, The corresponding MA and SA values extracted from **k** at 0.900 V (vs RHE) of $Pt_{20}Pd_{20}Cu_{60}$/C catalyst during potential cycling from 50 K to 70 K cycles.

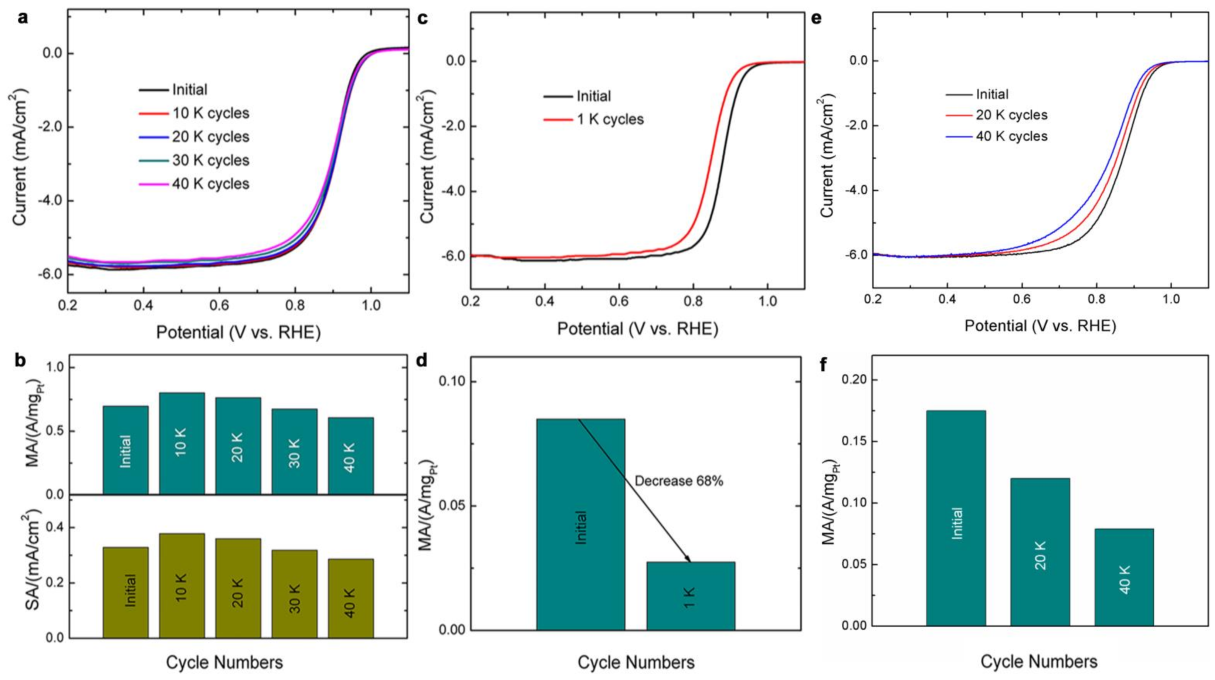

Supplementary Fig. 5 | ORR accelerated durability test of $Pt_{20}Pd_{40}Cu_{40}$/C, $Pd_{50}Cu_{50}$/C, and the commercial E-tek Pt/C catalysts. a, RDE curves of $Pt_{20}Pd_{40}Cu_{40}$/C catalyst before cycling and after 10 K, 20 K, 30 K, and 40 K cycles, respectively. **b**, The corresponding MA and SA at 0.900 V (vs RHE) for the ORR. **c**, RDE curves of $Pd_{50}Cu_{50}$/C catalyst before cycling and after 1 K cycles[10]. **d**, The corresponding MA values at 0.900 V (vs RHE) for the ORR. **e**, RDE curves of E-tek Pt/C catalyst before cycling, after 20 K cycles, and after 40 K cycles. **f**, The corresponding MA values at 0.900 V (vs RHE) for the ORR[11]. (RDE scan rate: 10 mV; ADT scan rate: 100 mV/s; potential cycle window: 0.6 and 1.0 V; Electrolyte: 0.1 M $HClO_4$ saturated with oxygen; RDE rotating speed: 1600 RPM.)

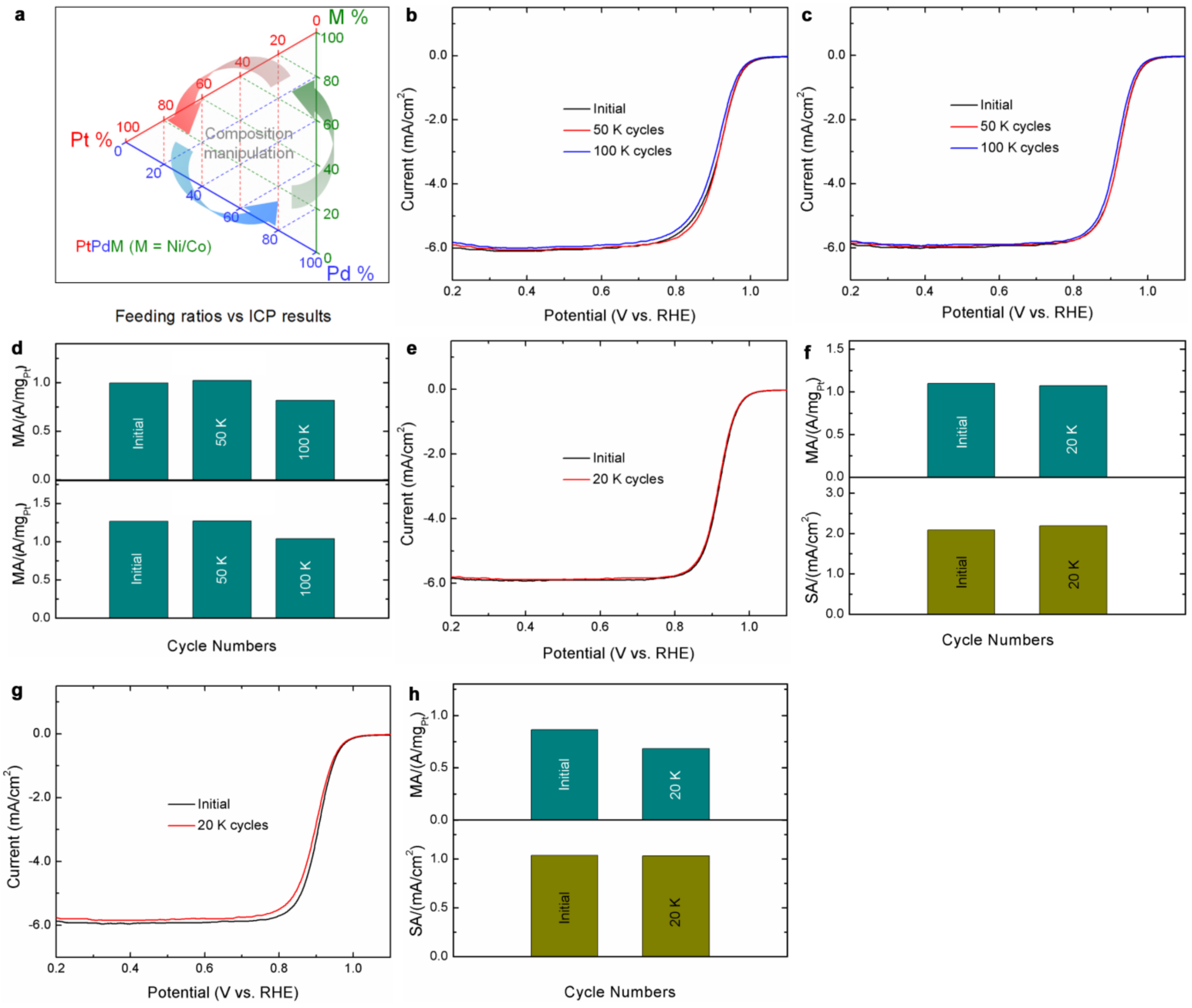

Supplementary Fig. 6 | The Design Concept of Low-Noble-Metal-Content Alloy Catalysts (PtPdM (M= Ni, Co)) and Their Activity and Durability for ORR. a, Triangle plot to demonstrate the composition manipulation of PtPdM (M = Ni/Co) nanoparticles. **b**, RDE curves of $Pt_{20}Pd_{40}Ni_{40}/C$, **c**, RDE curves of $Pt_{20}Pd_{40}Co_{40}/C$ catalysts before cycling, after 50 K, and after 100 K cycles, respectively. **d**, The corresponding MA values at 0.900 V (vs RHE) of $Pt_{20}Pd_{40}Ni_{40}/C$ (top) and $Pt_{20}Pd_{40}Co_{40}/C$ (bottom) catalysts during accelerated durability test. **e**, RDE curves of $Pt_{20}Pd_{20}Co_{60}/C$ catalyst before cycling and after 20 K cycles. **f**, The corresponding MA and SA at 0.900 V (vs RHE) for the ORR. **g**, RDE curves of $Pt_{20}Pd_{20}Ni_{60}/C$ catalyst before cycling and after 20 K cycles. **h**, The corresponding MA and SA at 0.900 V (vs RHE) for the ORR. (RDE scan rate: 10 mV; ADT scan rate: 100 mV/s; potential cycle window: 0.6 and 1.0 V; Electrolyte: 0.1 M $HClO_4$ saturated with oxygen; RDE rotating speed: 1600 RPM.)

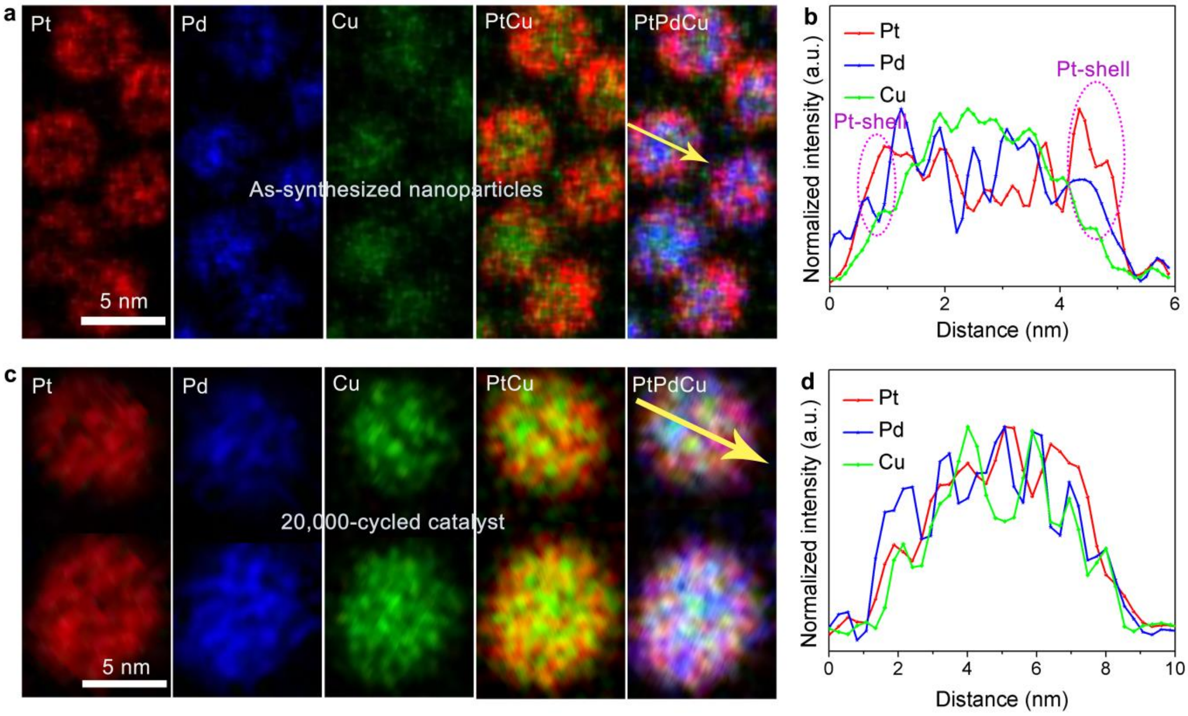

Supplementary Fig. 7 | Metal Component Distributions in the as-synthesized $Pt_{20}Pd_{20}Cu_{60}$ NPs and the 20,000-cycled $Pt_{20}Pd_{20}Cu_{60}/C$ catalysts. **a**, STEM-EELS elemental mapping of Pt (in red), Pd (in blue), Cu (in green), the integrated mapping of Pt and Cu, and the integrated mapping of Pt, Pd, and Cu for the as-synthesized $Pt_{20}Pd_{20}Cu_{60}$ NPs. **b**, The corresponding EELS line-scan profiles and the highlighted Pt-shell regions. **c**, Additional representative STEM-EELS elemental mapping of Pt (in red), Pd (in blue), Cu (in green), the integrated mapping of Pt and Cu, and the integrated mapping of Pt, Pd, and Cu for the 20,000-cycled $Pt_{20}Pd_{20}Cu_{60}/C$ catalyst. **d**, The corresponding EELS line-scan profiles. The exact atomic compositions determined by EDS are $Pt_{17}Pd_{23}Cu_{60}$ and $Pt_{27}Pd_{27}Cu_{46}$ for the as-synthesized $Pt_{20}Pd_{20}Cu_{60}$ NPs and the 20,000-cycled $Pt_{20}Pd_{20}Cu_{60}/C$ catalyst, respectively.

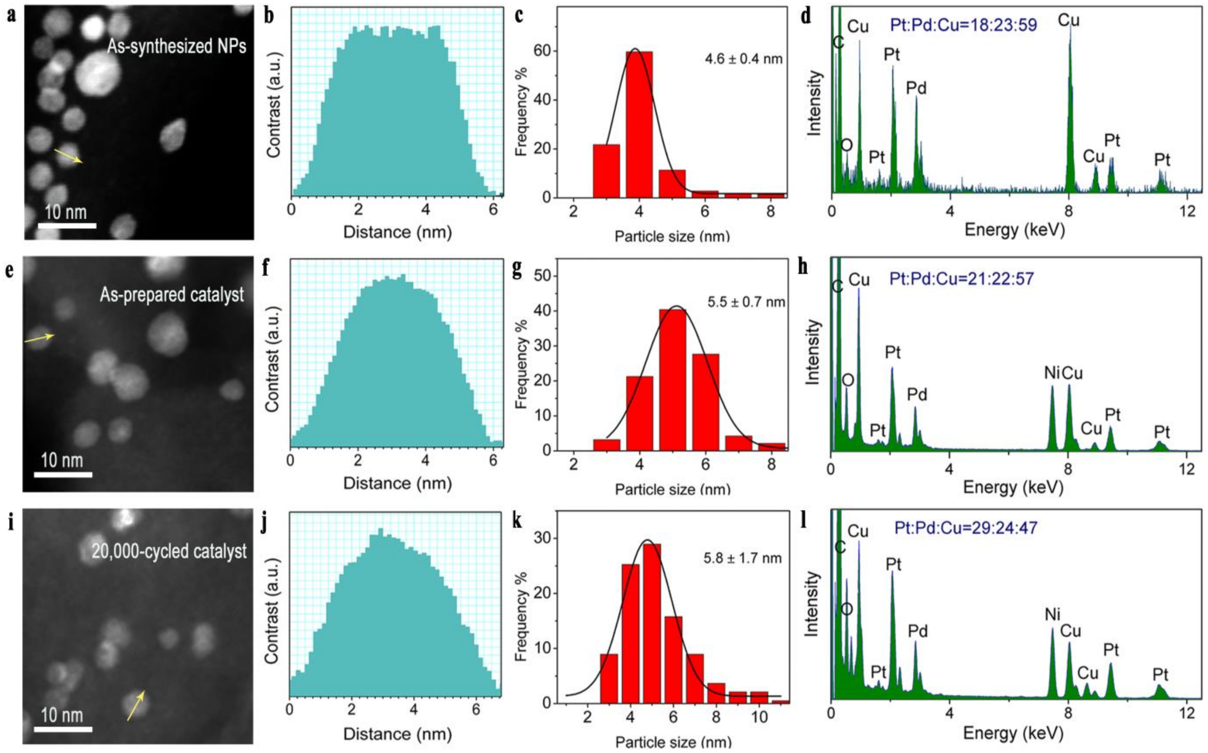

Supplementary Fig. 8 | HAADF images and the corresponding analyses of $Pt_{20}Pd_{20}Cu_{60}$ NPs in different states. **a**, **e**, **i**, HAADF-STEM images; **b**, **f**, **j**, contrast profiles measured along with the yellow arrows; **c**, **g**, **k**, metallic particle size distribution plots; and **d**, **h**, **l**, EDX spectra for **a-d** the as-synthesized $Pt_{20}Pd_{20}Cu_{60}$ NPs; **e-h** the as-prepared $Pt_{20}Pd_{20}Cu_{60}$/C catalyst after thermochemical treatment; and **i-l** the 20,000-cycled $Pt_{20}Pd_{20}Cu_{60}$/C catalyst.

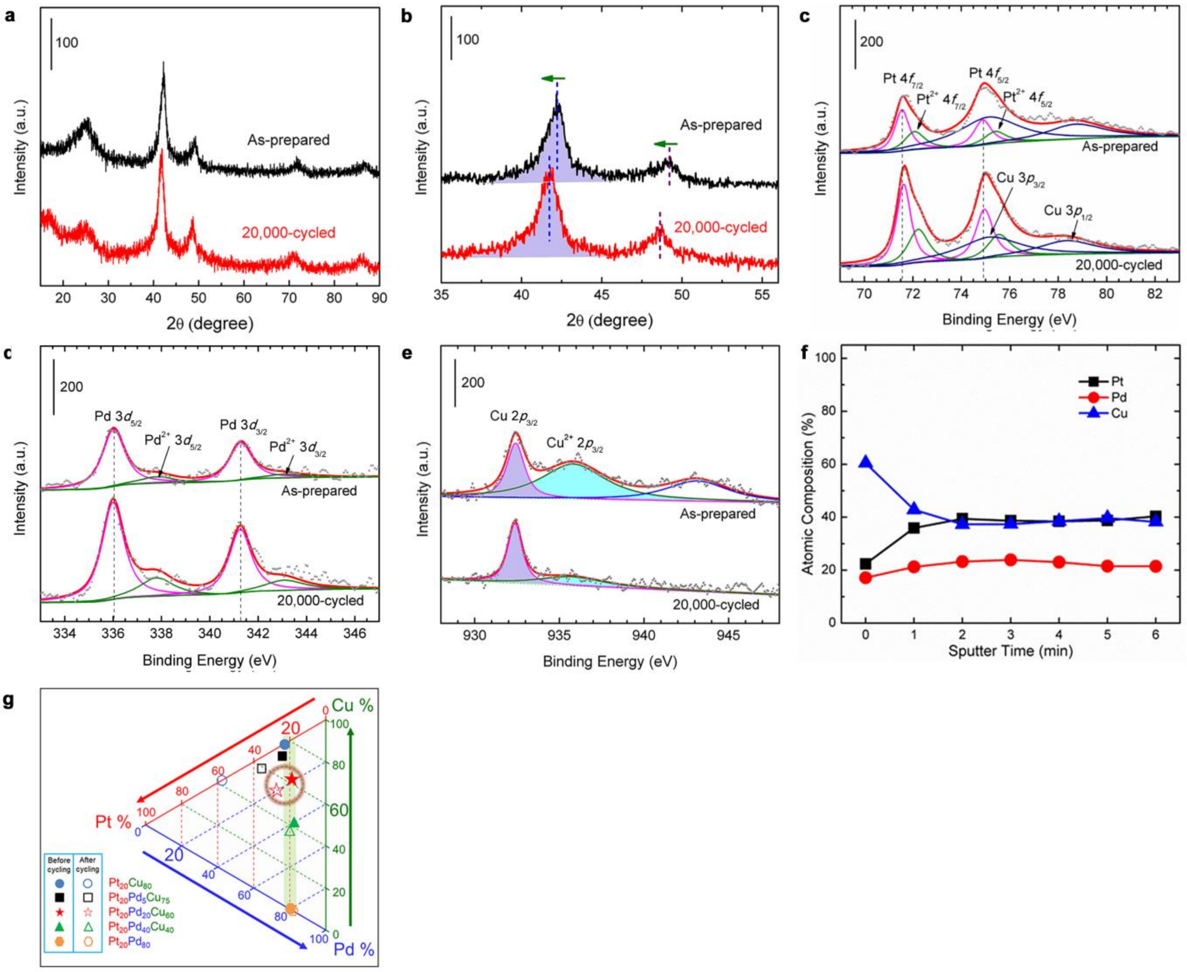

Supplementary Fig. 9 | Physical characterizations of $Pt_{20}Pd_{20}Cu_{60}$/C catalysts before and after 20,000 potential cycles. **a**, XRD patterns of the as-prepared and the 20,000-cycled $Pt_{20}Pd_{20}Cu_{60}$/C catalysts. **b**, A magnified view of the major features in panel **a** for a better visual comparison. **c-e**, XPS spectra and deconvoluted peaks for the as-prepared and the 20,000-cycled $Pt_{20}Pd_{20}Cu_{60}$/C catalysts in regions of **c**, Pt 4*f*, **d**, Pd 3*d*, and **e**, Cu 2*p*. **f**, Plot of the compositions of the 20,000-cycled $Pt_{20}Pd_{20}Cu_{60}$/C catalysts during Ar ion sputtering as a function of the sputtering time. We also examined $Pt_{20}Pd_nCu_{80-n}$/C catalysts with different compositions by XPS (see Supplementary Table 7). **g**, Plots of Pt, Pd and Cu atomic percentages in $Pt_{20}Pd_nCu_{80-n}$ NPs determined by ICP-OES before (solid symbols) and after 20,000 cycles ADT test (hollow symbols). The results gave $Pt_{58}Cu_{42}$, $Pt_{37}Pd_4Cu_{59}$, $Pt_{27}Pd_{20}Cu_{53}$, $Pt_{20}Pd_{43}Cu_{37}$, and $Pt_{19}Pd_{81}$, respectively, corresponding to the initial ICP results of $Pt_{22}Cu_{78}$, $Pt_{23}Pd_4Cu_{73}$, $Pt_{19}Pd_{19}Cu_{62}$, $Pt_{19}Pd_{39}Cu_{42}$, and $Pt_{20}Pd_{80}$, respectively.

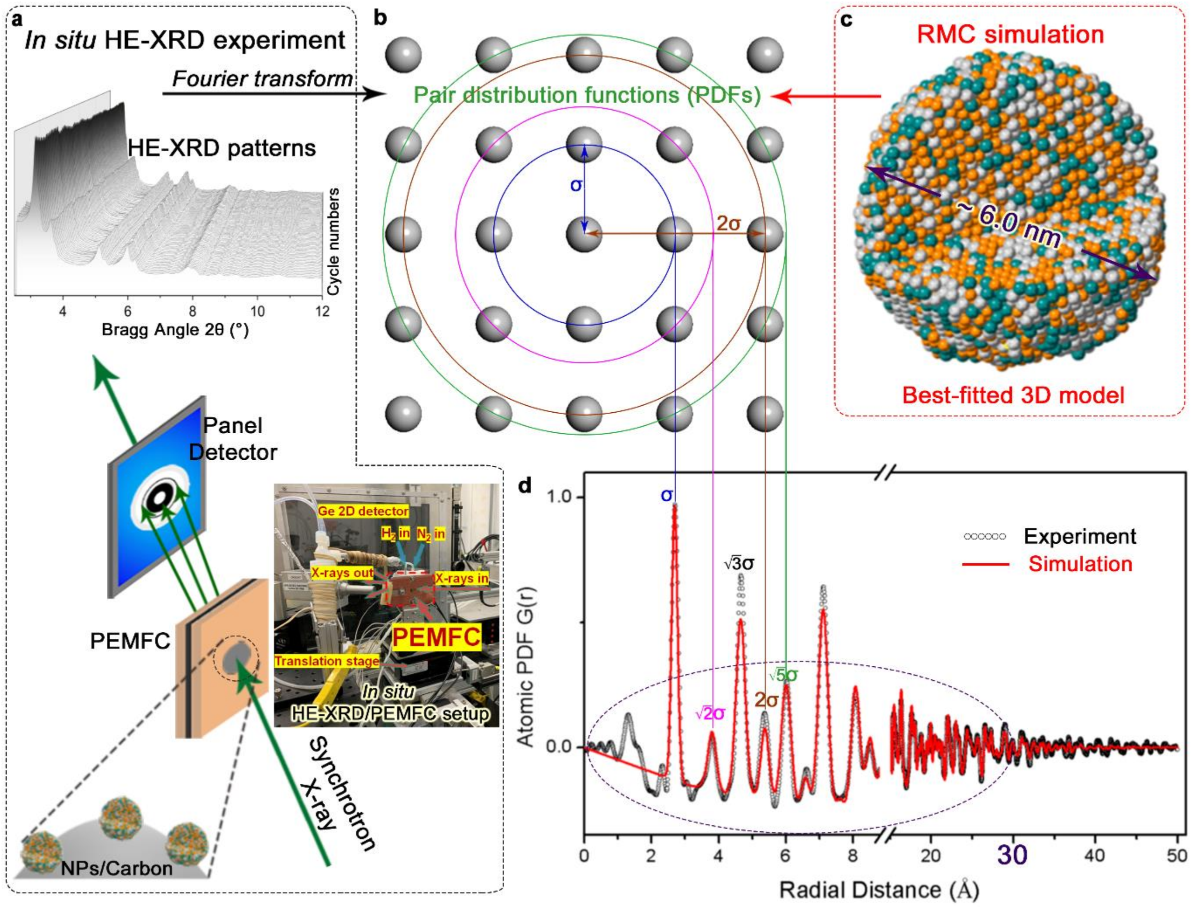

Supplementary Fig. 10 | Schematic illustrations of the in-situ HE-XRD/PEMFC experimental set up, PDF analysis, and RMC simulation for the catalyst structural characterization[12]. a, Experiment setup: **s**ynchrotron X-ray HE-XRD study of catalyst in PEMFC device and HE-XRD pattern collected. **b**, PDF analysis. **c**, RMC simulation: an example showing a best-fitted 3D RMC simulation model. **d**, A comparison of atomic PDFs generated from HE-XRD experiments (black symbols) and RMC simulations (red lines). The best-fitted 3D modeling provides the structure information of the catalyst.

As shown in Supplementary Fig. 10a, high-energy (> 100 keV) synchrotron X-rays penetrate through the catalyst MEA in a custom-designed PEMFC device[12]. The signals are collected by a panel detector and are transformed to HE-XRD patterns. The data are analyzed by PDFs technique (Supplementary Fig. 10b), in which the numbers of atom pairs in the first coordination shell constitute the first (σ) strong peak in the atomic PDF pattern (blue lines), that in the second coordination shell constitute the second peak in the atomic PDF pattern, and so on. Detailed structures of the catalysts are analyzed by 3D RMC models, revealing lattice parameters, atomic coordination numbers, nanophase contents and distributions, and atomic compositions and distributions, etc. (Supplementary Fig. 10c)[8]. The PDFs converted from experimental HE-XRD patterns (black symbols) and 3D RMC simulations (read lines) are depicted in Supplementary Fig. 10d. The best fitting between the experimental and the simulative PDFs produces the most accurate information extracted from the best-fitted 3D RMC models. The major PDF oscillations gradually calm down at a radial distance of ~30 Å in Supplementary Fig. 10d, corresponding to the NP size which is about 6 nm in the data as shown (Supplementary Fig. 10c)[12].

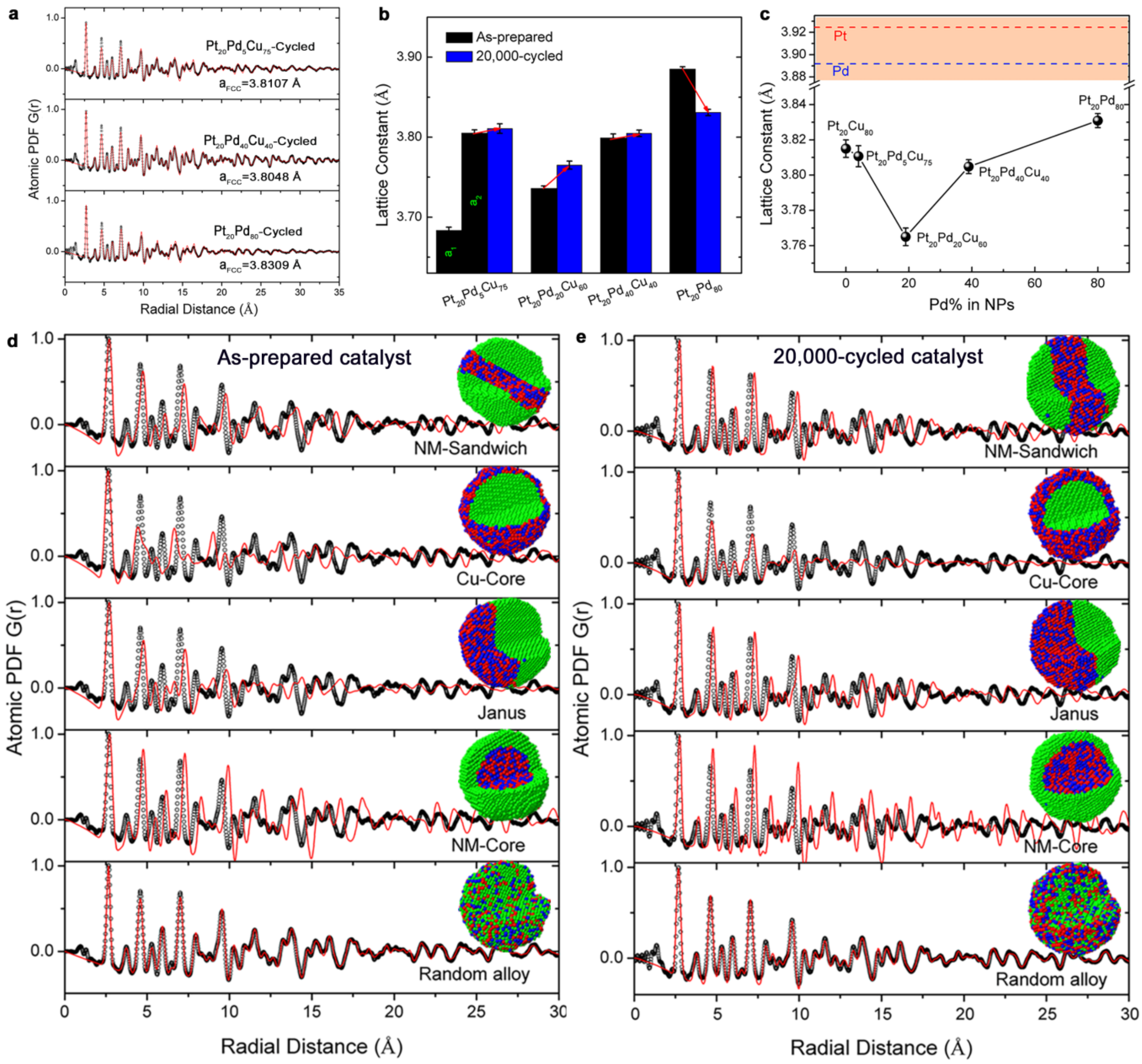

Supplementary Fig. 11 | Ex situ experimental HE-XRD data and analyzed results of PtPdCu/C catalysts before and after potential cycling. a, Experimental curves (black symbols) and phase structure model-derived atomic PDFs (red lines) for other 20,000-cycled PtPdCu/C and PtPd/C catalysts. **b**, Lattice constants of $Pt_{20}Pd_nCu_{80-n}$/C catalysts before and after 20,000 potential cycles. **c**, Plot of the lattice parameters of the 20,000-cycled $Pt_{20}Pd_nCu_{80-n}$/C (n=0, 5, 20, 40, 80) catalysts. Error bars represent s.d. based on three independent experiments in b and c. **d**, **e**, HE-XRD experimental curves (black symbols) and reverse Monte Carlo model computed atomic PDFs (red lines) for **d**, the as-prepared and **e**, the 20,000-cycled $Pt_{20}Pd_{20}Cu_{60}$/C catalyst

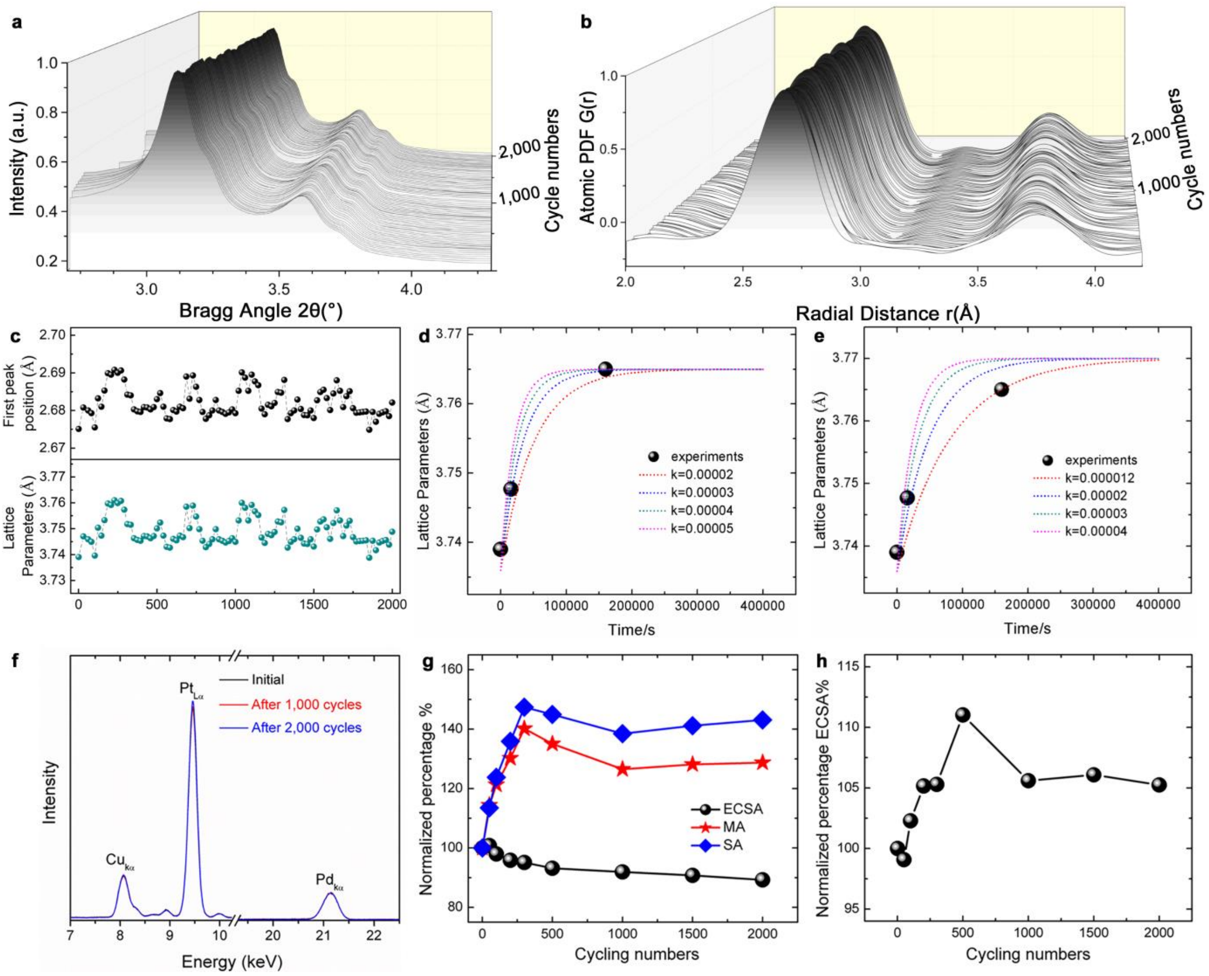

Supplementary Fig. 12 | Data collected from in situ/operando HE-XRD measurements of PtPdCu/C catalyst in MEA in an PEMFC cell under operating condition. **a**, A magnified view of the *in operando* HE-XRD patterns and **b**, the corresponding atomic PDFs obtained by Fourier transform for $Pt_{20}Pd_{20}Cu_{60}$/C catalyst inside an operating PEMFC upon potential cycling. **c**, The close examination of the first peak positions from the atomic PDFs in b and the calculated lattice constants as a function of potential cycling number. **d**, **e**, Simulations of the kinetics of the lattice constants (LC) extracted from the HE-XRD patterns for $Pt_{20}Pd_{20}Cu_{60}$/C catalyst during potential cycling based on first-order reaction model. **d**, LC = 3.736 + 0.029(1-exp(-kt)), and **e**, LC = 3.736 + 0.034(1-exp(-kt)). Apparently, Model A with k= $4x10^{-5}$ s^{-1} fits all data points, whereas Model B does not. **f**, EDX spectra during in-situ potential cycling of the $Pt_{20}Pd_{20}Cu_{60}$/C catalyst in MEA assembled in a custom-designed fuel cell. **g**, Normalized ECSA, MA, and SA values from in-house RDE measurements of $Pt_{20}Pd_{20}Cu_{60}$/C catalyst, mimicking the in-situ experimental condition. **h**, Normalized ECSA values obtained from in-situ fuel cell measurements of $Pt_{20}Pd_{20}Cu_{60}$/C catalyst.

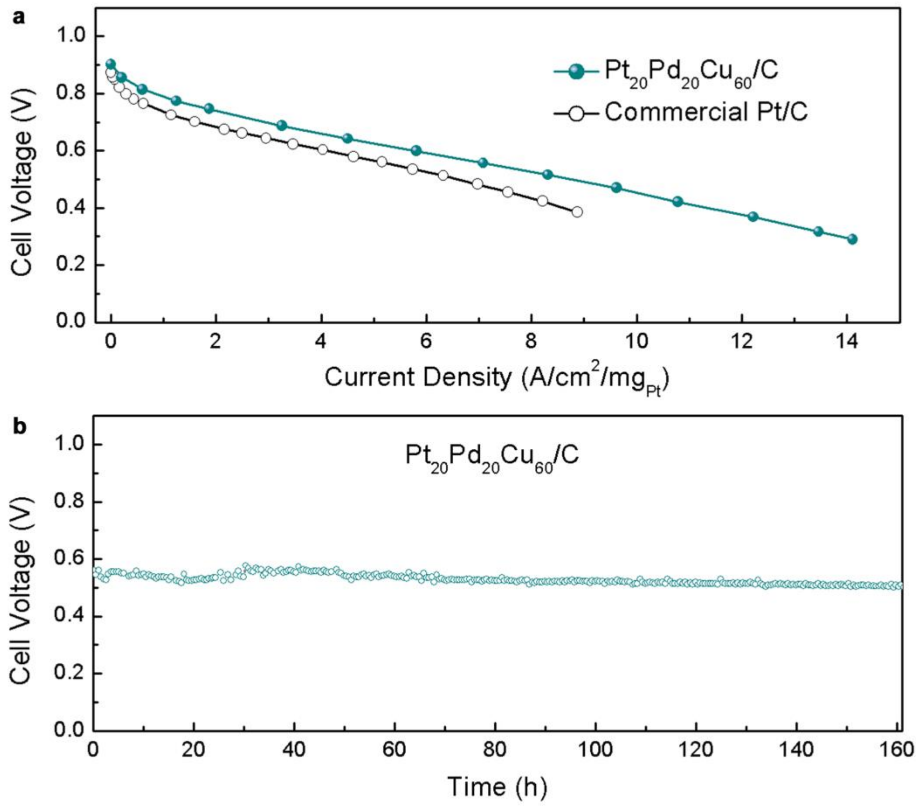

Supplementary Fig. 13 | Data collected from testing PtPdCu/C and commercial Pt/C catalyst in MEA in an PEMFC. a, H_2–O_2 fuel cell polarization plots with the MEAs prepared using $Pt_{20}Pd_{20}Cu_{60}/C$ and commercial Pt/C as the cathode catalysts. **b**, Stability test: fuel cell voltage vs. time at 1.0 A/cm^2 for the MEA with $Pt_{20}Pd_{20}Cu_{60}/C$ as the cathode catalyst. (Anode: commercial Pt/C). Note that normalized current density was used in this plot because of the difference in catalyst loading between MEAs with Pt/C (0.34 mg$_{Pt}$/cm^2) and $Pt_{20}Pd_{20}Cu_{60}/C$ (0.15 mg$_{Pt}$/cm^2) as the cathode catalysts.

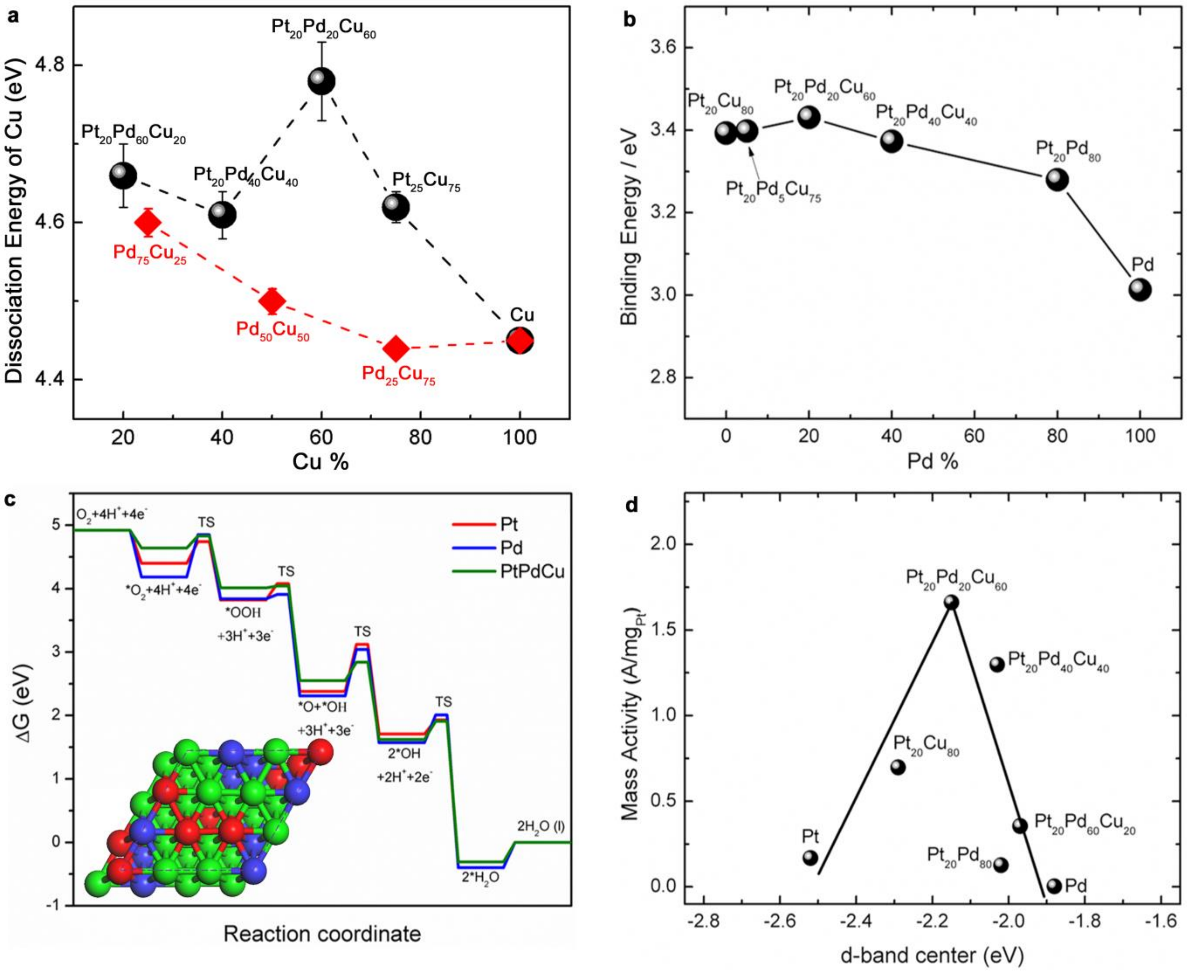

Supplementary Fig. 14 | The theoretical modeling of enhanced stability and activity of the ternary alloy catalyst. a, Plots of the dissociation energies of Cu vs. the Cu atomic percentage in PdCu, PtCu, and PtPdCu slab models with different compositions. **b,** Plot of the binding energies vs the compositions for PtPdCu, PtCu, PtPd and Pd clusters. A set of 1.5 nm cluster models with 116 atoms with different compositions was built. Pure Pt cluster shows the highest binding energy (4.48 eV) while pure Pd and Cu give the lowest binding energies (3.01 and 3.00 eV). By fixing Pt at 20% in Pt-alloys while varying the compositions of Pd and Cu, maximum binding energy was achieved for $Pt_{20}Pd_{20}Cu_{60}$ cluster. The optimized metal binding energies may be an important factor for good durability. **c**, Free energy diagram comparing the energetics for the different ORR species and the reaction barriers for some key elementary steps in ORR. The calculations were conducted on Pt (111), Pd (111), and PtPdCu (111) surface models at 0 V with respect to computational hydrogen electrode. Insert in c is the slab surface model of $Pt_{20}Pd_{20}Cu_{60}$. Note: the red, blue, and green balls denote the Pt, Pd, and Cu atoms, respectively. **d**, Plots of the mass activity vs. the d-band centers on the catalysts' (111) surfaces. The mass activity is apparently maximized at $Pt_{20}Pd_{20}Cu_{60}$ with a d-band center close to -2.2 eV. According to the d-band theory[13], Pt-based alloy can negatively tune the d-band center of Pt by around 0.2-0.3 eV, e.g., -2.8 eV. In comparison with Pt-skin alloy model, our full alloy model shows a reverse positively shift of the d-band center by 0.3 eV, e.g., -2.2 eV. Alloying with Pd apparently increases the d-band center of Pt.

Supplementary Table 1. Comparisons of structures, compositions, preparations, and catalytic properties of relevant catalysts reported in the recent literature.

Catalysts	Composition	Catalysts treatment	Mass Activity	Durability	Refs
PtPdCu NPs	$Pt_{27}Pd_{20}Cu_{53}$	Annealing under 20% O_2 at 260 °C and 15% H_2 at 400 °C in sequence.	1.08 A mg_{Pt+Pd}^{-1}	Negligible MA decay after 50,000 cycles	This work
$PdCu_2$@Pt core-shell NPs	$Pt_{13.0}Pd_{41.9}Cu_{45.1}$	Synthesize $PdCu_2$/C core first, then anneals it at 500 °C in 10% H_2 (balanced by N_2) for 2h. Deposit Pt as the shell.	0.485 A mg_{Pt+Pd}^{-1}	Negligible MA decay after 10,000 cycles	14
Hollow PtPdCu NPs	$Pt_{37}Pd_{41}Cu_{22}$	NPs supported on carbon then acid treated (dealloying).	0.61 A mg^{-1}	Negligible MA decay after 10,000 cycles	15
PtPdCu nanodendrite	$Pt_{14}Pd_{80}Cu_{6}$	Deposit Pt-shell on PdCu nanodendrites.	0.42 A mg_{Pt+Pd}^{-1} (at 0.85 V vs. RHE)	64% MA remains after 5,000 cycles	16
PtPdCu nanoparticle nanotubes	$Pt_{32.6}Pd_{15.4}Cu_{52}$	PtPdCu NPs on Cu nanowires; treated by electrochemical dealloying.	0.52 A mg_{Pt}^{-1}; 0.33 A mg_{Pt+Pd}^{-1}.	88.3% MA remains after 30,000 cycles	17
PtPdCu nanoframe	$Pt_{74.7}Pd_{18.6}Cu_{6.7}$	Catalysts treated by acid etching.	1.04 A mg_{Pt}^{-1}	MA decay after 10,000 cycles	18
Pd@PtNi core-shell octahedra	$Pt_1Pd_1Ni_{0.55}$	Catalysts treated by acid washing, heat at 60 °C, then wash with ethanol.	2.5 A mg_{Pt}^{-1}; 1.6 A mg_{Pt+Pd}^{-1}.	1.7% MA decays after 10,000 cycles	19
Pd@Pt octahedra	$Pt_1Pd_{3.73}$	Catalysts treated by acid etching.	1.05 A mg_{Pt}^{-1}	35% MA decays after 20,000 cycles	20
Pt_3Ni nanoframe	Pt_3Ni	Intial thermal treatment, andannealing in inert gas below 400 °C for 12h.	5.7 A mg_{Pt}^{-1}	Negligible MA decay after 10,000 cycles	21
Mo-doped Pt_3Ni octahedral	$Pt_{73.9}Ni_{24.5}Mo_{1.6}$	Wash the catalysts by ethanol/acetone mixture.	6.9 A mg_{Pt}^{-1}	5.5% MA decays after 8,000 cycles	22
PtPb@Pt nanoplate	$Pt_{55.9}Pb_{44.1}$	After assembly, anneal the catalyst at 250 °C at atmosphere for 1h.	4.3 A mg_{Pt}^{-1}	7.7% MA decays after 50,000 cycles	23
PtNi nanocages	$Pt_{81}Ni_{19}$	As-synthesized nanocages undergo acid washing and then assembly on carbon support	3.52 A mg_{Pt}^{-1}	Negligible MA decay after 50,000 cycles	24
PtNiCu NPs	$Pt_{82.6}Cu_{11.9}Ni_{5.5}$	Two-step wet chemical synthesis without post-treatments	3.7 A mg_{Pt}^{-1}	30.7% MA decays after 30,000 cycles	25

Supplementary Table 2. Comparisons of the atomic compositions and their changes before and after electrochemical durability tests of the catalysts studied in this work. (NM: noble metal. NNM: non-noble metal.)

Sample name	Feeding ratio	ICP composition (As-prepared)	ICP composition (Cycled)	NM:NNM (As-prepared)	NM:NNM (Cycled)	NNM% change
PtPdCu*	20:20:60	19:19:62	27:20:53	38:62	47:53	9
PtPdCu*	20:40:40	19:39:42	20:43:37	58:42	63:37	5
PtPdCu*	22:5:75	23:4:73	37:4:59	27:73	41:59	14
PtPd*	20:80	20:80	19:81	/	/	/
PtPdCo*	20:40:40	22:43:35	28:45:27	65:35	73:27	8
PtPdNi*	20:40:40	17:42:41	31:42:27	59:41	73:27	14
PtCu*	20:80	22:78	58:42	22:78	58:42	36
PtFeCu**	20:40:40	24:17:59	63:16:21	24:76	63:37	39
PtFeCo**	20:40:40	37:17:46	76:2:22	37:63	76:24	39
PtFeNi**	20:40:40	31:23:46	78:9:13	31:69	78:22	47
PtNiCo**	20:40:40	19:39:42	55:23:22	19:81	55:45	36
PtCuCo**	20:40:40	18:43:39	84:9:7	18:82	84:16	66
PtCuNi**	20:40:40	17:41:42	74:15:11	17:83	74:26	57
PtFe**	20:80	30:70	55:45	30:70	55:45	25
PtNi**	20:80	20:80	44:56	20:80	44:56	24
PtCo**	20:80	25:75	74:26	25:75	74:26	49

Note: * Catalysts tested 20,000 potential cycles;

** Catalysts tested 10,000 potential cycles.

Supplementary Table 3. Comparisons of the structures, compositions, and catalytic properties of the relevant PtPd catalysts reported in the recent literature.

Catalysts	Composition	Mass Activity	Durability	Refs
PtPdCu NPs	$Pt_{19}Pd_{19}Cu_{62}$	1.08 A mg_{Pt+Pd}^{-1}	Negligible MA decay after 50,000 cycles	This work
PtPd nanowire	$Pt_{78}Pd_{22}$	0.92 A mg_{Pt+Pd}^{-1}	9% MA decays after 8,000 cycles	26
PtPd nanocubes	$Pt_{32}Pd_{68}$	0.38 A mg_{Pt+Pd}^{-1}	46% MA decays after 15,000 cycles	27
PtPdCo nanorings	$Pt_{47}Pd_{41}Co_{12}$	3.58 A mg_{Pt}^{-1} (0.1 M KOH)	29% MA decay after 30,000 cycles	28
Pt~Pd nanowire	N/A	0.55 A mg_{PGM}^{-1}	37% ECSA and 3 mV half wave potential loss after 30,000 cycles	29
PdCu@Pt monolayer	Pt:Pd~1:5 (mass)	0.44 A mg_{PGM}^{-1}	~36% MA loss after 5,000 cycles	30
Pd@Pt	Pt:Pd~1:1.5 (mass)	0.38 A mg_{PGM}^{-1}	18% MA loss after 10,000 cycles	31
Pd-Pt nanodentrites	Pt:Pd~5.6:1 (mass)	0.20 A mg_{PGM}^{-1}	50% ECSA loss after 10,000 cycles	32
Pd@Pt octahedra	$Pt_1Pd_{3.73}$	0.35 A mg_{PGM}^{-1}	35% MA loss after 20,000 cycles	33
Pt-Pd nanocages	N/A	0.75 A mg_{Pt}^{-1}	36% MA loss after 10,000 cycles	34
PtPd NPs	$Pt_{14}Pd_{86}$	0.20 A mg_{PGM}^{-1}	N/A	35
PtPd alloy	Pt_1Pd_1	0.49 A mg_{Pt}^{-1}	3.5% MA loss after 10,000 cycles	36

Supplementary Table 4. Comparisons of metal combinations, compositions, and catalytic properties of other relevant Pt-based ternary catalysts reported in the recent literature.

Catalysts	Composition (initial)	Mass Activity	Durability	Refs
PtPdCu NPs	$Pt_{19}Pd_{19}Cu_{62}$	1.66 A mg_{Pt}^{-1}	Negligible MA decay after 50,000 cycles	This work
Rh-doped PtNi NPs	$Pt_{71}Ni_{26}Rh_{3}$	0.82 A mg_{Pt}^{-1}	61.0% MA decay after 30,000 cycles	37
PtNiIr yolk-shell NPs	$Pt_{59.6}Ni_{18.4}Ir_{22.0}$	0.67 A mg_{Pt}^{-1}	22.8% MA decay after 10,000 cycles	38
PtNiAu NPs	$Pt_{57}Ni_{23}Au_{20}$	0.83 A mg_{Pt}^{-1}	16.8% MA decay after 10,000 cycles	39
PtPbNi octahedral NPs	$PtPb_{1.12}Ni_{0.14}$	1.92 A mg_{Pt}^{-1}	17.2% MA decay after 15,000 cycles	40
PtCuW NPs	$Pt_{2}CuW_{0.25}$	0.75 A mg_{Pt}^{-1}	4.1% MA decay after 30,000 cycles	41
Ga-doped PtCo NWs	$Pt_{72}Co_{24}Ga_{4}$	0.67 A mg_{Pt}^{-1}	4.7% MA decay after 20,000 cycles	42
PtNiIn NPs	$Pt_{47}Ni_{50}In_{4}$	0.76 A mg_{Pt}^{-1}	23.2% MA decay after 16,000 cycles	43
Ga-doped PtNi NWs	$Pt_{60}Ni_{38}Ga_{1.9}$	1.24 A mg_{Pt}^{-1}	65.5% MA decay after 30,000 cycles	44
PtNiCo NPs	$Pt_{46}Ni_{25}Co_{29}$	3.1 A mg_{Pt}^{-1}	15.9% MA decay after 30,000 cycles	45

Supplementary Table 5 | The reaction barriers (unit: eV) of some key elementary steps involved in the ORR on Pt (111), Pd (111), and PtPdCu (111) surfaces.

	Pt (111)	Pd (111)	PtPdCu (111)
$*O_2 + *H \rightarrow *OOH + *$	0.34	0.67	0.19
$*OOH + * \rightarrow *OH + *O$	0.26	0.07	0.03
$*O + *H \rightarrow *OH + *$	0.74	0.73	0.29
$*OH + *H \rightarrow *H_2O + *$	0.22	0.44	0.29
$*O_2 + * \rightarrow *O + *O$	0.69	0.72	0.26

Supplementary Table 6 | Binding energies (unit: eV) of some key intermediates on Pt (111), Pd (111), and PtPdCu (111) surfaces.

	Pt (111)	Pd (111)	PtPdCu (111)
O_2	0.52	0.74	0.28
OOH	1.13	1.22	1.23
O	3.99	4.18	4.05[a]<br>3.76[b]
OH	2.17	2.37	2.38
H_2O	0.29	0.29	0.26
H	2.63	2.57	2.45

[a]. On PdCuCu fcc site;
[b]. On PtPtPd fcc site.

Supplementary Table 7 | XPS peak positions (unit: eV) for $Pt_{20}Pd_nCu_{80-n}$/C catalysts. The deconvoluted XPS peak positions for $Pt_{20}Pd_nCu_{80-n}$/C catalysts, including Pt 4$f_{7/2}$, and 4$f_{5/2}$, Pd 3$d_{5/2}$, and Pd 3$d_{3/2}$, Cu 2$p_{3/2}$, and Cu 2$p_{1/2}$.

XPS peak	$Pt_{20}Cu_{80}$	$Pt_{20}Pd_5Cu_{75}$	$Pt_{20}Pd_{20}Cu_{60}$	$Pt_{20}Pd_{40}Cu_{40}$	$Pt_{20}Pd_{60}Cu_{20}$
Pt^0 4$f_{7/2}$	71.35	71.25	71.13	71.23	71.11
Pt^{2+} 4$f_{7/2}$	72.00	71.84	71.85	72.25	72.35
Pt^0 4$f_{5/2}$	74.56	74.55	74.43	74.44	74.50
Pt^{2+} 4$f_{5/2}$	76.50	76.50	76.17	76.50	76.36
Pd^0 3$d_{5/2}$	---	336.02	335.89	335.86	335.65
Pd^{2+} 3$d_{5/2}$	---	---	337.20	336.86	336.95
Pd^0 3$d_{3/2}$	---	341.28	341.15	341.12	340.91
Pd^{2+} 3$d_{3/2}$	---	---	342.46	342.12	342.21
Cu^0 2$p_{3/2}$	933.16	933.19	932.98	932.90	932.65
Cu^{2+} 2$p_{3/2}$	935.53	935.24	935.30	935.34	935.12
Cu^0 2$p_{1/2}$	952.96	953.09	952.78	952.70	952.78
Cu^{2+} 2$p_{1/2}$	955.33	955.45	955.10	955.14	954.80

Supplementary References

1 Guisbiers, G. & Buchaillot, L. Size and shape effects on creep and diffusion at the nanoscale. *Nanotechnology* **19**, 435701 (2008).

2 Schrøder, T. B. & Dyre, J. C. Solid-like mean-square displacement in glass-forming liquids. *J. Chem. Phys.* **152**, 141101 (2020).

3 Xiong, G., Clark, J. N., Nicklin, C., Rawle, J. & Robinson, I. K. Atomic Diffusion within Individual Gold Nanocrystal. *Sci. Rep.* **4**, 6765 (2014).

4 Surrey, A., Pohl, D., Schultz, L. & Rellinghaus, B. Quantitative Measurement of the Surface Self-Diffusion on Au Nanoparticles by Aberration-Corrected Transmission Electron. *Nano Lett.* **12**, 6071–6077 (2012).

5 Rice, K. P., Paterson, A. S. & Stoykovich, M. P. Nanoscale Kirkendall Effect and Oxidation Kinetics in Copper Nanocrystals Characterized by Real-Time, In Situ Optical Spectroscopy. *Part. Part. Syst. Charact.* **32**, 373–380 (2015).

6 Schneider, S., Surrey, A., Pohl, D., Schultz, L. & Rellinghaus, B. Atomic surface diffusion on Pt nanoparticles quantified byhigh-resolution transmission electron microscopy. *Micron* **63**, 52–56 (2014).

7 Gereben, O. & Petkov, V. Reverse Monte Carlo study of spherical sample under non-periodic boundary conditions: the structure of Ru nanoparticles based on x-ray diffraction data. *J. Phys. Condens. Matter* **25**, 454211 (2013).

8 Petkov, V. *et al.* Solving the nanostructure problem: exemplified on metallic alloy nanoparticles. *Nanoscale* **6**, 10048–10061 (2014).

9 Shan, S. *et al.* Surface oxygenation of multicomponent nanoparticles toward active and stable oxidation catalysts. *Nat. Commun.* **11**, 4201 (2020).

10 Wu, Z. P. *et al.* Revealing the role of phase structures of bimetallic nanocatalysts in the oxygen reduction reaction. *ACS Catal.* **8**, 11302–11313 (2018).

11 Kong, Z. *et al.* Origin of high activity and durability of twisty nanowire alloy catalysts under oxygen reduction and fuel cell operating conditions. *J. Am. Chem. Soc.* **142**, 1287–1299 (2020).

12 Wu, Z. P., Shan, S., Zang, S. Q. & Zhong, C. J. Dynamic Core–Shell and Alloy Structures of Multimetallic Nanomaterials and Their Catalytic Synergies. *Acc. Chem. Res.* **53**, 2913–2924 (2020).

13 Stamenkovic, V. *et al.* Changing the activity of electrocatalysts for oxygen reduction by tuning the surface electronic structure. *Angew. Chem. Int. Ed.* **45**, 2897-2901 (2006).

14 Park, H. Y. *et al.* Hollow $PdCu_2$@Pt core@shell nanoparticles with ordered intermetallic cores as efficient and durable oxygen reduction reaction electrocatalysts. *Appl. Catal. B Environ.* **225**, 84–90 (2018).

15 Liu, X. J. *et al.* Hollow ternary PtPdCu nanoparticles: a superior and durable cathodic electrocatalyst. *Chem. Sci.* **6**, 3038–3043 (2015).

16 Fu, S. *et al.* Low Pt-content ternary PdCuPt nanodendrites: an efficient electrocatalyst for oxygen reduction reaction. *Nanoscale* **9**, 1279–1284 (2017).

17 Li, H. H. *et al.* Mixed-PtPd-Shell PtPdCu Nanoparticle Nanotubes Templated from Copper Nanowires as Efficient and Highly Durable Electrocatalysts. *Adv. Energy Mater.* **2**, 1182–1187 (2012).

18 Ye, W. *et al.* $Pt_4PdCu_{0.4}$ alloy nanoframes as highly efficient and robust bifunctional electrocatalysts for oxygen reduction reaction and formic acid oxidation. *Nano Energy* **39**, 532–538 (2017).

19 Choi, S. II *et al.* Synthesis and Characterization of Pd@Pt-Ni Core-Shell Octahedra with High Activity toward Oxygen Reduction. *ACS nano* **8**, 10363–10371 (2014).

20 Zhou, M. *et al.* Quantitative Analysis of the Reduction Kinetics Responsible for the One-Pot Synthesis of Pd−Pt Bimetallic Nanocrystals with Different Structures. *J. Am. Chem. Soc.* **138**, 12263–12270 (2016).

21 Chen, C. *et al.* Highly crystalline multimetallic nanoframes with three-dimensional electrocatalytic surfaces. *Science* **343**, 1339–1343 (2014).

22 Huang, X. Q. *et al.* High-performance transition metal-doped Pt_3Ni octahedra for oxygen reduction reaction. *Science* **348**, 1230–1234 (2015).

23 Bu, L. *et al.* Biaxially strained PtPb/Pt core/shell nanoplate boosts oxygen reduction catalysis. *Science* **354**, 1410–1414 (2016).

24 Tian, X. L. *et al.* Engineering bunched Pt-Ni alloy nanocages for efficient oxygen reduction in practical fuel cells. *Science* **366**, 850–856 (2019).

25 Cao, L. *et al.* Differential Surface Elemental Distribution Leads to Significantly Enhanced Stability of PtNi-Based ORR Catalysts. *Matter* **1**, 1567–1580 (2019).

26 Chang, F. *et al.* Strain-Modulated Platinum−Palladium Nanowires for Oxygen Reduction Reaction. *Nano Lett.* **20**, 2416–2422 (2020).

27 Wu, R. Tsiakaras, P. & Shen, P. K. Facile synthesis of bimetallic Pt-Pd symmetry-broken concave nanocubes and their enhanced activity toward oxygen reduction reaction. *Appl. Catal. B Environ.* **251**, 49–56 (2019).

28 Sun, Y. *et al.* Ultrathin PtPd-based nanorings with abundant step atoms enhance oxygen catalysis. *Adv. Mater.* **30**, 1802136 (2018).

29 Koenigsmann, C. *et al.* Enhanced Electrocatalytic Performance of Processed, Ultrathin, Supported Pd–Pt Core–Shell Nanowire Catalysts for the Oxygen Reduction Reaction. *J. Am. Chem. Soc.* **133**, 9783–9795 (2011).

30 Shao, M. *et al.* Pt Monolayer on Porous Pd–Cu Alloys as Oxygen Reduction Electrocatalysts. *J. Am. Chem. Soc.* **132**, 9253–9255 (2010).

31 Zhang, L. *et al.* Palladium–Platinum Core–Shell Electrocatalysts for Oxygen Reduction Reaction Prepared with the Assistance of Citric Acid. *ACS Catal.* **6**, 3428–3432 (2016).

32 Lim, B. *et al.* Pd–Pt Bimetallic Nanodendrites with High Activity for Oxygen Reduction. *Science* **324**, 1302–1305 (2009).

33 Zhou, M. *et al.* Quantitative Analysis of the Reduction Kinetics Responsible for the One-Pot Synthesis of Pd–Pt Bimetallic Nanocrystals with Different Structures. *J. Am. Chem. Soc.* **138**, 12263–12270 (2016).

34 Zhang, L. *et al.* Platinum-based nanocages with subnanometer-thick walls and well-defined, controllable facets. *Science* **349**, 412–416 (2015).

35 Wu, J. *et al.* Understanding Composition-Dependent Synergy of PtPd Alloy Nanoparticles in Electrocatalytic Oxygen Reduction Reaction. *J. Phys. Chem. C* **121**, 14128–14136 (2017).

36 Wang, W. *et al.* Highly Active and Stable Pt-Pd Alloy Catalysts Synthesized by Room-Temperature Electron Reduction for Oxygen Reduction Reaction. *Adv. Sci.* **4**, 1600486 (2017).

37 Beermann, V. *et al.* Rh-Doped Pt−Ni Octahedral Nanoparticles: Understanding the Correlation between Elemental Distribution, Oxygen Reduction Reaction, and Shape Stability. *Nano Lett.* **16**, 1719–1725 (2016).

38 Yang, T. *et al.* Synthesis of octahedral Pt–Ni–Ir yolk–shell nanoparticles and their catalysis in oxygen reduction and methanol oxidization under both acidic and alkaline conditions. *Nanoscale* **11**, 23206–23216 (2019).

39 Liu, F. *et al.* Highly Durable and Active Ternary Pt–Au–Ni Electrocatalyst for Oxygen Reduction Reaction. *ChemCatChem* **10**, 3049–3056 (2018).

40 Bu, L. *et al.* PtPb/PtNi Intermetallic Core/Atomic Layer Shell Octahedra for Efficient Oxygen Reduction Electrocatalysis. *J. Am. Chem. Soc.* **139**, 9576–9582 (2017).

41 Tu, W. *et al.* Tungsten as “Adhesive” in Pt2CuW0.25 Ternary Alloy for Highly Durable Oxygen Reduction Electrocatalysis. *Adv. Funct. Mater.* **30**, 1908230 (2020).

42 Li, M. *et al.* Lavender-Like Ga-Doped Pt3Co Nanowires for Highly Stable and Active Electrocatalysis. *ACS Catal.* **10**, 3018–3026 (2020).

43 Shen, X. *et al.* Tuning Electronic Structure and Lattice Diffusion Barrier of Ternary Pt−In−Ni for Both Improved Activity and Stability Properties in Oxygen Reduction Electrocatalysis. *ACS Catal.* **9**, 11431–11437 (2019).

44 Lim, J. *et al.* Ga−Doped Pt−Ni Octahedral Nanoparticles as a Highly Active and Durable Electrocatalyst for Oxygen Reduction Reaction. *Nano Lett.* **18**, 2450–2458 (2018).

45 Li, J. *et al.* Anisotropic Strain Tuning of $L1_0$ Ternary Nanoparticles for Oxygen Reduction. *J. Am. Chem. Soc.* **142**, 19209–19216 (2020).
