# Supplementary material for: Alloying–realloying enabled high durability for Pt–Pd-3d-transition metal nanoparticle fuel cell catalysts
Source: Nat Commun. 2021 Feb 8;12:859. doi: 10.1038/s41467-021-21017-6 (PMC7870895; doi:10.1038/s41467-021-21017-6)
Supplement: Supplementary file 2 — Peer Review File [file 41467_2021_21017_MOESM2_ESM.pdf]

Reviewer #1 (Remarks to the Author):

This manuscript by Wu et al. demonstrated a durable ternary alloy catalyst by alloying PtPd (<50%) with Cu (Ni or Co). Dealloying-realloying of the alloy catalysts was observed during potential cycles by in-situ/operando synchrotron X-ray diffraction spectroscopy in combination with STEM/EELS and XPS analyses, as well as DFT modeling. The results are interesting and may provide useful information for design of stable and active alloy catalysts in fuel cells. Before possible consideration of acceptance in Nat. Commun., the following issues should be clarified.
1. The abbreviation MA should be defined when first used.
2. Corresponding metric should be added to the Y-axis of the top panel in Fig. 1b.
3. Did some Pd species as well as Cu leach into the electrolyte?
4. The evolution kinetics of re-alloying during ADT tests can be investigated.
5. To attain a stable Pt-based ternary alloy catalyst, what's the rule for selection of the second and third metal components? Further, is it possible to design an active ternary alloy ORR electrocatalyst without use of Pt based on this dealloying-re-alloying finding?
6. From the line-scan profiles shown in Fig. 3c and d, the composition of the two nanoparticles are not complete homogeneous. The authors need to comment this.
7. Comparisons between the ternary catalyst obtained here and those reported in literature in terms of onset potential and half-wave potential in addition to mass activity can be added.
8. For practical application in fuel cells, longer consecutive cycling stability needs to be performed and investigated.

Reviewer #2 (Remarks to the Author):

The manuscript entitled "Alloying–Realloying Enabled High Durability for Pt–Pd–3d-Transition Metal Nanoparticle Fuel Cell Catalysts" by Wu et al. demonstrated a highly-durable alloy catalyst derived by alloying PtPd (<50%) with 3d-transition metals (Cu, Ni or Co) in ternary compositions. In comparison to the published results about dealloyed ORR catalysts, I think that the main difference is the authors state a dealloy-realloy process is resulting in the more stable catalyst. Thus, the authors should give a detailed explanation of the origin of re-alloy process. Specifically, why does the addition of Pd to PtCu induce the re-alloy under electrochemical conditions as previously reported results (e.g. PtCu) showed the only de-alloy process. Although the authors explain the reason of enhanced stability for Pt20Pd20Cu60 by theoretical calculations, the role of Pd should be further analyzed and discussed in promoting re-alloying. How about other precious metals such as Rh, Ir, Ru, Au etc? Perhaps a general theory about the phenomenon should be deduced.

Also, the statement of "Kinetic currents extracted from rotating disc electrode (RDE) curves (Fig. 2a, b) showed a maximum of MA (1.66 A/mgPt) for Pt20Pd20Cu60/C, almost a 10-fold increase over the state-of-the-art commercial Pt/C catalyst (0.22 A/mgPt)." on page 5 is not correct.

The reviewer thinks that it is suitable to publish on this journal with the above concerns are addressed.

Reviewer #3 (Remarks to the Author):

The authors present a systematic investigation of a family of ternary PtPdCu alloy catalyst nanoparticles for the oxygen reduction reaction with focus on their structure evolution under electrochemical cell or fuel cell operating conditions using advanced characterization techniques and modelling.
They discuss a fundamental question in the durability of Pt-alloy nanoparticles under fuel cell

operating conditions: “how a low-noble-metal-content (< 50 at.%) catalyst with high percentages of dealloyable metals can remain at active alloy state”, which is of a high significance for the understanding of the stability of these catalysts in fuel cell cathodes. The key result is the proposed process of dynamic realloying as a new stabilization strategy.
The characterization and modelling by HE-XRD and PDF analysis is impressive and surely show the power of this approach in investigating catalysts under operation, which will be of great interest to the nanoparticle catalysis community. However, I have found what I believe is the main result of the paper not completely supported by experimental results. If the realloying process were stronger supported by structural data, I would recommend the publication in this Journal. Therefore, at the present version I recommend revision and submission to another journal.

Major Comments:

• If I am correct, the authors suggest the following process to occur: a “homogeneous” random PtPdCu alloy goes through the dealloying of small amount of Cu during the first cycles of stability tests (based on cyclic voltammetry only information) and then realloying occurs till a new stable alloy is formed (based on final elemental mapping and PDF showing basically no PGM skin). The first question that is not clear to me is why this new state is more stable than the initial one to prevent further dealloying. More importantly, the authors state: “we demonstrate for the first time dynamic realloying as the pathway towards self-healable durability”. However, I cannot find any structural evidence (from PDF or elemental mapping) of the intermediate in situ dealloyed state. All the structural data, i.e. Figure 4f, point to a monotone slow process from initial to final state, which is in contrast with the authors presented process of realloying.
• Page 17, line 640 and Supplementary Figure 13a: “The Pt loading on the carbon papers for pure commercial Pt/C catalyst was about 0.08 mgPt/cm2 while for Pt20Pd20Cu60/C ORR catalyst was around 0.6-0.8 mgPt/cm2 ”. Up to 10 times more Pt has been used for fabrication of the PtPdCu MEA, but the mass activity at 0.9 V is not reported. The authors wrote only a general comment in the main text (page 12, line 439): “In addition to outperforming the commercial Pt/C catalyst under the same condition…” and refer to Supp Figure 13a. In Fig 13a, the polarization curves are reported as mA/cm2/mgPt, indicating a mass normalization, so I think that the data can be easily provided by the authors, but it is not easy to extract by the reader. Also, would the PtPdCu outperform the Pt/C also considering PGM mass activity? The authors should provide both Pt and PGM mass activity at 0.9 V in comparison to Pt/C and be more specific in the statement at page 12, line 439.

Other Comments:

• Supplementary Figure 1: The figure seems to show that the high Pt at% in the nanoparticles is the problem for high price. Instead of the Ptat%, it is the Pt weight loading on the electrode that is important. This should be clarified. One can have high Pt at% nanoparticle but super active catalyst ,allowing a low loading, i.e. in other words mass activity is calculated on the total Pt on the electrode, not on the at% Pt. So Figure S1a should be revised.
• Supplementary Figure 7cd: It is not clear to me what is the difference between Fig. 3c, d and Supplementary Fig. 7c, d. Are they just two different nanoparticles at the same conditions? Also, if the author can better specify in the caption of Figure 3a, b that with as-prepared they mean heat-treated and electrochemically activated, it will help the reader.
• Page 2 line 48: “Nevertheless, the dealloying approach to catalyst preparation, which dates back decade ago without real breakthroughs in commercializing alloy catalysts, has shown impracticality for operation in fuel cells2.” I believe that the authors here mean electrochemically dealloyed catalysts, not simply and generally dealloyed catalysts. This specification “electrochemically” or “voltammetric” should be inserted.
• Page 2 line 53: “and almost all perform poorly in real fuel cells15.”The authors cite a perspective from 2016, which might be not up-to-date. More recent reviews or perspective on the topic will show a more up-to-date (even though I suspect that the conclusion will be the same, so no changes of the sentence will probably be required).

• Page 3 line 109: "none of them have achieved a real breakthrough in understanding the evolution of the nanophase structures at the atomic scale." Do the authors mean the evolution under operating conditions? I believe that this sentence should be clarified by adding "under operation" or similar expressions.
• Page 7, line 278: "the as-synthesized Pt20Pd20Cu60 NPs (Supplementary Fig. 6a, b". I think that the referred figure should be Supplementary Fig. 7a, b, not 6.
• Page 7 line 287: "While it is feasible that a further examination of the very small percentage change of the dissolved metal ions in the RDE experiment, which is even smaller in MEA in the experimental time scale as discussed later, by ICP-OES or in-situ ICP-mass pectroscopy36 could gain some information on the transient behavior." It seems it misses the main clause. Please revise the sentence.
• Page 10, Line 390: "This structurally super stable alloy catalyst is in sharp contrast those catalysts". Missing the preposition of in contrast, i.e. in contrast to.
• Page 15, line 553 "Commercial Pt/C catalyst (E-tek 20 wt% loading)". I could not find the Pt and PGM wt% loading on carbon for RDE and MEA of the PtPdCu. Could the authors add it?
• Page 17, line 662 "the deformed cell sealing affect". Effect?

Reviewer #4 (Remarks to the Author):

The manuscript from Wu and co-workers reports a very impressive dataset from numerous characterization techniques and computational models regarding the high durability of PtPdCu nanoparticles in fuel cell cathode conditions. Whereas the work is quantitative, one can have concerns about some experimental details that lead the authors to their conclusions. This work could be published in Nature Communication, if the following comments are properly addressed:

General:
-In this study, but also in a previous one including a part from the co-authors (J. Am. Chem. Soc. 2020, 142, 1287−1299), the high stability is addressed using a 100 mV/s 0.6-1V. This protocol is known to induce only minor changes compare to a square wave-type potential profile, because of the short time polarization spent ~1V vs. RHE. Besides, this high sweep rate magnifies the ohmic drop losses, which means the catalyst is not even really polarize to the 1V upper potential limit. The fact that the first 20k cycles in liquid electrolyte actually further condition/activate the material (both SA and MA increases) supports its poor damaging power. It reads also strange, even suspicious that the comparison with the reference Pt material is stopped after 20k cycles. In case where Pt further degrades after 20k cycles, it would be only more valuable to the manuscript. These data should be included.
These doubts about the significance of the AST are particularly problematic considering that the stability of the introduced PtPdCu material is the main topic of the present contribution. More specifically, in the case of the operando experiment conducted here, the authors confess (and this is really welcomed) the cell used features high internal resistance that distorts the CVs even at lower sweep rate.

-It is not clear why the 'realloying' occurs in the PtPdCu system and not in any binary or ternary alloys. Whereas the chemical composition is not directly accessible in Fig. 3, one can see the fraction of Cu is diminished in the core. Since these PtPdCu particles are particularly Cu-rich (60 %), it seems that simply further cycling (and/or the use of a real degrading AST) would dissolve all the Cu stock, and the PdPtCu materials eventually suffer from the 'usual' non-noble metal losses. Continuous leaching of the non-noble metal is known to damage the ionic conductivity of the surrounding ionomer/membrane and is not desirable in fuel cell system. Which is typically, why literature focused during the past decade on core-shell materials that tentatively protect the non-noble core from dissolution. In other words, if there is 'realloying' it means there was dealloying (of course surface Cu

is not stable in PEMFC cathode conditions), the final loss in Cu within the NP indicates that Cu is eventually lost, and the realloying process will eventually stop. Again, the fast sweep rate used for the AST mitigate the Cu diffusion far from the material, which would typically occur in real PEMFC operation
More specific:

-l 48-50: The authors claim that the dealloyed approach to catalyst preparation never showed significant commercial breakthroughs and are impracticable for operation in fuel cells, based on the citation of a paper from 2012. Actually, core-shell type PtCo catalyst is used in the Toyota Mirai (Toshihiko Yoshida and Koichi Kojima 2015 Electrochem. Soc. Interface 24 45; Yuefei Cui et al 2020 J. Electrochem. Soc. 167 064520). This statement should be removed.

-l 108-110: If the field of fuel cell electrocatalysis may be competitive, and one may have to defend the originality/significance of his/her work, the practice consisting in the depreciation of previous works conducted by other groups is really not welcome in the scientific community. Saying that no one of the previous studies has ever achieved a breakthrough in the understanding of nanophase structures evolution at the atomic scale is completely unacceptable. Does this imply the present manuscript is the first one to achieve this?
-Fig. 3: It is not clear how the intensities of the STEM-EELS elemental line scans are normalized, thus the surface (and bulk) compositions are hard for the reader to evaluate.

L 374-375: The authors claim no PGM-skin was detected from XRD. What would have been the signature of a Pt, Pd or PtPd-rich shell among the disordered alloy? Especially on these rather large particles >6nm were the surface contribution compared to the bulk is low. The strained shell would not typically produce Bragg reflexions at different angles, and a different interatomic distance not detectable in PDF analyses. It does not sound like a valid argument. EXAFS would be more suited than XRD to tackle this question.

Point-by-point responses to the reviewers' comments, questions and suggestions, and the revisions made in the manuscript (NCOMMS-20-31672B):

Reviewer #1:

This manuscript by Wu et al. demonstrated a durable ternary alloy catalyst by alloying PtPd (<50%) with Cu (Ni or Co). Dealloying-realloying of the alloy catalysts was observed during potential cycles by in-situ/operando synchrotron X-ray diffraction spectroscopy in combination with STEM/EELS and XPS analyses, as well as DFT modeling. The results are interesting and may provide useful information for design of stable and active alloy catalysts in fuel cells. Before possible consideration of acceptance in Nat. Commun., the following issues should be clarified.

Response: We thank the reviewer's appreciation on our results for catalyst design in fuel cells. The issues raised by the reviewer and the valuable comments are addressed in the following responses.

Comment 1. The abbreviation MA should be defined when first used.

Response: Thank you for pointing it out. The abbreviation of mass activity (MA) is defined in the revision on page 2 as highlighted in yellow.

Comment 2. Corresponding metric should be added to the Y-axis of the top panel in Fig. 1b.

Response: Thank you for the suggestion. The corresponding metric is added to the Y-axis of the top panel in Fig. 1b in the revised version.

Comment 3. Did some Pd species as well as Cu leach into the electrolyte?

Response: Thank you for the question. As discussed in the manuscript, we performed composition analysis of the composition change. In comparison with the amount of Cu species being leached into the electrolyte, there was a relatively smaller portion of Pd which was also dissolved into the electrolyte during the prolonged potential cycling. This is in fact similar to the dissolution phenomenon for Pt species upon long duration potential cycling in fuel cells. In the revision, we clarified this point in the manuscript (Page 8).

Comment 4. The evolution kinetics of re-alloying during ADT tests can be investigated.

Response: Thank you for the comment/suggestion. Yes, we had some discussion of it in our original manuscript. The evolution kinetics of the lattice constant during the in-situ experiment generally was shown to follow the first order reaction kinetics with an apparent rate constant of $\sim 3\times10^{-5}$ s^{-1} and a final lattice parameter of 3.765 Å after the 20,000 cycles (Lines 394–396 on Page 10 in the original manuscript). The average lattice constants generally obey this first order kinetics as shown in Supplementary Fig. 12d in the revision. In fact, we further investigated the evolution kinetics of realloying during the ADT test by analyzing the dealloying-realloying details in the in-situ HE-XRD/PEMFC data. As shown by the result shown in Supplementary Fig. 12c added in the revision, the PDF peak and the calculated lattice constant are found to display a regular/irregular oscillatory kinetics as a function of the number of potential cycling. This phenomenon is believed to reflect dealloying–realloying induced changes in lattice constant, i.e., lattice constant expands upon dealloying and shrinks upon realloying. This finding is consistent with the finding with other nanoalloys [Ref 27 in the revision, *Nanoscale* **8**, 10749 (2016)]. Interestingly, the average frequency for the oscillatory realloying kinetics appears to fall in the range of self-diffusion of metal atoms in the surface/subsurface layers of the nanoparticles. This is supported by considering the diffusion coefficients for Pt and Cu atoms based on recent studies of the surface diffusion of atoms on Pt, Cu, and Au nanoparticles [Ref 25 and Refs 89-92 in Supplementary Information, *Sci. Rep.* **4**, 6765 (2014); *Nano Lett.* **12**, 6071 (2012); *Part. Part. Syst. Charact.* **32**, 373 (2015); *Micron* **63**, 52 (2014)]. We added a discussion on the above analysis result in the revised manuscript (Pages 10-11) and in the Supplementary Information (Pages S2-3).

Comment 5. To attain a stable Pt-based ternary alloy catalyst, what's the rule for selection of the second and third metal components? Further, is it possible to design an active ternary alloy ORR electrocatalyst without use of Pt based on this dealloying-re-alloying finding?

Response: We appreciate very much the comment and questions. In terms of the selection of the second and third metal components, we did have some discussion in the original manuscript on Page 3 (Lines 119-142). In this work, we focused on PtPd-based catalysts alloyed with base metals. Pd was chosen as a Pt's partner noble metal component because i) catalytic synergy enabled by the dual-noble-metal character comparing with single noble metal counterparts, as demonstrated in our studies of PtPd/C NP and NW catalysts [*J. Phys. Chem. C* **121**, 14128 (2017); *Nano Lett.* **20**, 2416 (2020)], ii) Pd, like Pt, is resistant to acid corrosion, iii) Pd is more malleable than Pt, helping self-healing upon dealloying, and iv) Pd's oxidation potential is lower than Pt, helping form a passivation. These contribute to the enhanced stability. For the third metal component, the selection of a non-noble transition metal (e.g., Cu, Co, Ni, Fe, etc.) stems first from the reduction of the catalyst cost. Synoptically, this metal plays a central role in the catalytic activity and stability by alloying effect reflected by the optimization of both electronic and strain effects. As to the possibility of designing an active ternary alloy ORR electrocatalyst without use of Pt based on this dealloying-re-alloying finding, we agree that is possible, and it's definitely part of our goal in future work. In fact, there are studies showing that Pt-based alloy nanomaterials could provide high-entropy, robust and anti-corrosive properties in addition to high intrinsic catalytic activity. Nevertheless, our work opens a new avenue to develop efficient ternary or multimetallic catalysts combining Pt, other noble metals, and different base metals. In the revised version, we have re-emphasized or reflected the above attributes (Pages 3 and 15).

Comment 6. From the line-scan profiles shown in Fig. 3c and d, the composition of the two nanoparticles are not complete homogeneous. The authors need to comment this.

Response: Thank you for the comment and suggestion. There are two related aspects in clarifying this point. First, the STEM-EELS line-scan profile result is highly selective based on a limited number of nanoparticles, rather than the statistically-averaging result as provided by XRD. The resolution of the line-scan profiles is relatively low, as limited by the instrument that was used. Second, the homogeneous element distribution of the ternary nanoparticles is evidenced by the XRD and HE-XRD/PDF results, showing no indication of phase segregation. In addition, the formation of completely homogeneous alloy for the as-synthesized NPs is controlled by nucleation and growth rates of different metals during the synthesis. The subsequent thermochemical treatment was used to calcine the NPs, favoring the formation of homogeneous alloy through structure optimization. The presence of incomplete homogenization, while insignificant, was also noticed when selected NPs were analyzed. In the revised version, we have reflected this point (Page 8).

Comment 7. Comparisons between the ternary catalyst obtained here and those reported in literature in terms of onset potential and half-wave potential in addition to mass activity can be added.

Response: Thank you for the suggestion. We have added a comparison table as Supplementary Table 4 in the revision, which compares the performances of ternary ORR electrocatalysts recently reported in the literature.

Comment 8. For practical application in fuel cells, longer consecutive cycling stability needs to be performed and investigated.

Response: Thank you for the comment and suggestion. We agree that longer cycling stability needs to be studied for practical application in fuel cells. In this work, our focus is the understanding of the role of realloying in the catalysts under fuel cell operation conditions. Since the most significant performance variation occurs during the initial stage of the fuel cell operation, we stated in the original manuscript on Page 18 (Lines 679-680) "Prior to the durability test, a current density of about 2.15 $A/cm^2/mg_{Pt}$ (0.32 A/cm^2) was applied to the fuel cell for about 16 hours until the cell voltage reached to a stable value", and we demonstrated the stability in Supplementary Fig. 13 with negligible cell voltage change during 72 h

fuel cell operation. At the reviewer's suggestion, we doubled the time length up to 160 hours in the fuel cell preformation evaluation to support the high stability for potential application. The result, which is added in Supplementary Fig. 13 in the revision, has further substantiated our conclusion on the catalyst's performance in terms of stability.

Reviewer #2:

The manuscript entitled "Alloying–Realloying Enabled High Durability for Pt–Pd–3d-Transition Metal Nanoparticle Fuel Cell Catalysts" by Wu et al. demonstrated a highly-durable alloy catalyst derived by alloying PtPd (<50%) with 3d-transition metals (Cu, Ni or Co) in ternary compositions. In comparison to the published results about dealloyed ORR catalysts, I think that the main difference is the authors state a dealloy-realloy process is resulting in the more stable catalyst. Thus, the authors should give a detailed explanation of the origin of re-alloy process.

Response: We first thank the reviewer's recognition of our finding on the dealloying-realloying enabled stability. As to the origin of the realloying process, in addition to what we have discussed in the original manuscript, we would like to further emphasize that this process is supported by the detailed analysis of the in-situ HE-XRD/PEMFC data. As shown in Supplementary Fig. 12c in the revision, an oscillatory behavior of the lattice constants during the potential cycling is clearly revealed. The observed lattice constant expansion and shrinkage reflect the dealloying and realloying processes, respectively. In fact, we looked into the diffusion coefficients reported for atoms on Pt and Cu nanoparticle surface, which are in the range of e.g., 1×10^{-18} to 1×10^{-16} cm^2/s [Ref 25 and Refs 89-92 in the Supplementary Information, *Sci. Rep.* **4**, 6765 (2014); *Nano Lett.* **12**, 6071 (2012); *Part. Part. Syst. Charact.* **32**, 373 (2015); *Micron* **63**, 52 (2014)]. This is many orders of magnitude greater than the bulk counterparts. We estimated the approximate diffusion time with respect to the average particle size. The result indicated that in the oscillatory peak-to-valley time frame (800 ~1200 s) the estimated diffusion distance would be 0.4 ~ 4.8 nm, which is in a close agreement with the NP size. With the help of this additional insight, the origin of the realloying phenomenon can be summarized in terms of several driving forces: i) the greatly increased atomic mobility at the nanoscale (many orders of magnitude than the bulk materials); ii) the combination of the applied electrochemical potential which induces the atomic rearrangement ($\Delta G = -nFE$) and the negative enthalpy change ($\Delta H < 0$) at the nanoscale as known for many nanoscale alloy particles [Refs 23, 26 in the revision, *Acc. Chem. Res.* **49**, 1587 (2016), *Acc. Chem. Res.* **53**, DOI: 10.1021/acs.accounts.0c00564 (2020), and *Nano Lett.* **12**, 4289 (2012)] which would favor realloying towards a thermodynamically stable state, and iii) the parting limit for the alloying components to allow their co-existence in/on the nanoalloy. To provide a more detailed explanation of the origin of realloying process, we reflected the above discussion in the revised version (Page 11 and 15).

Comment 1. Specifically, why does the addition of Pd to PtCu induce the re-alloy under electrochemical conditions as previously reported results (e.g. PtCu) showed the only de-alloy process. Although the authors explain the reason of enhanced stability for Pt20Pd20Cu60 by theoretical calculations, the role of Pd should be further analyzed and discussed in promoting re-alloying.

Response: Thank you for the question and comment. First, Pd, as a Pt's partner noble metal component, is believed to provide: i) catalytic synergy enabled by the dual-noble-metal character comparing with the single noble metal counterpart, as demonstrated in our studies of PtPd/C NP/NW catalysts [*J. Phys. Chem. C* **121**, 14128 (2017); *Nano Lett.* **20**, 2416 (2020)], ii) the resistance to acid corrosion, iii) the enhanced malleability helping self-healing upon dealloying, and iv) the lower oxidation potential helping form a passivation. Secondly, the realloying phenomenon is also known to occur in recent studies of PtCu and PtFe nanocatalysts, except showing a higher percentage of the dealloying in the realloyed structures [Refs 31, 40 in the revision, *Angew. Chem. Int. Ed.* **59**, 13778 (2020); *J. Am. Chem. Soc.* **142**, 1287 (2020)]. As revealed by the oscillatory kinetics in Supplementary Fig. 12c in the revision, the realloying is highly dynamic process [see, e.g., *Acc. Chem. Res.* **53**, DOI: 10.1021/acs.accounts.0c00564 (2020); *Nanoscale* **8**, 10749 (2016)]. Most previous studies only reported "conditioned" catalysts after the activation by ex-situ

techniques, which are unable to capture this dynamic process. In the dealloying-realloying process, Pd helps promote the realloying process and enhance the stability by reducing the dealloying degree. In addition, the ternary alloy composition with the added Pd increases the binding strength and the entropy of nanoparticles, and further enhances the stability. To address the question, the above discussion is reflected in the revised manuscript (Pages 3 and 15).

Comment 2. How about other precious metals such as Rh, Ir, Ru, Au etc? Perhaps a general theory about the phenomenon should be deduced.

Response: We appreciate this valuable comment. In general, we believe that similar or subtly-different synergies may operate for nanoalloy catalysts containing other precious metals such as Ir, Au, Ru, etc., depending on the actual compositions and phase structures. In fact, the incorporation of other precious metals into Pt-based alloy has been studied in some of our previous and recent works, e.g., PtAu nanowires [*J. Am. Chem. Soc.* **138**, 12166−12175 (2016)], PtIr-based ternary nanoparticles [*ACS Catal.* **1**, 562–572 (2011)], and PtRu nanoparticles [*J. Phys. Chem. C* **121**, 17077–17087 (2017)], but not by in-situ studies as in this work. Nevertheless, our work opens a new avenue to develop other potentially efficient ternary, multimetallic, and high-entropy-alloy catalysts combining Pt, other noble metals, and base metals. This is part of our on-going research topics. The above discussion is reflected in the revision (Page 15).

Comment 3. Also, the statement of "Kinetic currents extracted from rotating disc electrode (RDE) curves (Fig. 2a, b) showed a maximum of MA (1.66 A/mgPt) for Pt20Pd20Cu60/C, almost a 10-fold increase over the state-of-the-art commercial Pt/C catalyst (0.22 A/mgPt)." on page 5 is not correct.

Response: Thank you for pointing this out. To be more rigorous in terms of numbers, this statement has been revised as "Kinetic currents extracted from rotating disc electrode (RDE) curves (Fig. 2a, b) showed a maximum of MA (1.66 A/mg_{Pt}) for $Pt_{20}Pd_{20}Cu_{60}/C$, which is 9-fold increase over the commercial Pt/C catalyst (0.18 A/mg_{Pt})[40]." (Page 5).

Reviewer #3:

The authors present a systematic investigation of a family of ternary PtPdCu alloy catalyst nanoparticles for the oxygen reduction reaction with focus on their structure evolution under electrochemical cell or fuel cell operating conditions using advanced characterization techniques and modelling.

They discuss a fundamental question in the durability of Pt-alloy nanoparticles under fuel cell operating conditions: "how a low-noble-metal-content (< 50 at.%) catalyst with high percentages of dealloyable metals can remain at active alloy state", which is of a high significance for the understanding of the stability of these catalysts in fuel cell cathodes. The key result is the proposed process of dynamic realloying as a new stabilization strategy.

The characterization and modelling by HE-XRD and PDF analysis is impressive and surely show the power of this approach in investigating catalysts under operation, which will be of great interest to the nanoparticle catalysis community. However, I have found what I believe is the main result of the paper not completely supported by experimental results. If the realloying process were stronger supported by structural data, I would recommend the publication in this Journal. Therefore, at the present version I recommend revision and submission to another journal.

Response: We appreciate the reviewer's recognition of the powerfulness and value of HE-XRD/PDF analysis to the nanoparticle catalysis community. We also appreciate the comment on asking for detailed experimental results supporting the realloying process, which are provided in our responses to Comment 1.

Comment 1. If I am correct, the authors suggest the following process to occur: a "homogeneous" random PtPdCu alloy goes through the dealloying of small amount of Cu during the first cycles of stability tests (based on cyclic voltammetry only information) and then realloying occurs till a new stable

alloy is formed (based on final elemental mapping and PDF showing basically no PGM skin). The first question that is not clear to me is why this new state is more stable than the initial one to prevent further dealloying. More importantly, the authors state: "we demonstrate for the first time dynamic realloying as the pathway towards self-healable durability". However, I cannot find any structural evidence (from PDF or elemental mapping) of the intermediate in situ dealloyed state. All the structural data, i.e. Figure 4f, point to a monotone slow process from initial to final state, which is in contrast with the authors presented process of realloying.

Response: Thank you for the questions and comments. Yes, PtPdCu/C catalyst undergoes a quick initial reconstruction upon leaching out accessible surface Cu species under the initial potential cycles (~ 20 cycles), and the subsequently-realloyed state is more stable than the initial stage to prevent further dealloying. The factors controlling this process are discussed in the manuscript based on several experimental results. There is a relative surface enrichment of noble metal, e.g., $Pt_{35}Pd_{23}Cu_{42}$ revealed by RMC modeling based on the HE-XRD/PDF data, and $Pt_{40}Pd_{20}Cu_{40}$ revealed based on the Ar-sputtering experiment, in comparison with the initial composition ($Pt_{19}Pd_{19}Cu_{62}$). Realloying continuously played a very important role in reaching the stable state. This process is highly dynamic. To further aid the understanding of the dynamic dealloying-realloying process, we have included the detailed analysis of the lattice constant changes during the in-situ potential cycling experiment, as shown in Supplementary Fig. 12c in the revision. As stated in our earlier responses, the PDF peak and the calculated lattice constant display a regular/irregular oscillatory kinetics as a function of the number of potential cycling, reflecting dealloying-realloying induced subtle structural changes in the intermediate states, i.e., lattice constant expands upon dealloying and shrinks upon realloying. This finding is consistent with findings for other nanoalloys [Ref 27 in the revision, *Nanoscale* **8**, 10749–10767 (2016)]. Interestingly, the approximate frequency for the oscillatory realloying kinetics appears to fall in the range of self-diffusion of metal atoms in the surface/subsurface layers of the nanoparticles. This is supported by considering the diffusion coefficients for Pt and Cu atoms based on recent studies of surface atomic diffusion on Pt, Cu, and Au nanoparticles [Ref 25 and Refs 89-92 in the Supplementary Information, *Sci. Rep.* **4**, 6765 (2014); *Nano Lett.* **12**, 6071 (2012); *Part. Part. Syst. Charact.* **32**, 373 (2015); *Micron* **63**, 52 (2014)]. As for the elemental mapping, we would like to point out that the information provided by the elemental mapping is largely based on highly-selective and very limited number of nanoparticles, which does not necessarily represent the overall average dynamic structure evolution as revealed by the in-situ HE-XRD/PDF. The above discussion is reflected in the revised manuscript (Pages 10-11).

Comment 2. Page 17, line 640 and Supplementary Figure 13a: "The Pt loading on the carbon papers for pure commercial Pt/C catalyst was about 0.08 mgPt/cm2 while for Pt20Pd20Cu60/C ORR catalyst was around 0.6-0.8 mgPt/cm2 ". Up to 10 times more Pt has been used for fabrication of the PtPdCu MEA, but the mass activity at 0.9 V is not reported. The authors wrote only a general comment in the main text (page 12, line 439): "In addition to outperforming the commercial Pt/C catalyst under the same condition…" and refer to Supp Figure 13a. In Fig 13a, the polarization curves are reported as mA/cm2/mgPt, indicating a mass normalization, so I think that the data can be easily provided by the authors, but it is not easy to extract by the reader. Also, would the PtPdCu outperform the Pt/C also considering PGM mass activity? The authors should provide both Pt and PGM mass activity at 0.9 V in comparison to Pt/C and be more specific in the statement at page 12, line 439.

Response: Thank you for the comment and question. We would like to point out that the mass loading in the custom-designed PEMFC cell for in-situ HE-XRD is different from that in the standard PEMFC for considering the requirement for the in-situ HE-XRD experiment. The X-ray penetrates through the MEA, producing the catalyst information on both cathodic and anodic sides. For this reason, we design a much higher Pt loading of the alloy catalyst on the cathode side (almost a 10-fold Pt mass loading) than the anode side so that the collected information reflects mainly the cathode catalyst. The fuel cell performance test as shown in Supplementary Fig. 13 was conducted in a standard PEMFC using $Pt_{20}Pd_{20}Cu_{60}$/C (0.15 mg_{Pt}/cm^2) as the cathode catalyst and commercial Pt/C (0.40 mg_{Pt}/cm^2) as the anode catalysts. While the mass activity at 0.9 V for the fuel cell is low due to limited optimization of the

engineering factors of the fuel cell, a known issue in the fuel cell research community, the mass activity at 0.8 V, 0.86 A/mg_{Pt}, or 0.54 A/mg_{PGM} based on the total mass of all PGMs without iR compensation, clearly outperforms that of the commercial Pt/C catalyst (0.29 A/mg_{Pt}) in the fuel cell under the same operating condition. We expect that a better fuel cell performance could be achieved upon further optimization of the engineering factors in future work. We have more specifically reflected the above comments in the revised version (Pages 13 and 18).

Comment 3. Supplementary Figure 1: The figure seems to show that the high Pt at% in the nanoparticles is the problem for high price. Instead of the Pt at%, it is the Pt weight loading on the electrode that is important. This should be clarified. One can have high Pt at% nanoparticle but super active catalyst ,allowing a low loading, i.e. in other words mass activity is calculated on the total Pt on the electrode, not on the at% Pt. So Figure S1a should be revised.

Response: Thank you for the comment. We agree that Pt weight loading on the electrode is important. Our use of Pt content in the atomic percent (at.%) is for the composition comparison reason, as widely used in the literature in terms of the atomic ratios instead of mass ratios. One can easily convert atomic percentage to mass percentage, and for better clarity, we have provided both in the revised version (Page S4 in the Supplementary Information).

Comment 4. Supplementary Figure 7cd: It is not clear to me what is the difference between Fig. 3c, d and Supplementary Fig. 7c, d. Are they just two different nanoparticles at the same conditions? Also, if the author can better specify in the caption of Figure 3a, b that with as-prepared they mean heat-treated and electrochemically activated, it will help the reader.

Response: Thank you for the question. As you correctly stated, Fig. 3c, d and Supplementary Fig. 7c, d show the results for two sets of different nanoparticles under the same condition as that for the 20,000-cycled $Pt_{20}Pd_{20}Cu_{60}/C$ catalyst. For better clarity, we added more detailed sample description in the caption of Figure 3a, b. "**a, c,** as-prepared (thermochemically calcined) and **b, d,** after 20,000 cycles (electrochemically cycled) by accelerated durability test in a standard electrochemical cell."

Comment 5. Page 2 line 48: "Nevertheless, the dealloying approach to catalyst preparation, which dates back decade ago without real breakthroughs in commercializing alloy catalysts, has shown impracticality for operation in fuel cells2." I believe that the authors here mean electrochemically dealloyed catalysts, not simply and generally dealloyed catalysts. This specification "electrochemically" or "voltammetric" should be inserted.

Response: We thank the reviewer for the comment. We agree with the assessment. Indeed, chemically dealloying approach has been widely used to fabricate Pt-based alloy fuel cell catalysts. However, the fuel cell performance didn't show a real breakthrough in terms of cost and stability toward the mass commercialization. To clarify our point, we have revised this sentence in the revision on Page 2 as: "Electrochemical dealloying approach to catalyst preparation, which dates back decade ago without breakthroughs in commercializing alloy catalysts in terms of cost and stability, has shown limited success for operation in fuel cells[2,19,20]."

Comment 6. Page 2 line 53: "and almost all perform poorly in real fuel cells15."The authors cite a perspective from 2016, which might be not up-to-date. More recent reviews or perspective on the topic will show a more up-to-date (even though I suspect that the conclusion will be the same, so no changes of the sentence will probably be required).

Response: We appreciate the comment. We have carefully double checked the most recently published papers in this topic area. This remains true as shown by most studies of the ORR performance using electrochemical half-cell, i.e., RDE technique, or in alkaline electrolyte. A recent example involves MOF-derived Pt_3Co catalyst [Ref 10, *Science* **362**, 1276 (2018)] that shows high mass activity in PEMFC but limited stability (85% MA decay after 30K cycles for the LP@PF-2 catalyst). The durability is a longstanding challenging problem in the field of fuel cell catalyst research.

Comment 7. Page 3 line 109: "none of them have achieved a real breakthrough in understanding the evolution of the nanophase structures at the atomic scale." Do the authors mean the evolution under operating conditions? I believe that this sentence should be clarified by adding "under operation" or similar expressions.

Response: Thank you for the question and suggestion. Yes. For a better clarification, we have modified the sentence in the revision on Page 3: "limited success has been achieved in understanding the evolution of the nanophase structures at the atomic scale, especially under the reaction conditions."

Comment 8. Page 7, line 278: "the as-synthesized Pt20Pd20Cu60 NPs (Supplementary Fig. 6a, b". I think that the referred figure should be Supplementary Fig. 7a, b, not 6.

Response: Thank you for pointing out this typo. We have corrected it accordingly in the revision on Page 8.

Comment 9. Page 7 line 287: "While it is feasible that a further examination of the very small percentage change of the dissolved metal ions in the RDE experiment, which is even smaller in MEA in the experimental time scale as discussed later, by ICP-OES or in-situ ICP-mass pectroscopy36 could gain some information on the transient behavior." It seems it misses the main clause. Please revise the sentence.

Response: Thank you for pointing out this mistake. The sentence is corrected on Page 8: "While it is feasible to detect the very small percentage change of dissolved metal ions in the RDE experiment, which is even smaller in MEA in the experimental time scale as discussed later, measurements by ICP-OES or in-situ ICP-mass spectroscopy could gain some information for assessing the transient behavior."

Comment 10. Page 10, Line 390: "This structurally super stable alloy catalyst is in sharp contrast those catalysts". Missing the preposition of in contrast, i.e. in contrast to.

Response: Thank you for pointing out this typo. It has been corrected accordingly.

Comment 11. Page 15, line 553 "Commercial Pt/C catalyst (E-tek 20 wt% loading)". I could not find the Pt and PGM wt% loading on carbon for RDE and MEA of the PtPdCu. Could the authors add it?

Response: We thank the reviewer for pointing it out, which is our negligence. The corresponding experimental details have been added in the revision on Page 17: "The mass metal loading of Pt-based alloy NP electrocatalysts in this study was 20 ± 2 wt% for the standard electrochemical cell test. A higher mass metal loading of ~35 wt% of the catalyst was used in the custom designed PEMFC for the in-situ HE-XRD experiments and the standard PEMFC experiments."

Comment 12. Page 17, line 662 "the deformed cell sealing affect". Effect?

Response: Thank you for pointing out this typo. Yes, it is effect, which has been corrected in the revision.

Reviewer #4:

The manuscript from Wu and co-workers reports a very impressive dataset from numerous characterization techniques and computational models regarding the high durability of PtPdCu nanoparticles in fuel cell cathode conditions. Whereas the work is quantitative, one can have concerns about some experimental details that lead the authors to their conclusions. This work could be published in Nature Communication, if the following comments are properly addressed:

Response: We appreciate the reviewer's recognition of our findings on the high durability of our PtPdCu nanoparticles in fuel cell cathode conditions. The concerns about some experimental details are addressed in the following responses.

Comment 1. In this study, but also in a previous one including a part from the co-authors (J. Am. Chem. Soc. 2020, 142, 1287−1299), the high stability is addressed using a 100 mV/s 0.6-1V. This protocol is known to induce only minor changes compare to a square wave-type potential profile, because of the

short time polarization spent ~1V vs. RHE. Besides, this high sweep rate magnifies the ohmic drop losses, which means the catalyst is not even really polarize to the 1V upper potential limit. The fact that the first 20k cycles in liquid electrolyte actually further condition/activate the material (both SA and MA increases) supports its poor damaging power. It reads also strange, even suspicious that the comparison with the reference Pt material is stopped after 20k cycles. In case where Pt further degrades after 20k cycles, it would be only more valuable to the manuscript. These data should be included.

These doubts about the significance of the AST are particularly problematic considering that the stability of the introduced PtPdCu material is the main topic of the present contribution. More specifically, in the case of the operando experiment conducted here, the authors confess (and this is really welcomed) the cell used features high internal resistance that distorts the CVs even at lower sweep rate.

Response: We thank the reviewer for the comments and suggestions. While we agree that the durability test protocol (100 mV/s, 0.6-1.0 V) used in our works may not be aggressive comparing with the square wave-type potential profile, we would like to point out that this protocol that we used followed that documented by the U.S. DOE Fuel Cell Technologies Office in "Fuel Cells Multi-Year Research Development and Demonstration Plan". This protocol has been widely used by many research groups in the recent literatures, e.g., *Science* **362**, 1276 (2018), *Nat. Energy* **2**, 17111 (2017), *Science* **354**, 1414 (2016), *Science* **343**, 1339 (2014), etc. The fact that the MA and SA increase in the first 20k cycles serves as a clear demonstration that the activation of the catalysts originates form the dynamic realloying process. This type of activation was also observed in another recent work (*Nat. Energy* 2, 17111 (2017)). The excellent stability of PtPd-based nanoparticle catalysts, especially for $Pt_{20}Pd_{20}Cu_{60}/C$ catalyst, is in sharp contrast to the PdCu nanoparticle, which showed 68% MA decay after only 1K cycles under the same operating condition (see Supplementary Fig. 5c, d). Moreover, the excellent durability of $Pt_{20}Pd_{20}Cu_{60}/C$ catalyst was further substantiated by the actual PEMFC performance data (Supplementary Fig. 13), showing a stable cell voltage close to 0.6 V for at least 160 hours under a current density of 1.0 A/cm^2. We agree that we should include reference Pt data in longer operation time for a better comparison to highlight the high durability of $Pt_{20}Pd_{20}Cu_{60}/C$ catalyst. Indeed, as shown in Supplementary Fig. 5e, f in the revision for the extended potential cycles to 40K for the commercial Pt/C catalyst, there is a 55% decrease for the final MA. We have specifically reflected the above discussion in the revised version (Page 6).

Comment 2. It is not clear why the 'realloying' occurs in the PtPdCu system and not in any binary or ternary alloys. Whereas the chemical composition is not directly accessible in Fig. 3, one can see the fraction of Cu is diminished in the core. Since these PtPdCu particles are particularly Cu-rich (60 %), it seems that simply further cycling (and/or the use of a real degrading AST) would dissolve all the Cu stock, and the PdPtCu materials eventually suffer from the 'usual' non-noble metal losses. Continuous leaching of the non-noble metal is known to damage the ionic conductivity of the surrounding ionomer/membrane and is not desirable in fuel cell system. Which is typically, why literature focused during the past decade on core-shell materials that tentatively protect the non-noble core from dissolution. In other words, if there is 'realloying' it means there was dealloying (of course surface Cu is not stable in PEMFC cathode conditions), the final loss in Cu within the NP indicates that Cu is eventually lost, and the realloying process will eventually stop. Again, the fast sweep rate used for the AST mitigate the Cu diffusion far from the material, which would typically occur in real PEMFC operation

Response: We thank the reviewer for the thoughtful comments. While we would agree with part of the reviewer's reasoning, we would like to state our conclusion on realloying stems largely from the experimental observations. Indeed, realloying should in general occur in all PGM-based alloys under the electrochemical conditions (see *Nat. Energy* **2**, 17111 (2017), and *Acc. Chem. Res.* **53,** DOI: 10.1021/acs.accounts.0c00564 (2020)). The critical question is how it correlates with the degree of dealloying and hence the stability, which is an important focus of our work. In the case of PGM-based alloys with low noble metal content (e.g., < 50 at.%), the dealloying process dominates the initial cycling operation stage. In our comparison of the initial atomic composition ($Pt_{19}Pd_{19}Cu_{62}$), the atomic compositions for the as-prepared ($Pt_{21}Pd_{21}Cu_{58}$), and the post-cycled ($Pt_{27}Pd_{27}Cu_{46}$) determined by EDS,

the results are highly consistent with other characterization results such as ICP and XPS, demonstrating that most of the Cu elements are retained in the catalyst after 20,000 cycles. A great portion of Cu still exists in the NP catalyst after long-term fuel cell operation. This realloying-driven process is further supported by the dynamic oscillatory lattice constant observed in the in-situ potential cycling experiment (see the added Supplementary Fig. 12c in the revision). The PDF peak and the calculated lattice constant display a regular/irregular oscillatory kinetics as a function of the number of potential cycling, reflecting dealloying/realloying induced changes, i.e., lattice constant expands upon dealloying and shrinks upon realloying. The final large compressive strain (> 4% with a lattice constant of 3.765 Å after 20,000 cycles) reflects the existence of a great portion of Cu in the alloy catalyst. We agree that continuous leaching of the non-noble metal would damage the ionic conductivity and is not desirable in fuel cell system, which needs in-depth studies in future work. We believe that the realloying is a continuous dynamic process, the degree of which depends on the composition, parting limit, and thermodynamic factors. For better clarity, we reflect the above discussion in the revision on Pages 3, 9-11.

Comment 3. Lines 48-50: The authors claim that the dealloyed approach to catalyst preparation never showed significant commercial breakthroughs and are impracticable for operation in fuel cells, based on the citation of a paper from 2012. Actually, core-shell type PtCo catalyst is used in the Toyota Mirai (Toshihiko Yoshida and Koichi Kojima 2015 Electrochem. Soc. Interface 24 45; Yuefei Cui et al 2020 J. Electrochem. Soc. 167 064520). This statement should be removed.

Response: We appreciate the reviewer for the valuable comment and mentioning the references. We agree with the reviewer's point. We are aware that PtCo/C catalyst is used in Toyota Mirai. However, we were not clear about the preparation of the core-shell PtCo catalyst in that use. To better reflect the reviewer's concern, we have modified the statement and cited the references in the revision on Page 2: "Electrochemical dealloying approach to catalyst preparation, which dates back decade ago without breakthroughs in commercializing alloy catalysts in terms of cost and stability, has shown limited success for operation in fuel cells[2,19,20]."

Comment 4. Lines 108-110: If the field of fuel cell electrocatalysis may be competitive, and one may have to defend the originality/significance of his/her work, the practice consisting in the depreciation of previous works conducted by other groups is really not welcome in the scientific community. Saying that no one of the previous studies has ever achieved a breakthrough in the understanding of nanophase structures evolution at the atomic scale is completely unacceptable. Does this imply the present manuscript is the first one to achieve this?

Response: Thank you for the comment, and we agree with your point. We must note that our statement about the breakthrough was by no means to depreciate previous works by other groups. Quite to contrary, our statement was meant to expand our thinking on the realloying and structure evolution of the nanoalloy catalysts built upon previous works. Apparently, our statement was not well phrased to that intention. While realizing the fact that the structure evolution and durability of fuel cell catalysts under in-situ/operando operating conditions have seldom been reported, we have rephrased our statement accordingly on Page 3 in the revision: "limited success has been achieved in understanding the evolution of the nanophase structures at the atomic scale, especially under the reaction conditions."

Comment 5. Fig. 3: It is not clear how the intensities of the STEM-EELS elemental line scans are normalized, thus the surface (and bulk) compositions are hard for the reader to evaluate.

Response: Thank you for the comment. For better clarities, we have replotted all line scan profiles. The intensities of three metal elements in the STEM-EELS elemental line scans are normalized, and the corresponding compositions determined by EDS before and after the potential cycling are provided in the figure captions in the revision (Page 9 in the main text and Page S10 in the Supplementary Information). The atomic compositions of the as-synthesized NP, the pristine carbon-supported catalyst, and the post-cycled catalyst determined by EDS are $Pt_{17}Pd_{23}Cu_{60}$, $Pt_{21}Pd_{21}Cu_{58}$, and $Pt_{27}Pd_{27}Cu_{46}$, respectively, which agree well with the results provided by ICP-OES.

Comment 6. Lines 374-375: The authors claim no PGM-skin was detected from XRD. What would have been the signature of a Pt, Pd or PtPd-rich shell among the disordered alloy? Especially on these rather large particles >6nm were the surface contribution compared to the bulk is low. The strained shell would not typically produce Bragg reflexions at different angles, and a different interatomic distance not detectable in PDF analyses. It does not sound like a valid argument. EXAFS would be more suited than XRD to tackle this question.

Response: Thank you for the comment and question. This is a good point, and indeed, traditionally it is difficult for XRD to detect the formation of PGM-skin. However, in our study with a nanoparticle size around 6 nm, the amount of PGM would only allow the formation of ~2 monolayer PGM-skin in a completely phase-segregated fashion with Cu-core@PGM-skin structure, which is illustrated by the shell thickness–composition–particle size correlation in Supplementary Fig. 1b. This type of complete phase segregation would be detectable by XRD, as shown in our previous studies of AuPt nanoparticles that revealed clear splitting of the XRD peaks for Au and Pt phases (Ref 47 in the revision, *Chem. Mater*. 22, 4282 (2010)). This is indeed supported by our extended HE-XRD/PDF analysis results, as shown in Supplementary Fig. 11d, e which reveal poor fitting results with different Cu-core@PGM-shell models. In fact, the XRD patterns shown in Supplementary Fig. 9a for the 20,000-cycled catalyst showed clearly the alloy feature instead of the phase segregation characteristic. The HE-XRD/PDF analysis results as shown in Supplementary Fig. 11e demonstrated the formation of random alloy for the 20,000-cycled catalyst, rather than other types of structures. We agree with the reviewer that EXAFS may help provide some structural information in this aspect, as we did so in previous works [Ref 45 in the revision, *J. Am. Chem. Soc.* 134, 15048 (2012)]. There is however a limit of the insight because of the local structural character in EXAFS, rather than the both local and extended structure information obtainable in HE-XRD/PDF coupled with RMC modeling. This continues to be part of our on-going studies. We have reflected the above discussion in the revision on Pages 10.

Again, we would like to thank all four reviewers for their thoughtful and insightful comments, questions and suggestions.

Reviewer #1 (Remarks to the Author):

The authors have addressed most of my concerns and the revised manuscript can thus be accepted for publication in Nat. Commun.

Reviewer #3 (Remarks to the Author):

The authros improved the manuscript significantly, providing additional missing details related to the fuel cell measurements, clarify some sentences and in particular addressing my main concern by the addition of Figure 12c and related discussion in the main. Based on the new provided insights about the operando realloying process, this work is in my opinion now suitable to be published in Nat Comm.

Reviewer #4 (Remarks to the Author):

The authors have carefully addressed most of the comments. Although some answers may lead to some further questions, this would be interesting discussions in the field.
I recommend publication in Nature Communications
